# Point-of-Care Ultrasound for the Detection of Vascular Access Site Complications—The ULTRASITCOM Study

**DOI:** 10.1016/j.jscai.2024.102516

**Published:** 2025-02-18

**Authors:** Pietro Di Santo, Omar Abdel-Razek, Graeme Prosperi-Porta, Pouya Motazedian, Pascal Thériault-Lauzier, Saad Alhassani, Lee H. Sterling, Simon Parlow, Marie-Eve Mathieu, Richard G. Jung, Baylie Morgan, Doug Coyle, Dean A. Fergusson, Kwadwo Kyeremanteng, Rebecca Mathew, Marino Labinaz, Michael Froeschl, Rebecca Hibbert, Trevor Simard, Jared G. Bird, George A. Wells, Benjamin Hibbert

**Affiliations:** a CAPITAL Research Group, University of Ottawa Heart Institute, Ottawa, Ontario, Canada; b School of Epidemiology and Public Health, University of Ottawa, Ottawa, Ontario, Canada; c Department of Critical Care Medicine, The Ottawa Hospital, Ottawa, Ontario, Canada; d Division of Cardiovascular Medicine, Beth Israel Deaconess Medical Center, Harvard Medical School, Boston, Massachusetts; e Division of Cardiovascular Medicine, Stanford School of Medicine, Palo Alto, California; f Ottawa Hospital Research Institute, Ottawa, Ontario, Canada; g Department of Radiology, Mayo Clinic, Rochester, Minnesota; h Department of Cardiovascular Medicine, Mayo Clinic, Rochester, Minnesota; i Cardiovascular Research Methods Centre, University of Ottawa Heart Institute, Ottawa, Canada

**Keywords:** cardiac catheterization, diagnostic accuracy, point-of-care ultrasound, pseudoaneurysm, vascular access complications

## Abstract

**Background:**

Recent technological advancements have expanded access to ultrasound technology. Invasive cardiac procedures come with risks of vascular access complications, necessitating efficient detection methods for dangerous complications such as pseudoaneurysms. Current clinical practice has relied on physical examination, and often requires formal diagnostic imaging to diagnose these complications. The ULTRAsound Assessment of Access SITe COMplications study assessed the diagnostic accuracy of point-of-care ultrasound (POCUS) as an adjunct to physical examination for the detection of pseudoaneurysms following invasive cardiac procedures.

**Methods:**

We conducted a single-center study that enrolled patients who underwent invasive cardiovascular procedures with suspected access site complications. Cardiology fellows were trained on the use of POCUS by a radiologist with expertise in vascular imaging. The primary outcome focused on the diagnostic odds ratio (DOR) of combined clinical and POCUS assessments compared to Doppler ultrasound or computed tomography.

**Results:**

Among 111 participants, most were female (59.5%), with a mean age of 72.2 years, and with transfemoral access being most prevalent (67.6%). A total of 15 participants were found to have a pseudoaneurysm on formal diagnostic imaging. The combined clinical and POCUS assessments were highly sensitive and demonstrated superior DOR for detecting pseudoaneurysms compared to the physical examination alone (DOR 42.6 [95% CI, 34.6-50.6] vs 15.6 [95% CI, 11.7-19.5]; *P* < .01).

**Conclusions:**

Point-of-care ultrasound is a highly sensitive tool for detecting pseudoaneurysms following invasive cardiovascular procedures. These findings suggest the potential integration of POCUS into routine practice, which could result in timely complication identification and management, thereby improving patient outcomes and reducing health care costs.

## Introduction

Ultrasound is a versatile tool used for diagnosing patients across diverse clinical environments. Its widespread use has been attributed to its notable diagnostic sensitivity, absence of ionizing radiation, minimal invasiveness, and cost-effectiveness.^1^ Recent technological progress has led to the miniaturization of ultrasound components, substantial enhancements in battery longevity, and the creation of compact, high-resolution displays. These advancements have converged to give rise to handheld devices, and when coupled with declining costs, have broadened the accessibility of ultrasonography beyond the boundaries of formal diagnostic imaging departments.^2^

Diagnostic and interventional procedures encompassing coronary, electrophysiological, and valvular interventions are central to the contemporary management of cardiovascular disease. Although radial artery access is associated with improved outcomes and a reduction in complications in patients undergoing percutaneous coronary intervention,^3^^,^^4^ femoral access remains widely utilized, especially for procedures necessitating large-bore sheaths. Although infrequent, these procedures may result in vascular access site complications, primarily pseudoaneurysms, hematomas, arteriovenous (AV) fistulas, dissections, and local stenoses or occlusions related to vascular closure devices. These access site complications have in turn been associated with an extended hospital length of stay, increased patient morbidity and mortality, and increased costs.^5^^,^^6^ Of particular concern, a pseudoaneurysm is a contained rupture of an artery that involves disruption in all 3 layers of the arterial wall and is usually a consequence of an arterial puncture that does not adequately seal.^7^ This allows pulsatile blood to track into the perivascular space and a hematoma with surrounding tissue forms the wall of the pseudoaneurysm. The incidence of this complication is reported to be 2% to 6% following percutaneous coronary intervention.^7^ Although some pseudoaneurysms may thrombose spontaneously, this complication could be life-threatening should the pseudoaneurysm rupture. For many transcatheter procedures, vascular access complications remain the most common complication for affiliated procedures.

Postprocedure evaluation of access site complications is reliant on physical examination and secondarily on formal diagnostic studies in cases in which complications are suspected. The poor diagnostic accuracy of physical examination has been long recognized and has increased physician reliance on formal diagnostic investigations.^8^^,^^9^ Routine use of point-of-care ultrasound (POCUS) at the bedside has become an established standard of care in other aspects of cardiovascular medicine. As a result of a growing body of literature suggesting possible advantages of ultrasonographic assessment as an adjunct to physical examination, we sought to evaluate the ability of POCUS to detect vascular access site complications following invasive cardiac procedures.

## Methods

### Study design

The ULTRAsound Assessment of Access SITe COMplications (ULTRASITCOM) study is a prospective diagnostic accuracy study conducted at a single quaternary care cardiac center in Ottawa, Canada. Participants were recruited over an 18-month period from February 2022 until September 2023.

### Ethical statement

The study was approved by the Ottawa Health Science Network Research Ethics Board and the study was conducted in accordance with the Helsinki Declaration. All study participants provided written informed consent.

### Patient population

Patients who underwent invasive cardiac procedures that required radial, brachial, or femoral arterial and/or femoral venous access and in whom there was a clinical suspicion of an access site complication were considered eligible for the study. In patients with isolated venous access (eg, transcatheter edge-to-edge repair), patients could be included if there was a clinical concern regarding potential arterial injury—venous complications were not specifically addressed in this research study. Patients were excluded if they had hemodynamic instability as defined by mean arterial pressure less than 65 mm Hg or requiring vasopressor support or if they had an urgent or emergent need for surgical intervention as determined by the treating physician.

### POCUS training

General cardiology and interventional cardiology fellows underwent a 2-hour training session with a vascular radiologist which consisted of a didactic teaching session and a case-based review of common pathologies including hematoma, AV fistula, pseudoaneurysm, active extravasation/bleeding, venous and/or arterial thrombosis, edema and lymph nodes. Training sessions were provided every 6 months and fellows were required to attend at least 1 session prior to enrolling their first participant.

### Index and reference tests

Study participants underwent a comprehensive physical examination by a general cardiology or interventional cardiology fellow followed by a POCUS assessment of the access site of concern. A standardized case report form to document the result of their clinical and POCUS assessments was then completed prior to obtaining formal imaging studies. The participant’s electronic medical record was reviewed to obtain baseline demographics, procedural characteristics, the results of the formal diagnostic imaging study, and management of access site complications.

### Study outcomes

The primary outcome of the ULTRASITCOM study was the diagnostic odds ratio (DOR) comparing combined clinical and POCUS assessment to the reference standard of formal diagnostic imaging. The DOR is defined as the ratio of the odds of the test being positive if the subject has a disease relative to the odds of the test being positive if the subject does not have the disease and can be used as a single indicator of test performance.^10^ The value of the DOR ranges from 0 to infinity, with higher values indicating better discriminatory test performance. A DOR of 1 means that a test does not discriminate between patients with the disorder and those without it. The DOR does not depend on the prevalence of the disease that the test is used for. The diagnostic accuracy of the combined physical examination and POCUS assessment was also compared to the physical examination alone by comparing the DOR of each test.

Other measures of diagnostic accuracy including sensitivity, specificity, positive predictive value, negative predictive value, and overall diagnostic accuracy are also reported. Secondary outcome measures included postprocedure management strategies of postprocedure pseudoaneurysms.

### Statistical analysis

Categorical data are expressed as number and percentage and continuous variables are expressed as mean and SD. We analyzed the diagnostic accuracy of the combined clinical and POCUS assessment (ie, index test) in the detection of pseudoaneurysm to formal diagnostic imaging (ie, reference standard) using DOR as the primary measure of test performance. We also evaluated test performance using measures of sensitivity, specificity, positive predictive value, negative predictive value, and overall diagnostic accuracy for the combined assessment as a secondary outcome. Other secondary outcomes evaluating the individual physical examination were analyzed in the same manner as the combined clinical and POCUS assessment. Point estimates and 95% CI for each measure of diagnostic accuracy were calculated. Of note, if a 2 × 2 table contained zeroes, the DOR was undefined, and therefore 0.5 was added to all counts in the table as this is a commonly used method to calculate an approximation of the DOR.^10^

A convenience sample was used without a formal sample size calculation. Given the level of uncertainty regarding the pseudoaneurysm rate, it was not possible to accurately estimate the required sample size. We planned to open recruitment for 18 months with the recruitment of as many participants as possible during this timeframe acknowledging that consecutive patient recruitment was not feasible due to logistical constraints (eg, patients could be referred from the ward or as outpatients without informing the interventional cardiology team). All reported *P* values were 2-sided and a value less than .05 was considered statistically significant. Analyses were performed using SAS version 9.4 (SAS Institute).

## Results

**Figure 1:**
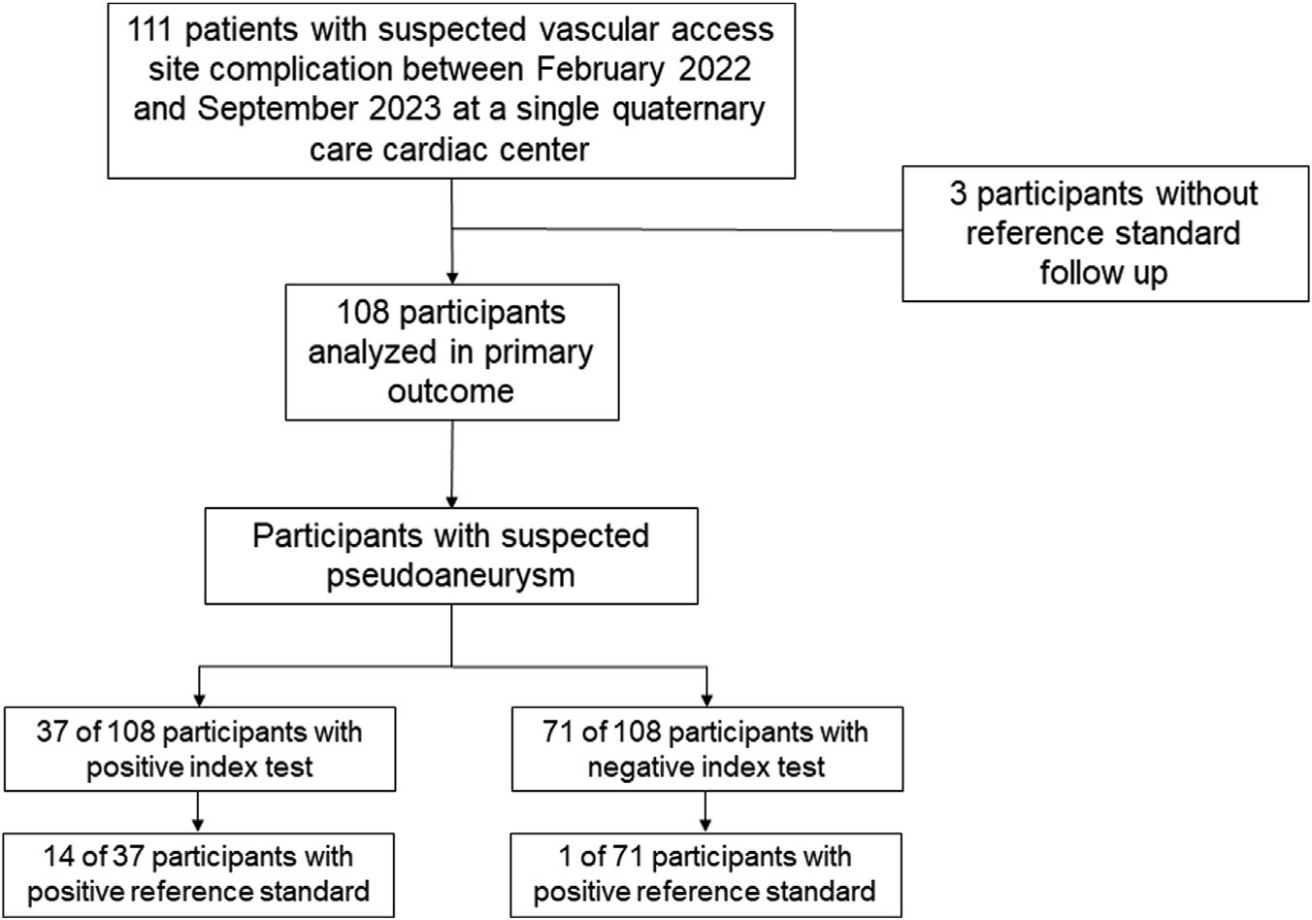
**Study flow.** Flow of participants through the ULTRAsound Assessment of Access SITe COMplications study.

There were 111 study participants enrolled during the study period (Figure 1). All participants underwent detailed clinical evaluation with both physical examination and POCUS assessments (ie, index test). Formal diagnostic imaging with either Doppler ultrasonography or computer tomography (ie, reference standard) was available in 108 study participants (97.3%).

### Participant demographics and procedural characteristics

Most study participants were female (59.5%) with an average age of 72.2 ± 12.5 years, height of 166 ± 10 cm, and weight of 77.3 ± 17.4 kg (Table 1). Cardiovascular risk factors were prevalent with nearly two-thirds of study participants having a known history of hypertension and half with a history of dyslipidemia. Most patients were taking aspirin, P2Y12 inhibitors, beta-blockers, and statins. With respect to procedural characteristics, nearly half of the study participants underwent percutaneous coronary intervention, followed by approximately 25% who had diagnostic coronary angiography alone and the remaining 25% had a structural heart procedure (eg, transcatheter aortic valve intervention, transcatheter edge-to-edge repair, etc). The most common access site of concern was transfemoral (67.6%) followed by transradial (31.5%). Maximum sheath size was most commonly 6F (70.4%) followed by large-bore sheaths (ie, greater than 8F) (18.0%) and nearly half of the study participants had a vascular closure device deployed at the end of the procedure (21.6% with collagen plug-based device and 22.5% with suture-based device).

**Table 1:** Baseline participant demographics and procedural characteristics.

	Study participants (N = 111)
Age, y	72.2 ± 12.5
Female sex	66 (59.5%)
Height, cm	166 ± 10
Weight, kg	77.3 ± 17.4
Past medical history	
Hypertension	70 (63.1%)
Dyslipidemia	58 (52.7%)
Active smoking	16 (14.4%)
Diabetes	38 (34.2%)
Family history of premature CAD	11 (9.9%)
Medications	
Aspirin	79 (71.2%)
P2Y12 inhibitor	75 (67.6%)
Direct oral anticoagulant	12 (10.8%)
Warfarin	0 (0.0%)
ACEI/ARB/ARNI	50 (45.1%)
Beta-blocker	60 (54.1%)
Mineralocorticoid receptor antagonist	15 (13.5%)
SGLT2 inhibitor	16 (14.6%)
Statin	76 (68.5%)
Procedural characteristics	
Diagnostic coronary angiogram	26 (23.4%)
Percutaneous coronary intervention	54 (48.7%)
Transcatheter aortic valve intervention	20 (18.0%)
Transcatheter edge-to-edge repair	2 (1.8%)
Other structural heart intervention	9 (8.1%)
Access site of concern	
Femoral	75 (67.6%)
Radial	35 (31.5%)
Brachial	1 (0.9%)
Maximum sheath size	
6F	76 (70.4%)
7F	3 (2.8%)
8F	9 (8.3%)
9F	14 (13.0%)
10F	0 (0.0%)
11F	2 (1.9%)
12F	4 (3.7%)
Closure device	
None/compression	62 (55.9%)
Angioseal	24 (21.6%)
Proglide	25 (22.5%)

Values are mean ± SD or n (%).

ACEI, angiotensin-converting enzyme inhibitor, ARB, angiotensin receptor blocker, ARNI, angiotensin receptor/neprilysin inhibitor; CAD, coronary artery disease.

### Diagnostic accuracy of index test

Ninety-five study participants (88.0%) underwent Doppler ultrasound assessment whereas 13 participants (12.0%) had computed tomography as the reference standard. The diagnostic test accuracy of the combined clinical and POCUS assessments, as well as the accuracy of physical examination alone, can be found in the Central Illustration, Table 2, and Supplemental Tables S1 and S2.

**Central Illustration:**
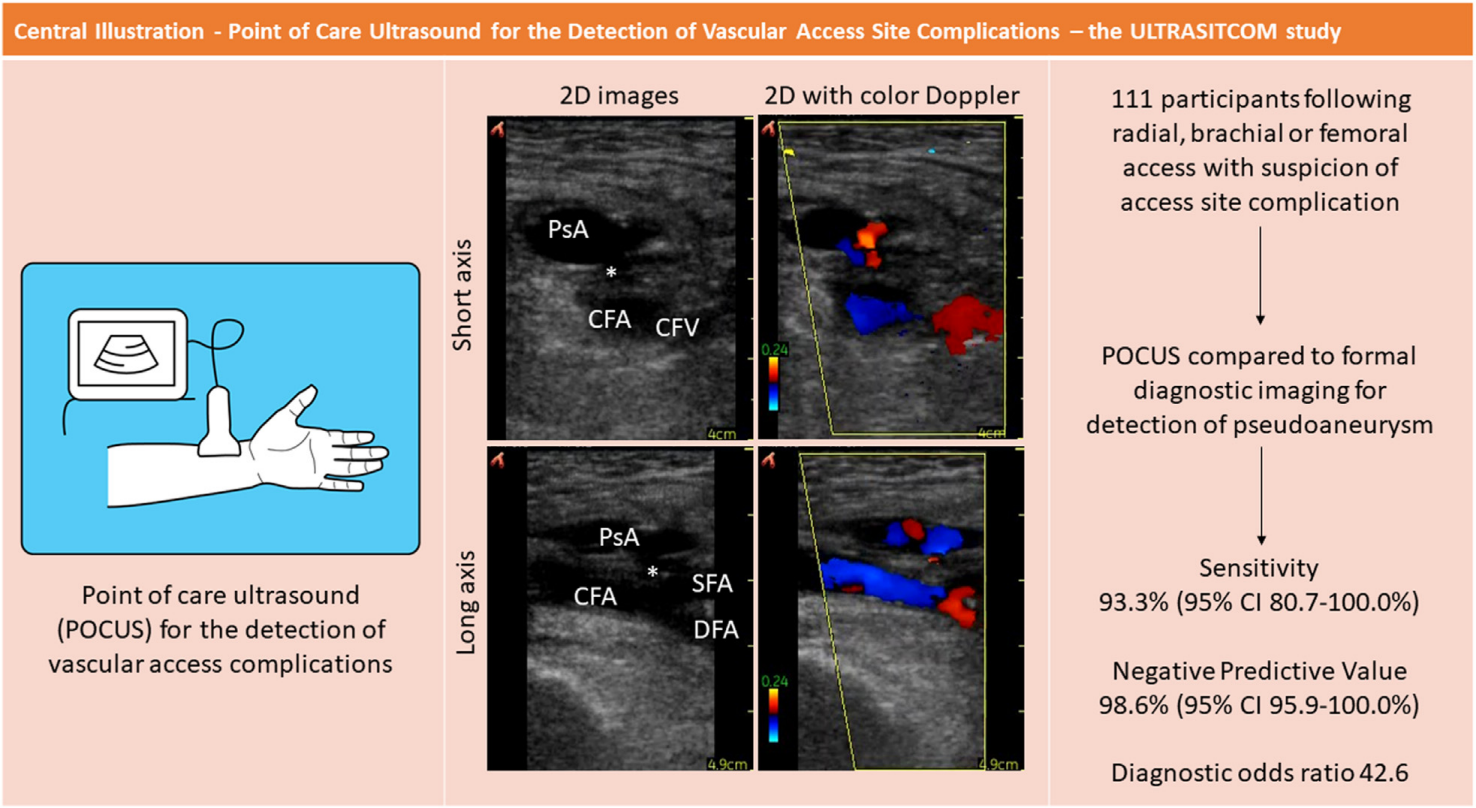
**Performance characteristics of the comprehensive physical examination pluspoint-of-careultrasound (POCUS) assessment in the diagnosis of postprocedural pseudoaneurysm (PsA).** In a cohort of 111 patients following radial, brachial, or femoral access, combined physical examination and POCUS assessment had greater sensitivity (93.3%) and negative predictive value (98.6%) in the diagnosis of PsA compared to physical examination alone; POCUS skills were rapidly acquired after a single 2-hour training session with a vascular radiologist. CFA, common femoral artery; DFA, deep femoral artery; SFA, superficial femoral artery.

**Table 2:** Diagnostic accuracy of clinical and POCUS assessments for pseudoaneurysm detection.

	Physical examination + POCUS	Physical examination only
Diagnostic odds ratio^a^	42.6 (95% CI, 34.6-50.6)	15.6 (95% CI, 11.7-19.5)
Sensitivity	93.3% (95% CI, 80.7%-100.0%)	80.0% (95% CI, 59.8%-100.0%)
Negative predictive value	98.6% (95% CI, 95.9%-100.0%)	96.1% (95% CI, 91.8%-100.0%)
Specificity	75.3% (95% CI, 66.5%-84.0%)	79.6% (95% CI, 71.4%-87.8%)
Positive predictive value	37.8% (95% CI, 22.2%-53.5%)	38.7% (95% CI, 21.6%-55.9%)
Overall accuracy	77.8% (95% CI, 68.8%-85.2%)	79.6% (95% CI, 70.8%-86.8%)

POCUS, point-of-care ultrasound.

a *P* value comparing diagnostic odds ratio between combined physical examination + POCUS vs physical examination only is <.01.

**Figure 2:**
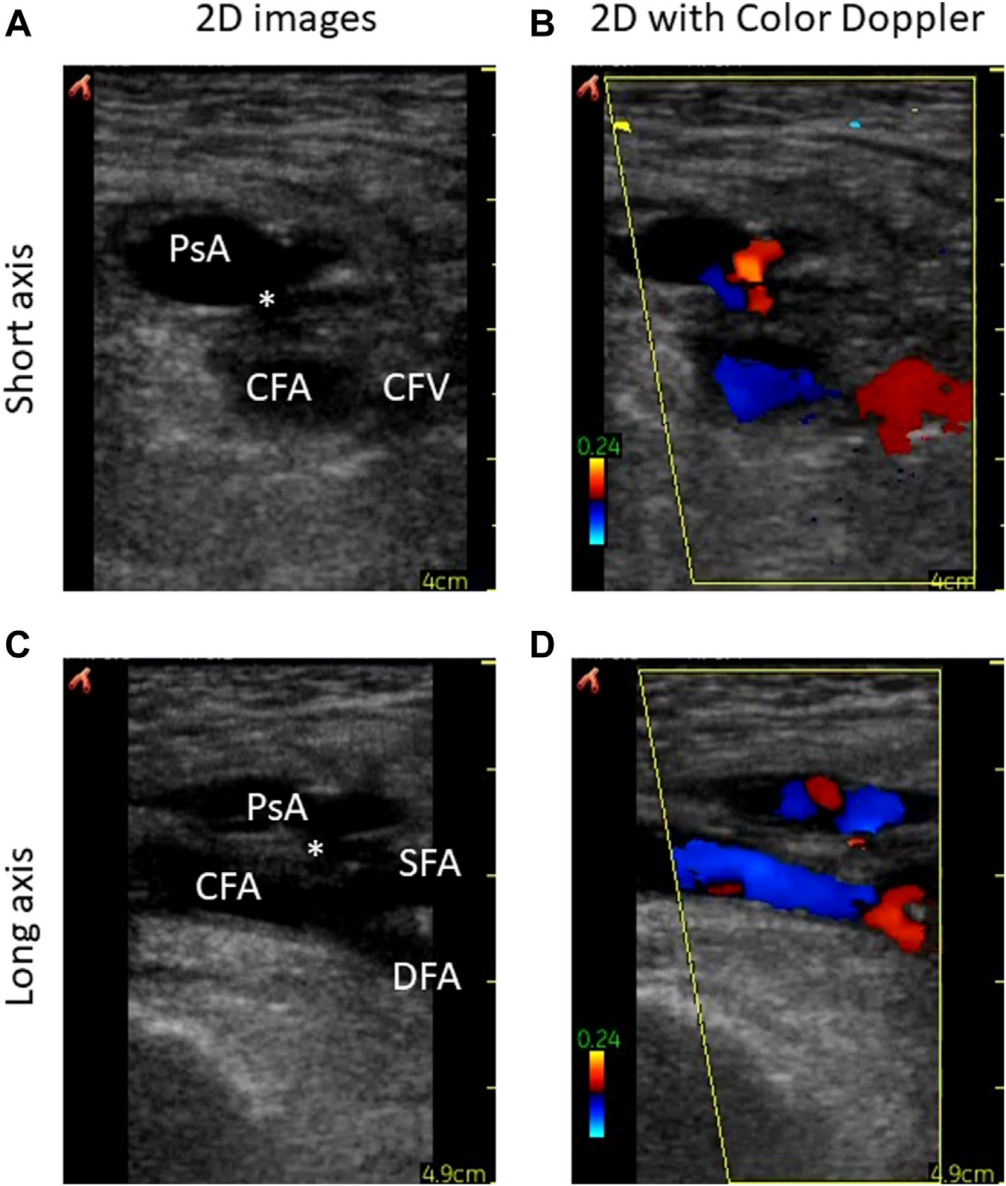
**Pseudoaneurysm (PsA) identified on point-of-care ultrasound.** PsA as visualized on point-of-care ultrasound with the neck of the PsA (∗) just proximal to the femoral artery bifurcation in the (**A**) short axis without color, (**B**) short axis with color Doppler, (**C**) long axis without color, and (**D**) long axis with color Doppler. CFA, common femoral artery; DFA, deep femoral artery; SFA, superficial femoral artery.

Using the reference standard, a pseudoaneurysm was detected in 15 study participants (13.9%)—9 of which occurred following transfemoral access and 6 occurred after transradial access (Figure 2). There were no brachial artery pseudoaneurysms identified. Thirteen of the pseudoaneurysms occurred in patients in whom the maximal sheath size was 6F, and 2 of the pseudoaneurysms occurred in patients with 9F sheaths. The DOR for the detection of pseudoaneurysm was greater with the combined clinical and POCUS assessment compared to the physical examination only (42.6 [95% CI, 34.6-50.6] vs 15.6 [95% CI, 11.7-19.5]; *P* < .01). The sensitivity for pseudoaneurysm detection was greater using the combined physical examination and POCUS assessment, compared to physical examination alone (93.3% [95% CI, 80.7%-100.0%] vs 80.0% [95% CI, 59.8%-100.0%]) The negative predictive value for pseudoaneurysm was similar between the 2 assessment methods (combined: 98.6% [95% CI, 95.9%-100.0%] vs physical examination only: 96.1% [95% CI, 91.8%-100.0%]).

### Management of access site complications

Most study participants with pseudoaneurysm required either a thrombin injection (20.0%) or underwent surgical arterial repair (40.0%) (Table 3). The remaining study participants with pseudoaneurysm were treated conservatively. Of note, the single participant in whom a pseudoaneurysm was missed by combined clinical and POCUS assessments did not require arterial repair or thrombin injection.

**Table 3:** Management of access site complications.

	Pseudoaneurysm (n = 15)
Conservative/manual compression	6 (40.0%)
Thrombin injection	3 (20.0%)
Surgical repair	6 (40.0%)

## Discussion

The ULTRASITCOM study evaluated the utility of POCUS as an adjunct to clinical assessment in detecting vascular access site complications, namely pseudoaneurysms, following invasive cardiac procedures. The study has 3 major findings. First, the diagnostic performance as reflected in a DOR of 42.6 for POCUS in this postprocedure population suggests a strong overall performance. Second, the sensitivity of a combined clinical and POCUS evaluation was high at 93.3% with a negative predictive value of 98.6%—suggesting that a negative study was reassuring to rule out pseudoaneurysm. Third, the cardiology fellows had a 2 hour of didactic training with no further hands-on training suggesting a rapid acquisition of the skill set necessary to perform these assessments. Thus, although the performance of POCUS in this study is excellent, one would expect that with more experience and training, interventional cardiologists and fellows could attain diagnostic accuracy approaching that of formal studies.

Although many types of vascular access complications are possible, including hematoma, AV fistula, active extravasation/bleeding, and venous and/or arterial thrombosis, the focus of the ULTRASITCOM study was pseudoaneurysm. A pseudoaneurysm is a contained rupture of an artery that involves disruption in all 3 layers of the arterial wall and is usually a consequence of an arterial puncture that does not adequately seal.^7^ This allows pulsatile blood to track into the perivascular space and a hematoma with surrounding tissue forms the wall of the pseudoaneurysm. The incidence of this complication is reported to be 2% to 6% following percutaneous coronary intervention.^7^ Although some pseudoaneurysms may thrombose spontaneously, this complication could be life-threatening should the pseudoaneurysm rupture. Nonetheless, there are many pseudoaneurysms that are small and managed conservatively. In the current study, the only pseudoaneurysm that was not identified on POCUS was not clinically relevant and was managed conservatively. Thus, we are reassured that all clinically relevant pseudoaneurysms were identified with the combined clinical and POCUS assessment strategy.

The result of the ULTRASITCOM study has an important implication for clinical practice and training. Increasingly, POCUS for cardiac applications is being integrated into cardiology training and interventional fellows increasingly have vascular ultrasound skills for access.^11^ Although commonly used to evaluate cardiac pathology, this is—to the best of our knowledge—the first application for assessment of vascular access complications. Indeed, with relatively abbreviated training, learners were able to perform and diagnose pseudoaneurysms with a high degree of accuracy. Furthermore, there have been individual reports in which the addition of POCUS has resulted in point-of-care diagnosis.^12^^,^^13^ As a result, one can envision POCUS for vascular complications becoming a standard part of interventional training to enable timely interventions, and potentially reduce morbidity and health care costs.

This study is not without limitations. First, a nonrandomized, convenience sample size from a single center was used, which may limit the external generalizability of the study results. All efforts were made to include patients in whom there was concern regarding an access site complication over the 18-month study period. Second, a variety of invasive procedures were performed, and some procedural factors are associated with an increased risk of pseudoaneurysm (eg, sheath size, anticoagulation, simultaneous arterial and venous cannulation).^7^ However, although various procedures were performed, we do not believe this impacted the ability of the POCUS scans to detect the presence of a pseudoaneurysm. Third, the general and interventional cardiology fellows underwent a formal but unvalidated 2-hour training session with a radiologist and the impact this had on the ability to detect pseudoaneurysms remains unknown. Whether more optimized training is needed remains to be studied. Fourth, POCUS and physical examination evaluations were compared to 2 different reference modalities to confirm the diagnosis of a pseudoaneurysm. However, both Doppler ultrasonography and CT angiography have demonstrated similar sensitivity and specificity in the detection of pseudoaneurysms. Additionally, although the absolute number of large-bore access sheaths was small, the majority of pseudoaneurysms occurred following procedures requiring 6F access sheaths. Lastly, the direct and indirect health care costs and possible adverse events associated with delays in obtaining formal imaging are not available.

## Conclusion

Point-of-care ultrasound when combined as an adjunct to clinical assessment, is a highly sensitive tool for detecting pseudoaneurysms following invasive cardiovascular procedures. These findings suggest the potential integration of POCUS into routine practice, which could result in timely complication identification and management, thereby improving patient outcomes and reducing health care costs. Further studies are needed to confirm whether POCUS identification of pseudoaneurysms can translate into improved clinical outcomes.
