# Supplementary material for: Point-of-Care Ultrasound for the Detection of Vascular Access Site Complications—The ULTRASITCOM Study
Source: J Soc Cardiovasc Angiogr Interv. 2025 Feb 18;4(2):102516. doi: 10.1016/j.jscai.2024.102516 (PMC11916795; doi:10.1016/j.jscai.2024.102516)
Supplement: Supplemental Table S1 and S2 [file mmc1.docx]

Supplemental Table S1 – Combined physical examination and point of care ultrasound assessment for the detection of pseudoaneurysm

|  | **Reference test positive**  (i.e. pseudoaneurysm present) | **Reference test negative**  (i.e. pseudoaneurysm absent) |  |
| --- | --- | --- | --- |
| **Combined assessment positive**  (i.e. pseudoaneurysm suspected) | 14 | 23 | **Positive predictive value**  37.8% (95% CI 22.2-53.5%) |
| **Combined assessment negative**  (i.e. pseudoaneurysm not suspected) | 1 | 70 | **Negative predictive value**  98.6% (95% CI 95.9-100.0%) |
|  | **Sensitivity**  93.3% (95% CI 80.7-100.0%) | **Specificity**  75.3% (95% CI 66.5-84.0%) | **Overall diagnostic accuracy**  77.8% (95% CI 68.8-85.2%) |

Supplemental Table S2 - Physical examination only for detection of pseudoaneurysm (n=108)

|  | **Reference test positive**  (i.e. pseudoaneurysm present) | **Reference test negative**  (i.e. pseudoaneurysm absent) |  |
| --- | --- | --- | --- |
| **Clinical exam positive**  (i.e. pseudoaneurysm suspected) | 12 | 19 | **Positive predictive value**  38.7% (95% CI 21.6-55.9%) |
| **Clinical exam negative**  (i.e. pseudoaneurysm not suspected) | 3 | 74 | **Negative predictive value**  96.1% (95% CI 91.8-100.0%) |
|  | **Sensitivity**  80.0% (95% CI 59.8-100.0%) | **Specificity**  79.6% (95% CI 71.4-87.8%) | **Overall diagnostic accuracy**  79.6% (95% CI 70.8-86.8%) |
