# Supplementary material for: Point-of-Care Ultrasound for the Detection of Vascular Access Site Complications—The ULTRASITCOM Study
Source: J Soc Cardiovasc Angiogr Interv. 2025 Feb 18;4(2):102516. doi: 10.1016/j.jscai.2024.102516 (PMC11916795; doi:10.1016/j.jscai.2024.102516)
Supplement: Supplementary Material [file mmc2.pdf]

Ultrasound

Terminology, Technique & Basic Principles

US Transducer Types

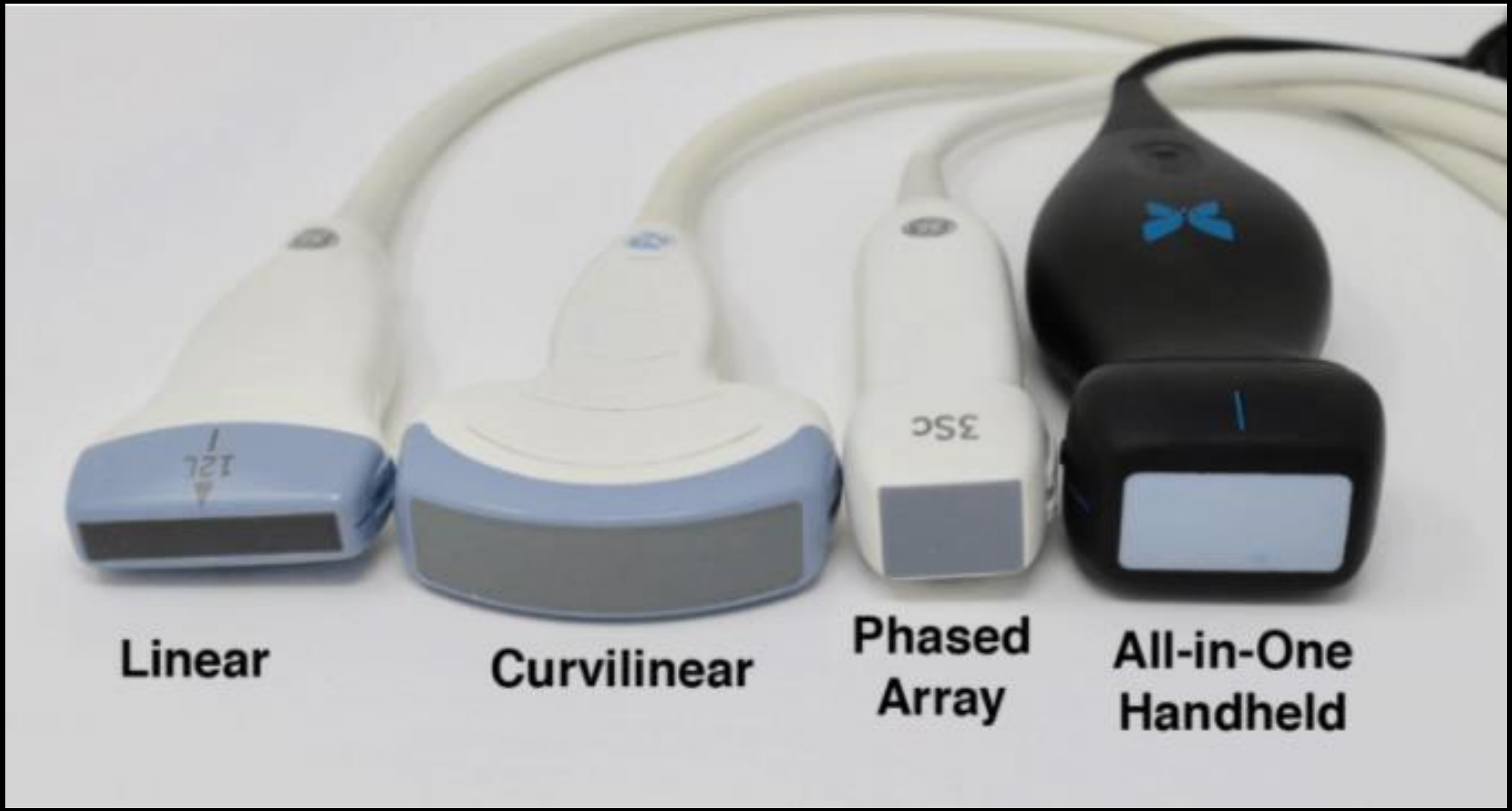

Linear Probe
High Frequency
Linear Footprint
Shallow Structures (< 8cm)

Vessels

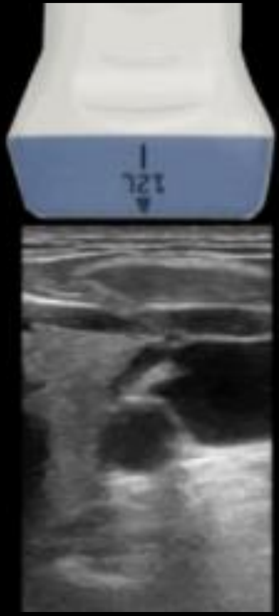

Curvilinear Probe
Low Frequency
Wide Footprint
Deep Structures

Deep Organs

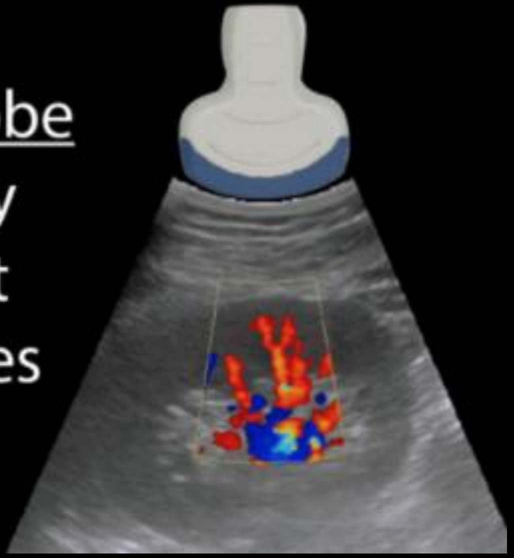

Phased Array Probe
Low Frequency
Narrow Footprint
Deep Structures

Heart

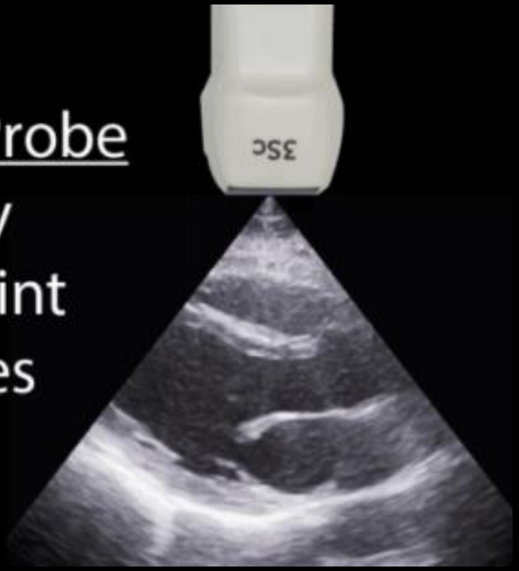

Ultrasound Vocabulary

- Anechoic 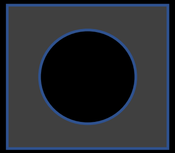
- Hypoechoic 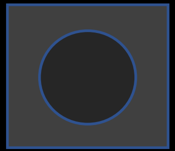
- Isoechoic 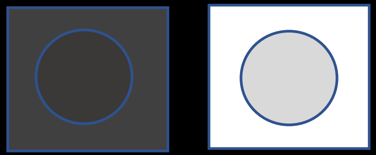
- Hyperechoic 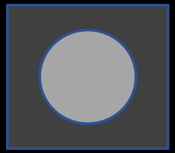
- Complex fluid 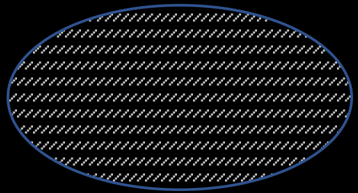

Echogenicity

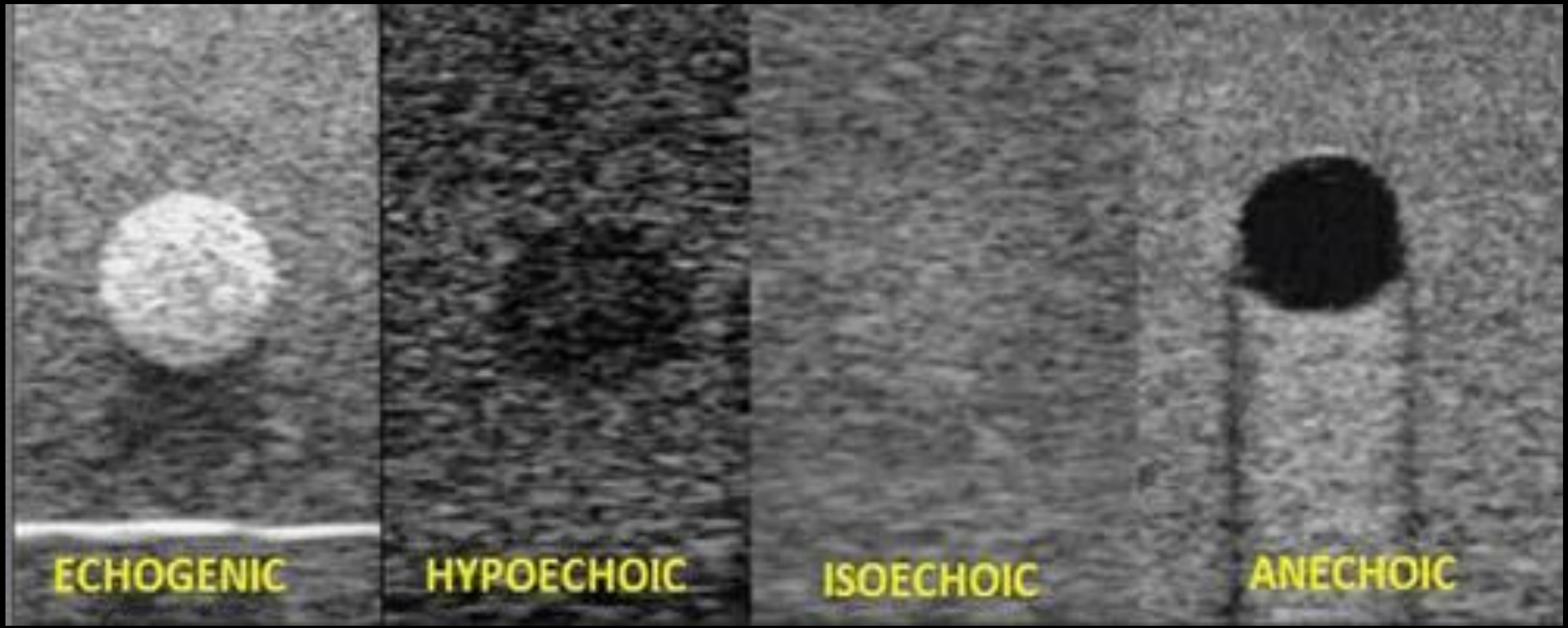

(Hyperechoic)

Complex fluid (mixed echogenicity)

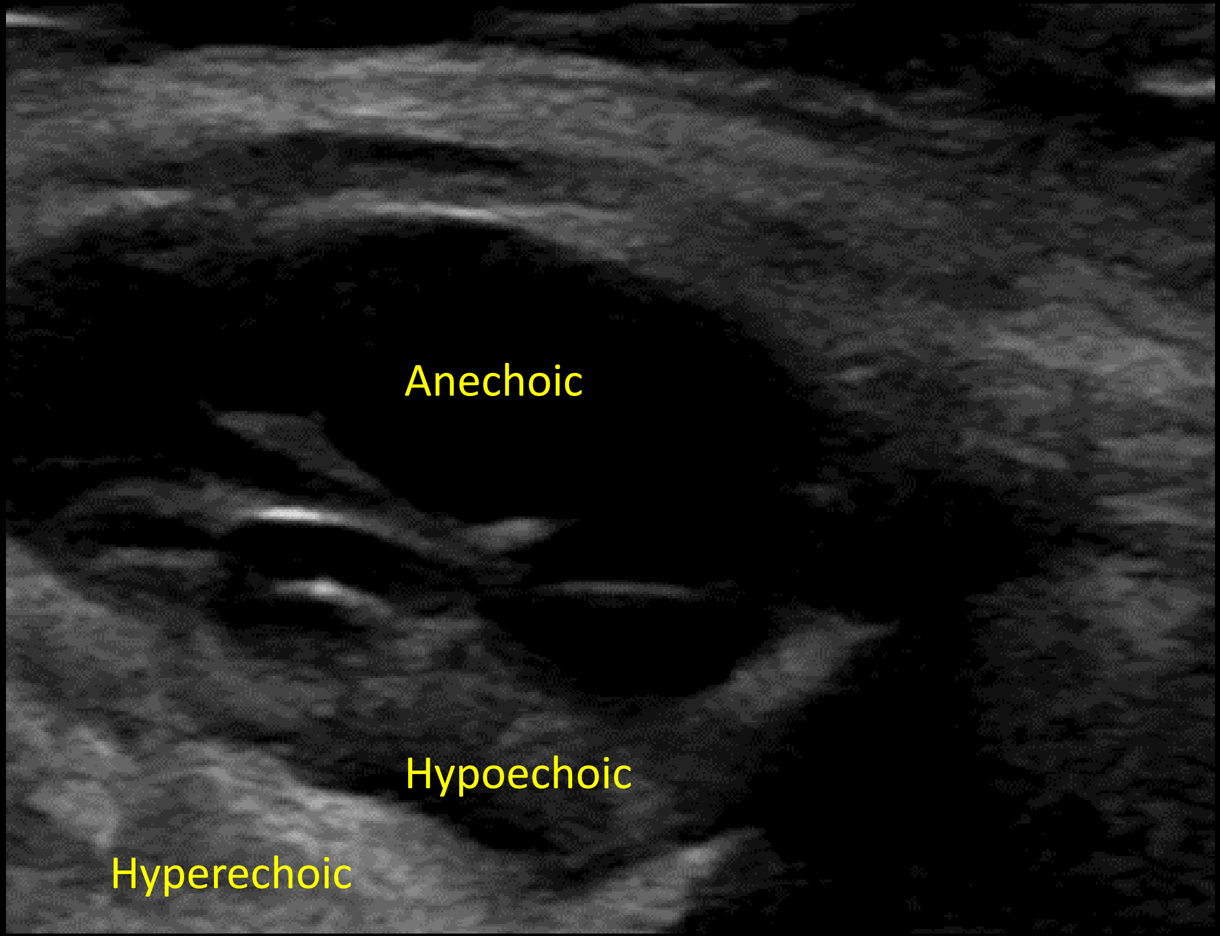

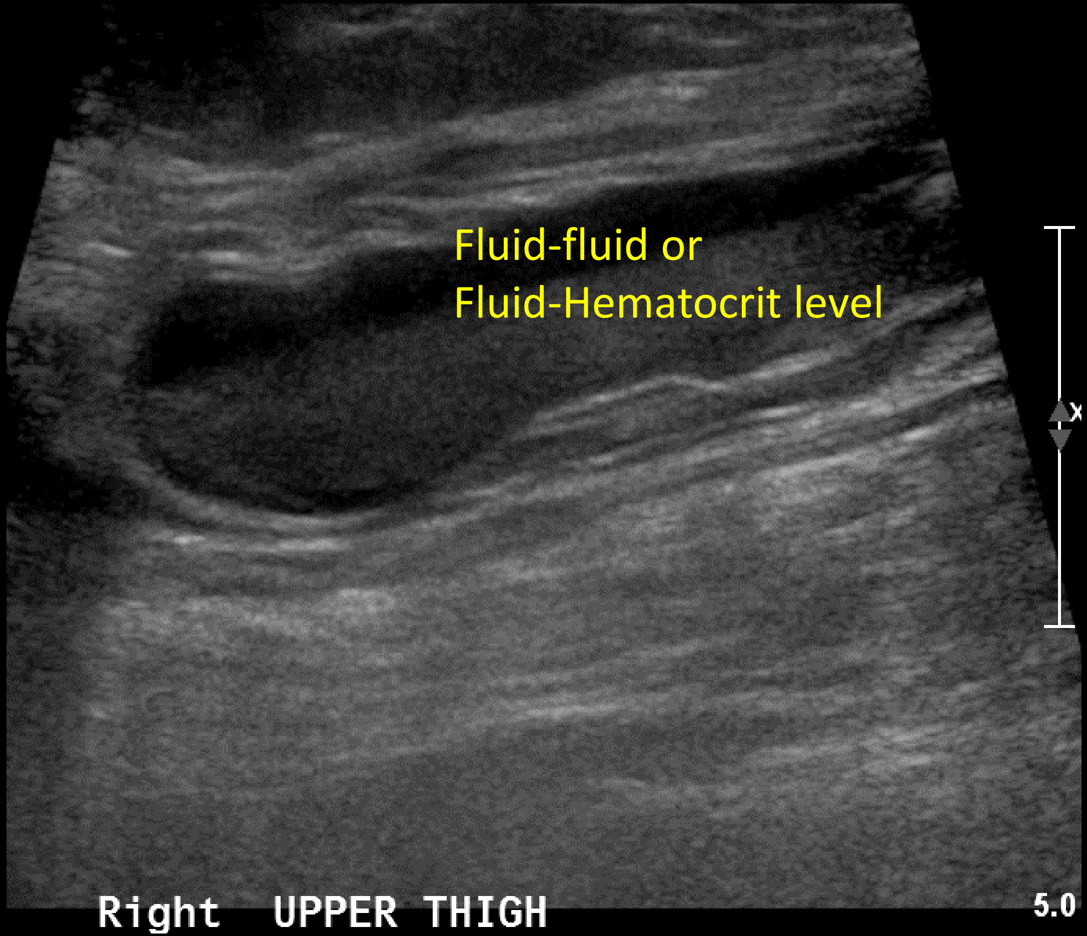

Ultrasound Vocabulary

- Colour Doppler
- Power Doppler
- Spectral Doppler

- Ying-Yang
- Aliasing
- Laminar flow
- To-and-Fro flow

Colour Doppler

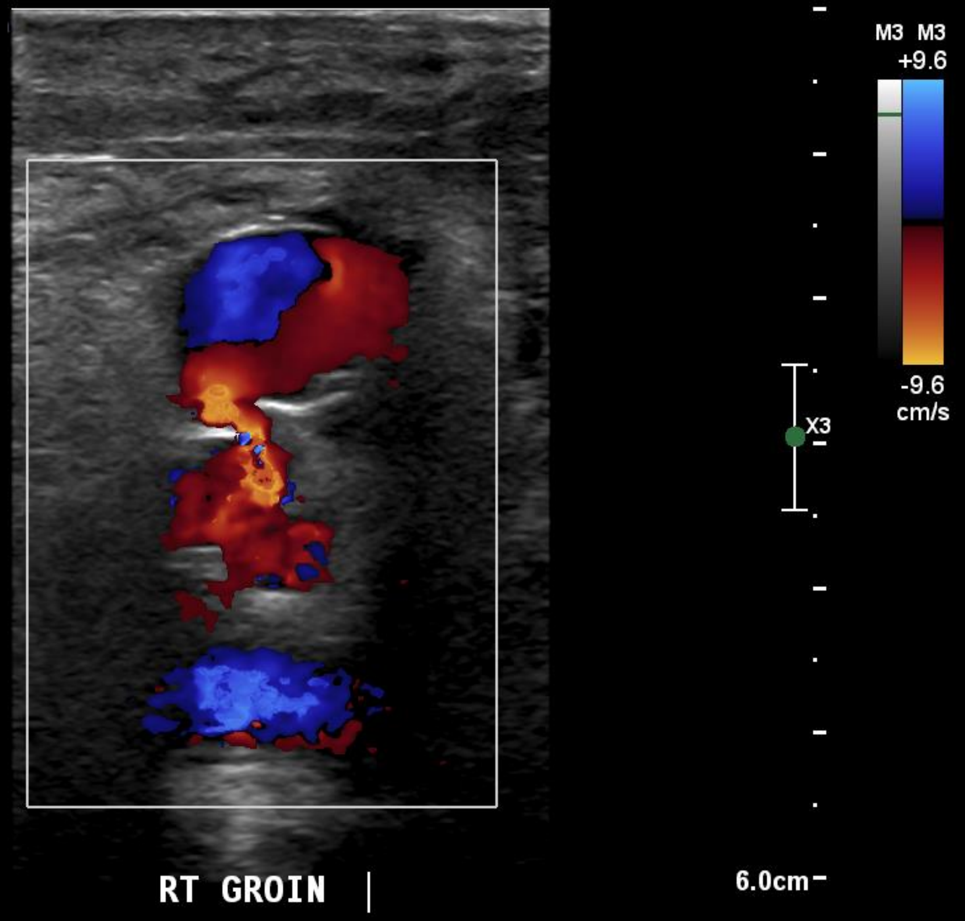

- Applying 'colour' to determine:
  - Is there flow?
  - Direction of flow?
  - Direction is based on scale and if flow is towards or away from the transducer

Ying-Yang

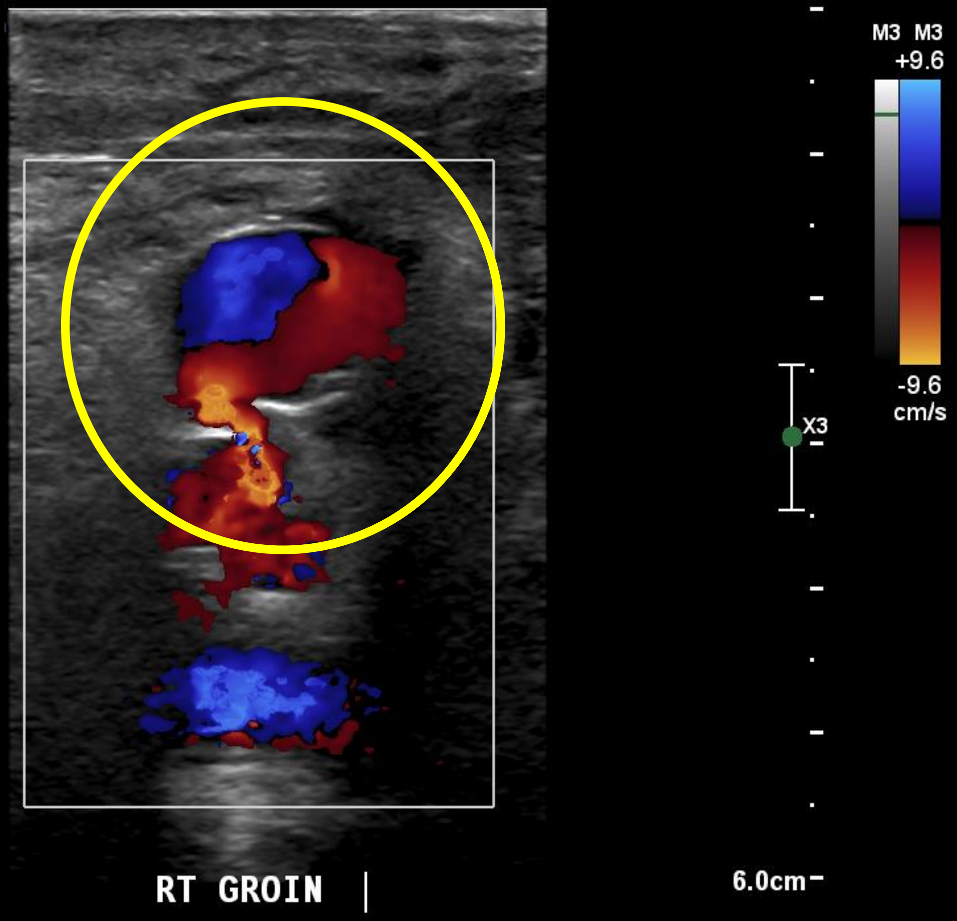

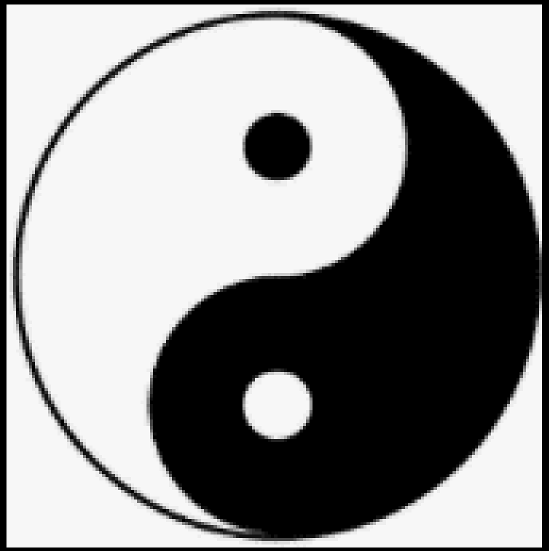

Power Doppler

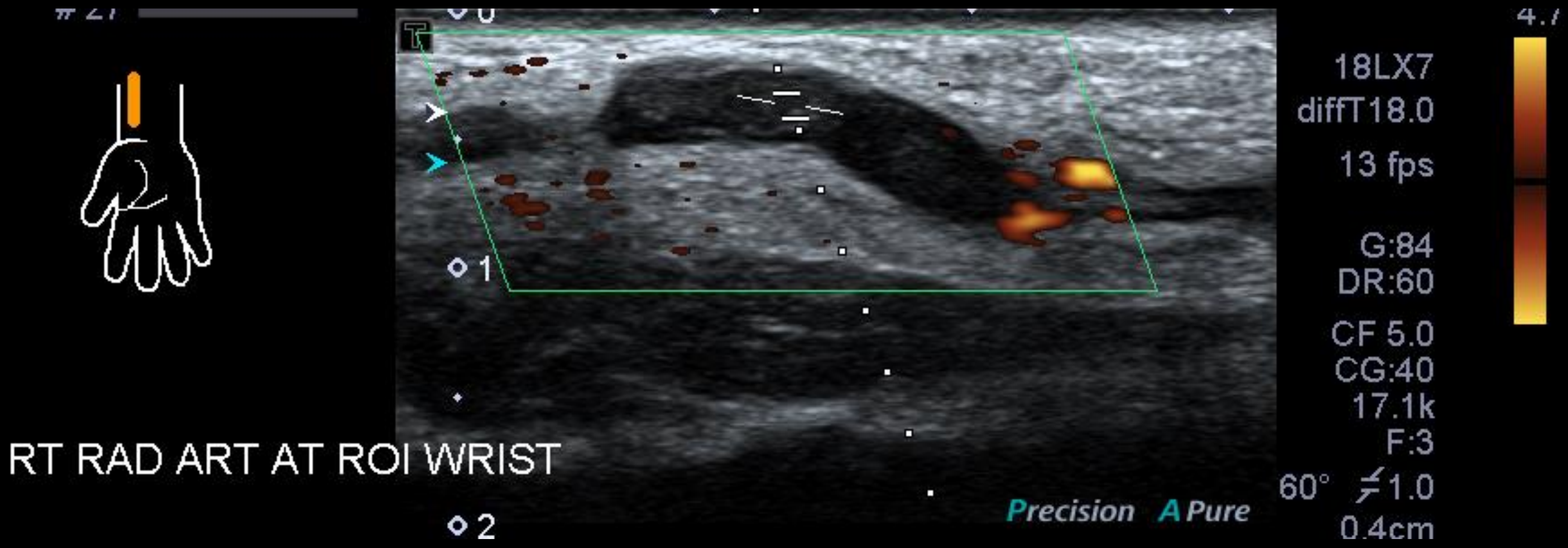

- Detects subtle/slow flow
- But does not indicate direction
- Comes up as “orange/yellow” colour setting

Colour vs Power Doppler

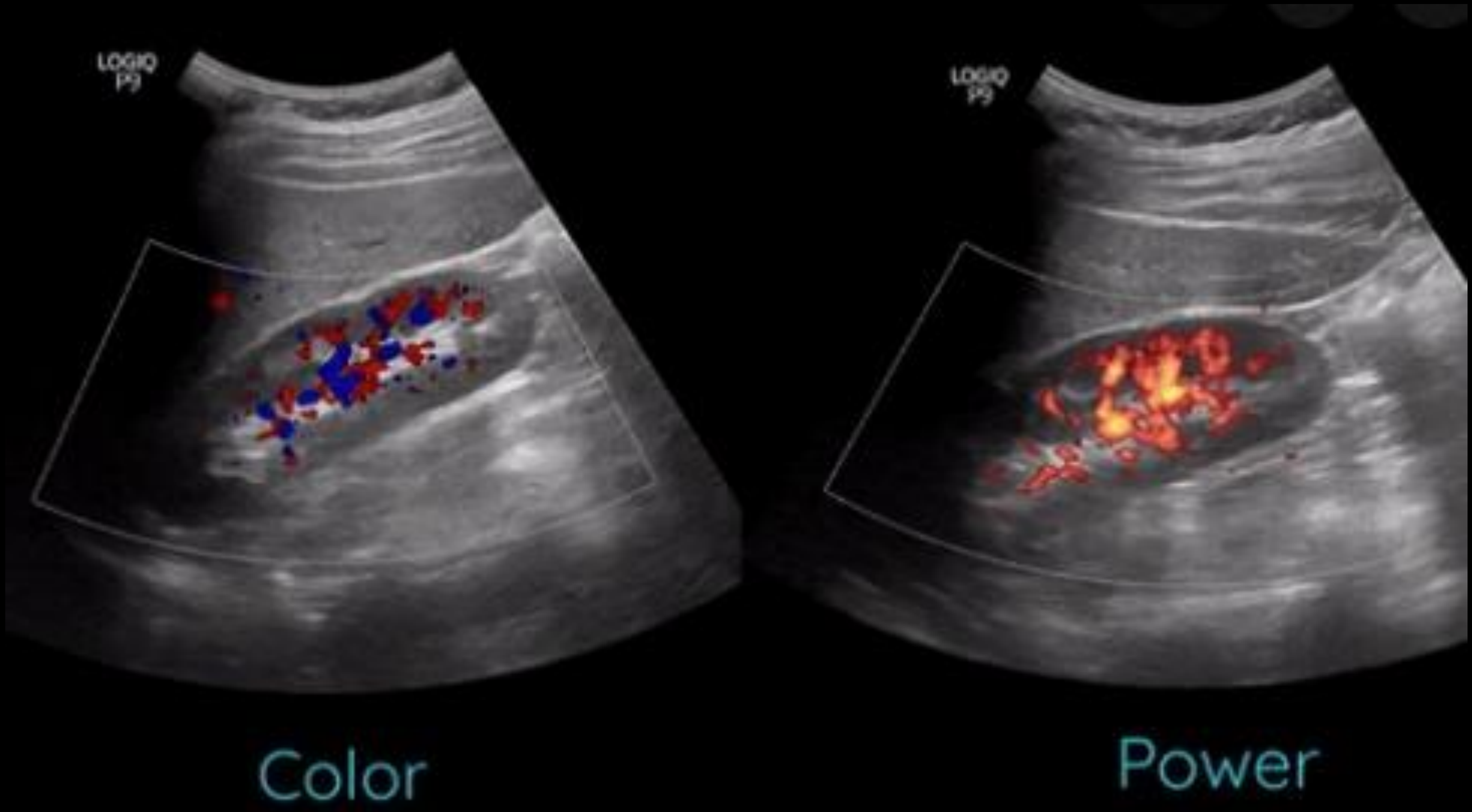

Spectral Doppler

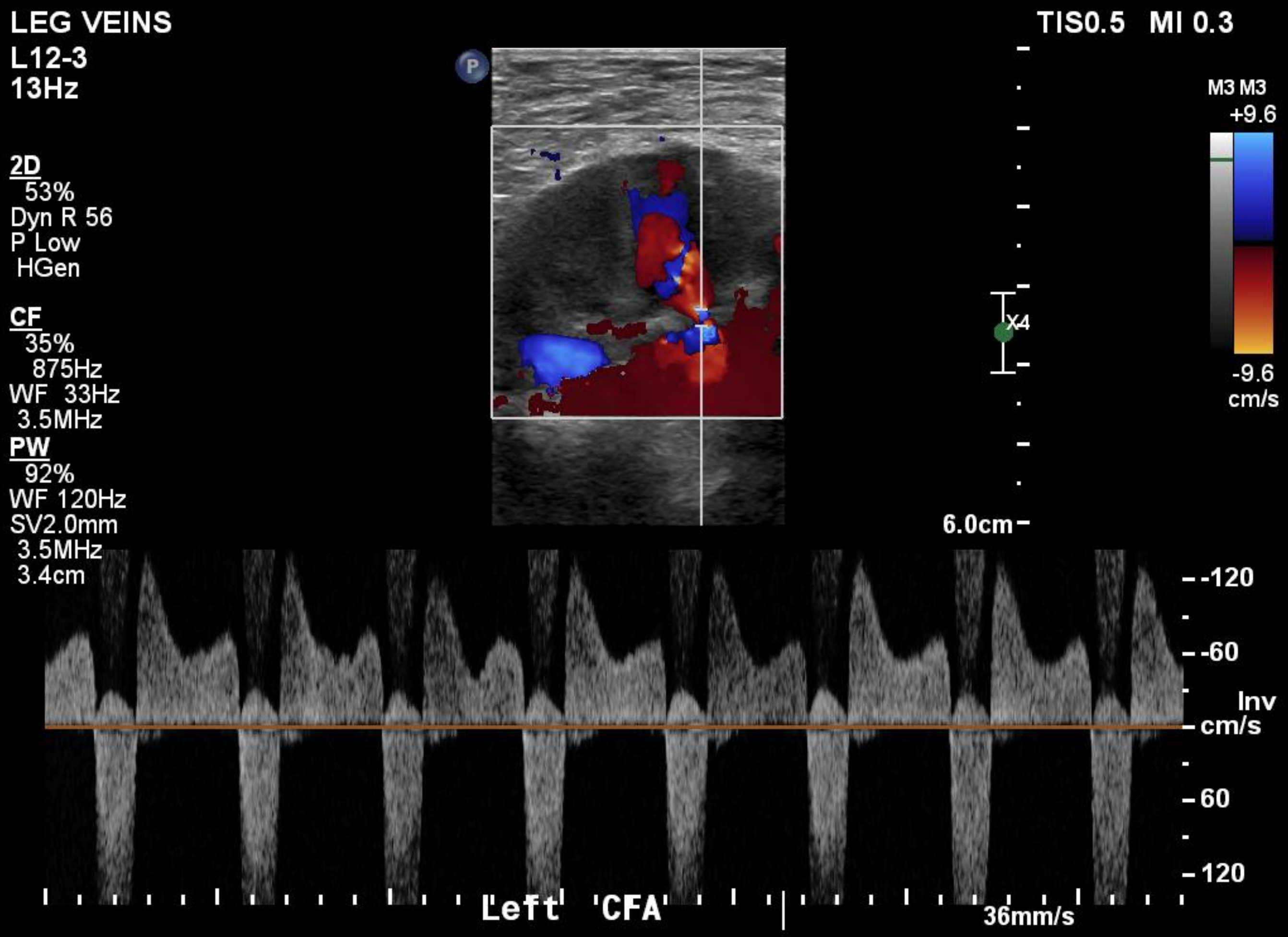

Above baseline = toward transducer

Below baseline = away from transducer

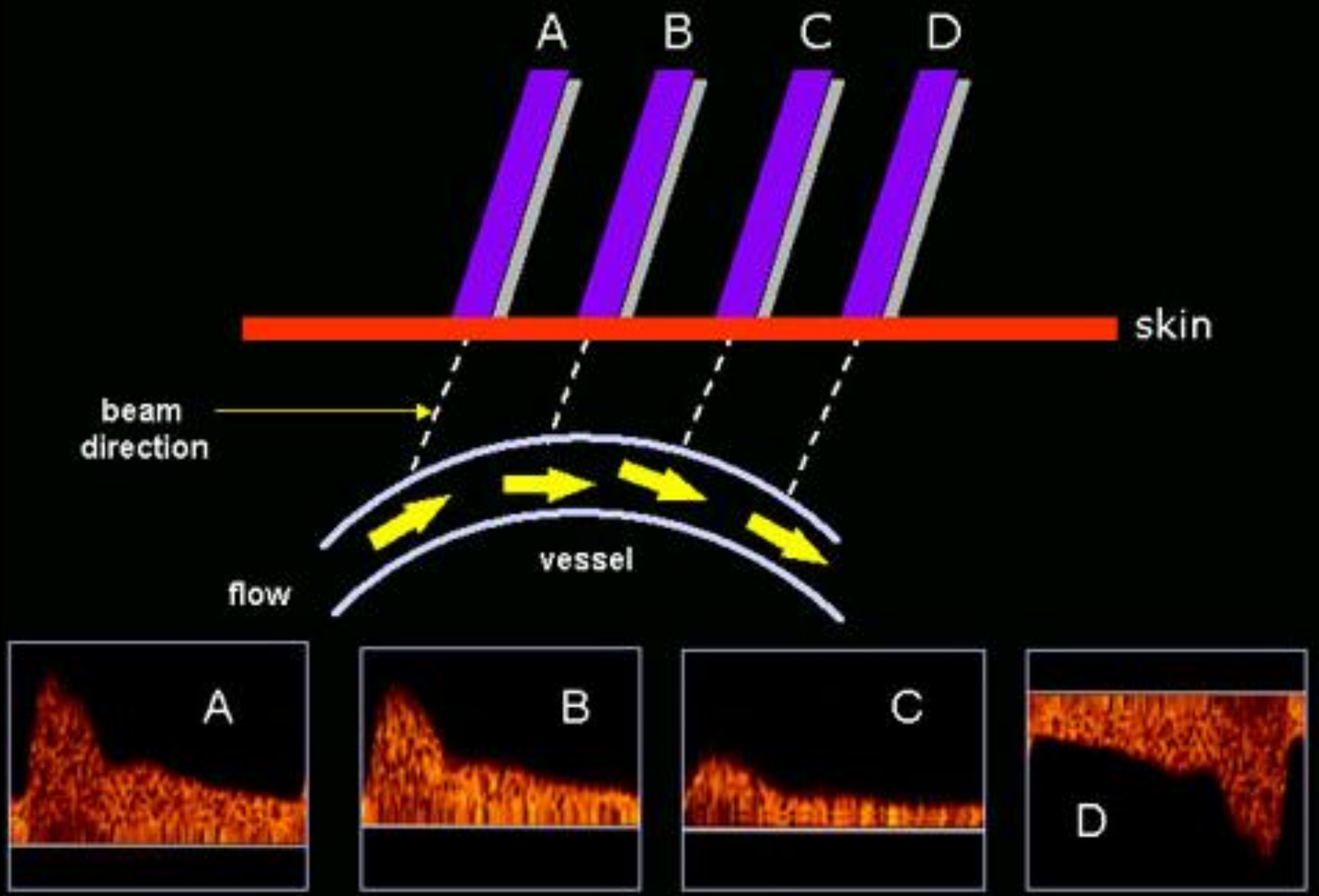
A
B
C
D
skin
beam direction
vessel
flow
A
B
C
D

Steering/Angle

- Transducer must be positioned at 60 degrees or less with respect to vessel being sampled

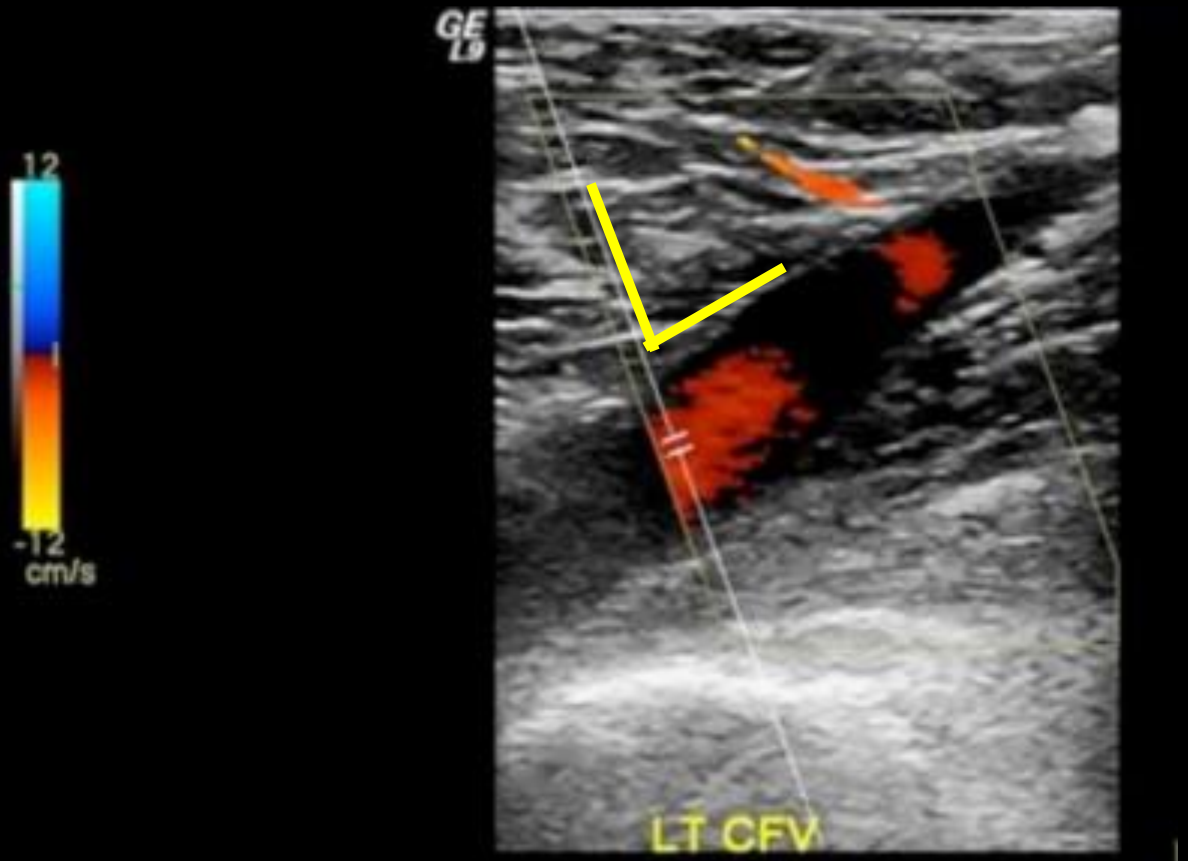

90 degrees = bad

VS

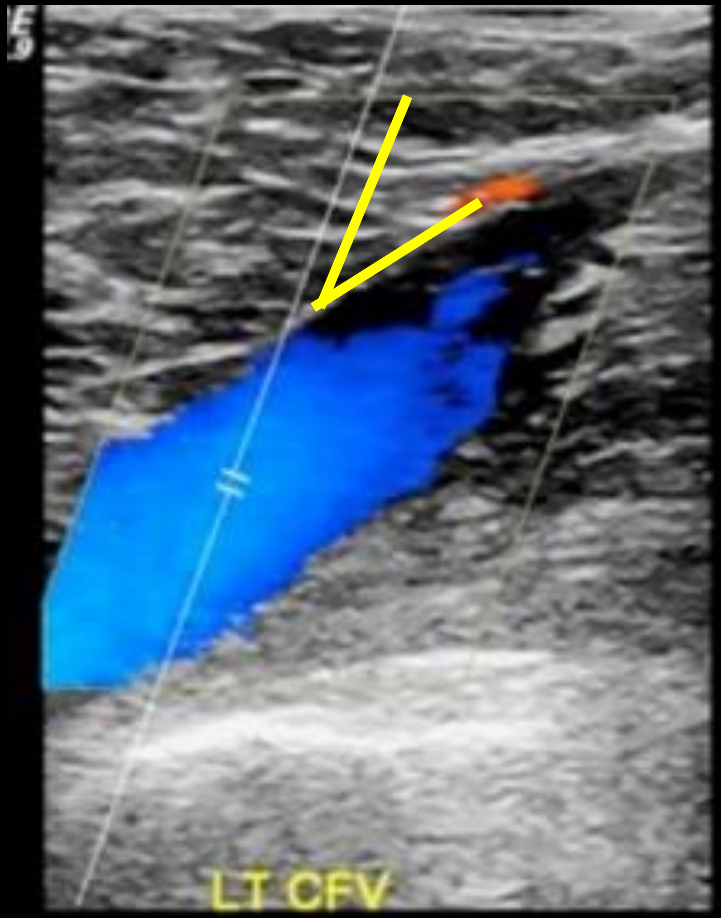

Want 60 degrees or less

Over and Undergaining Colour

- Adjust gain appropriately so that vessels on colour are not “bleeding out” or “undercoloured”

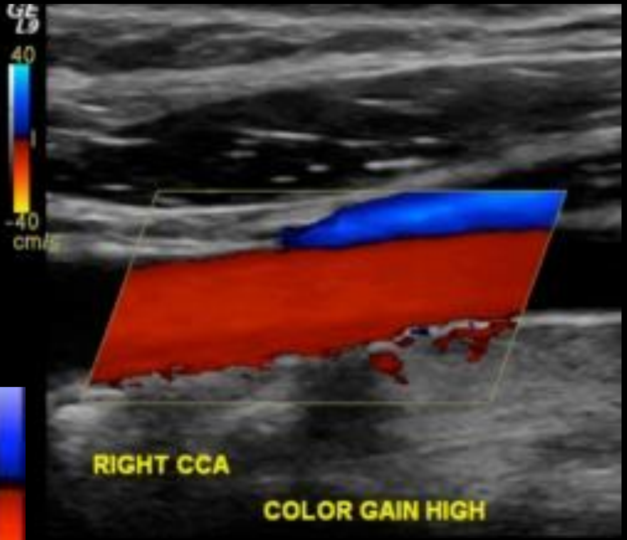

YES!
(Just right)

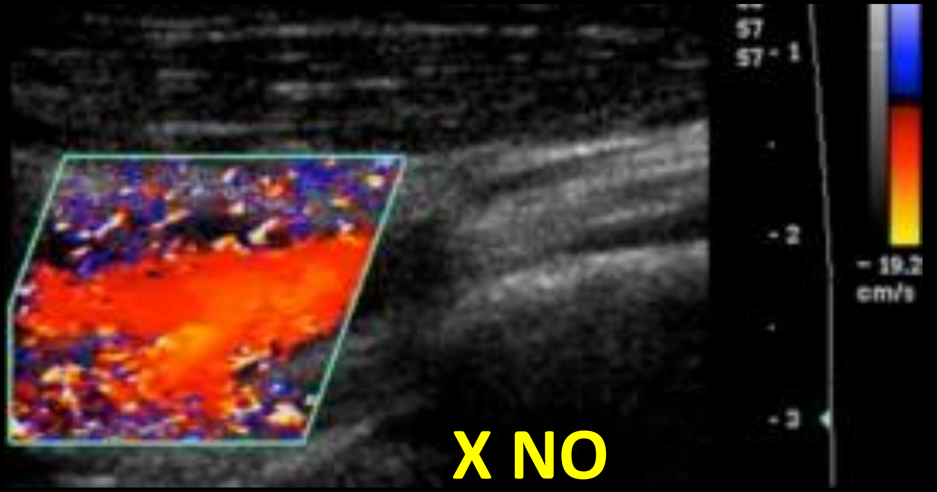

X NO
(Over)

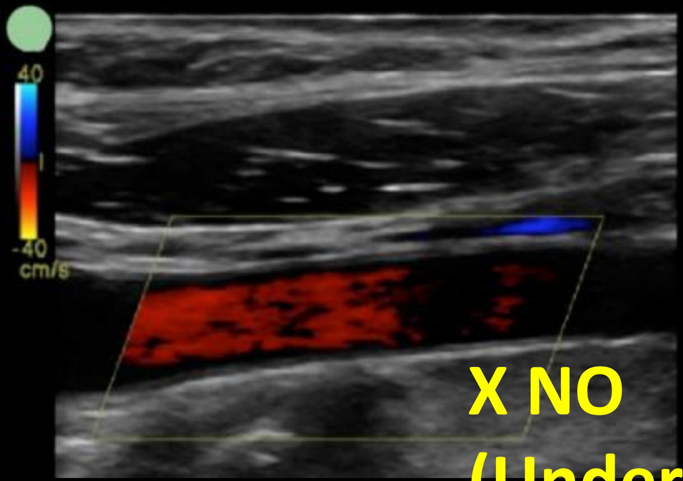

X NO
(Under)

Aliasing = turbulent flow

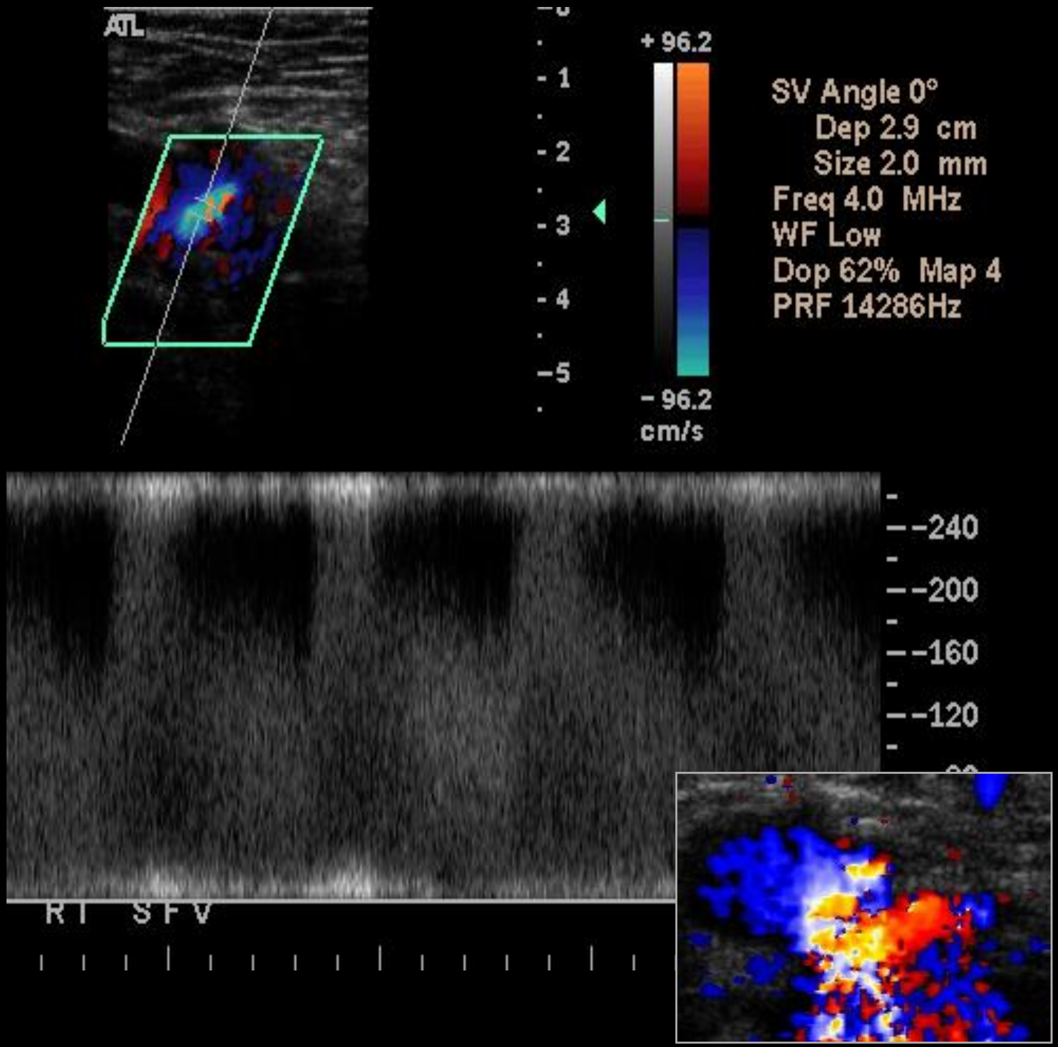

# vs Laminar flow

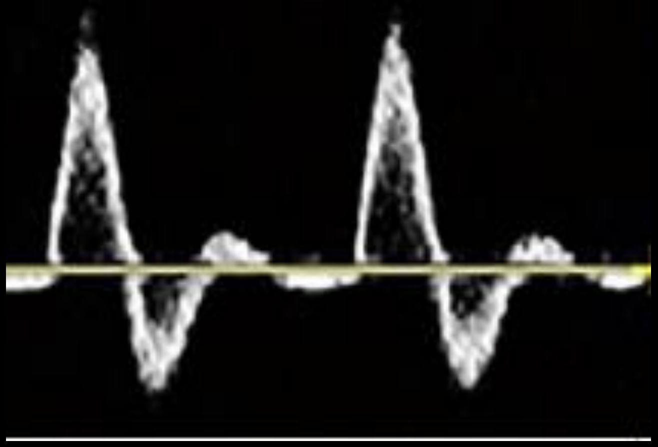

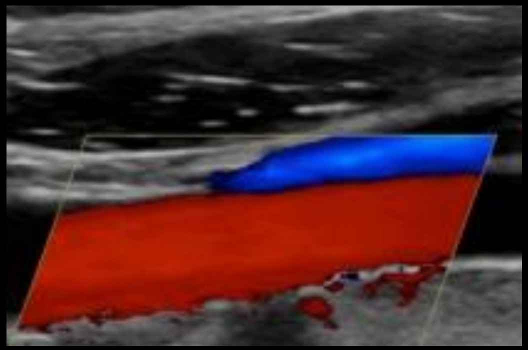

To-and-fro flow (aka bidirectional flow)

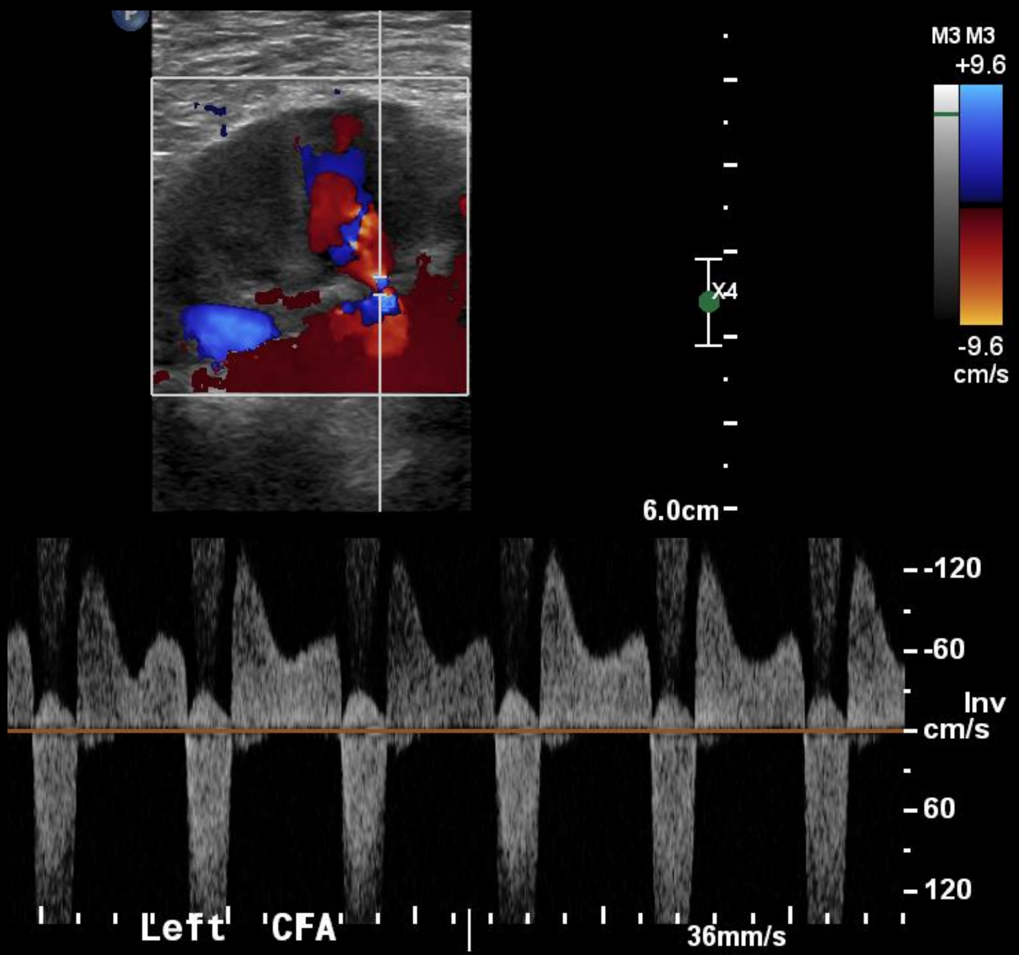

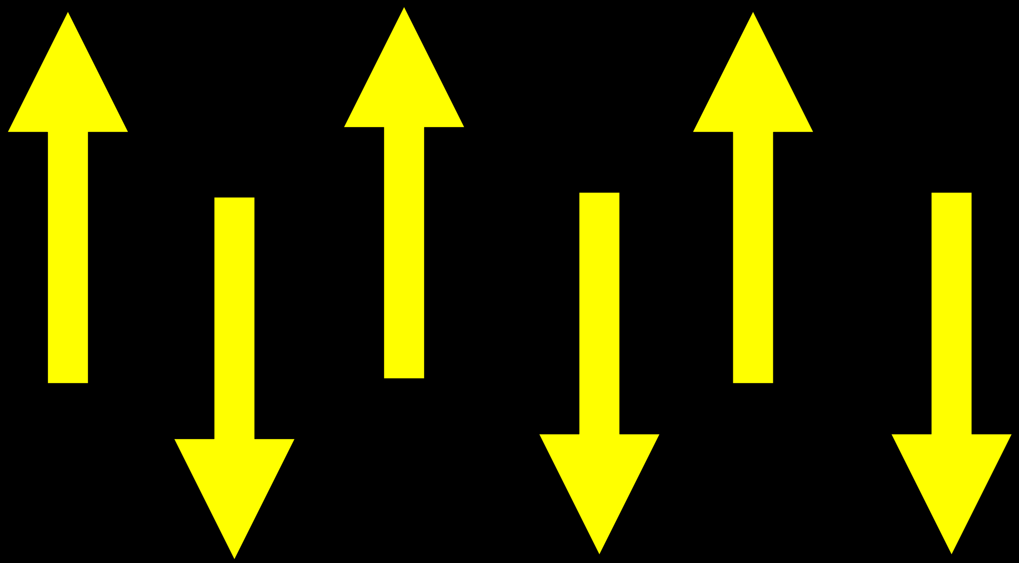

Anatomy
Groin and Wrist

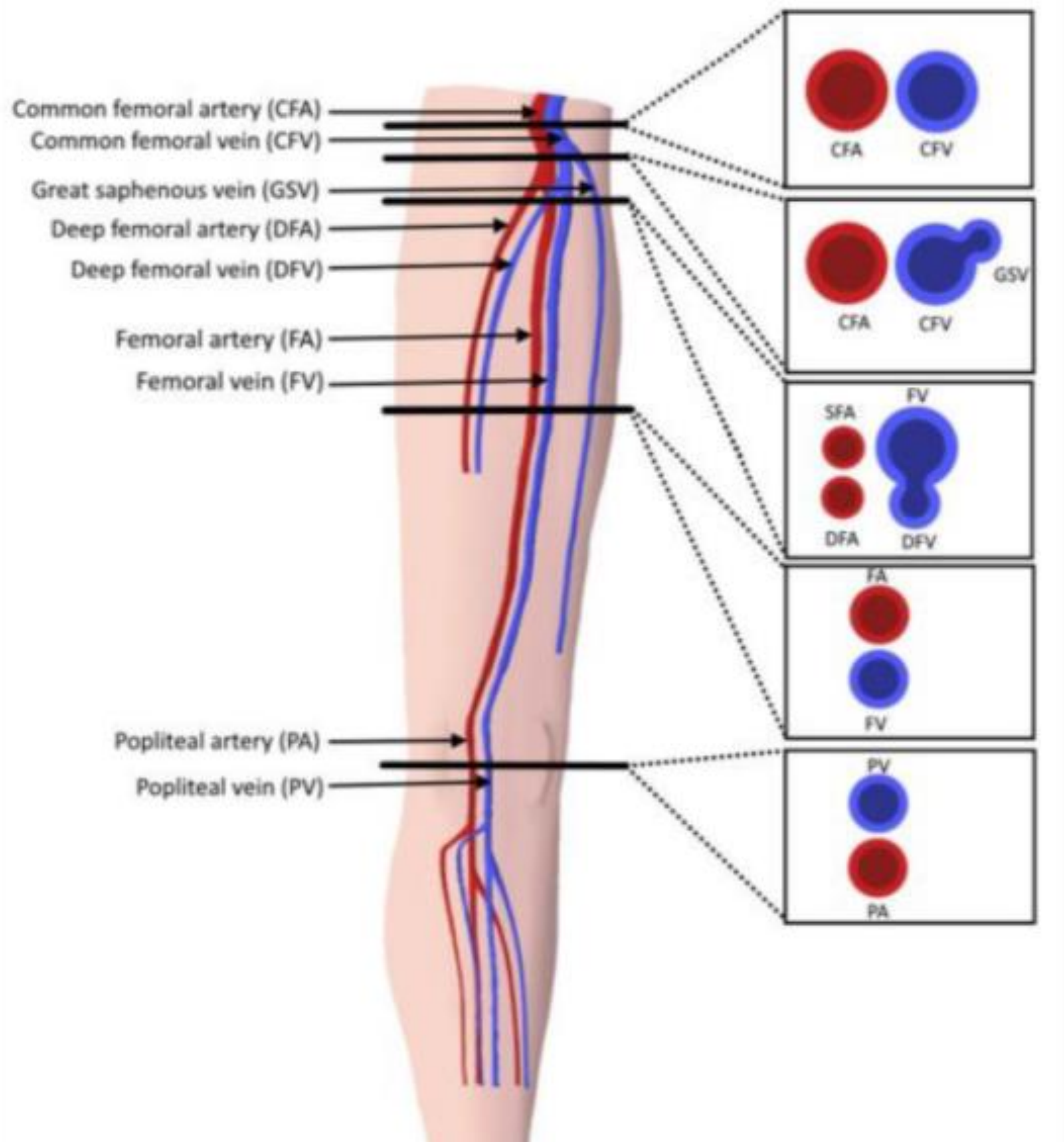
Common femoral artery (CFA)
Common femoral vein (CFV)
Great saphenous vein (GSV)
Deep femoral artery (DFA)
Deep femoral vein (DFV)
Femoral artery (FA)
Femoral vein (FV)
Popliteal artery (PA)
Popliteal vein (PV)
CFA
CFV
CFA
CFV
GSV
SFA
FV
DFA
DFV
FA
FV
PV
PA

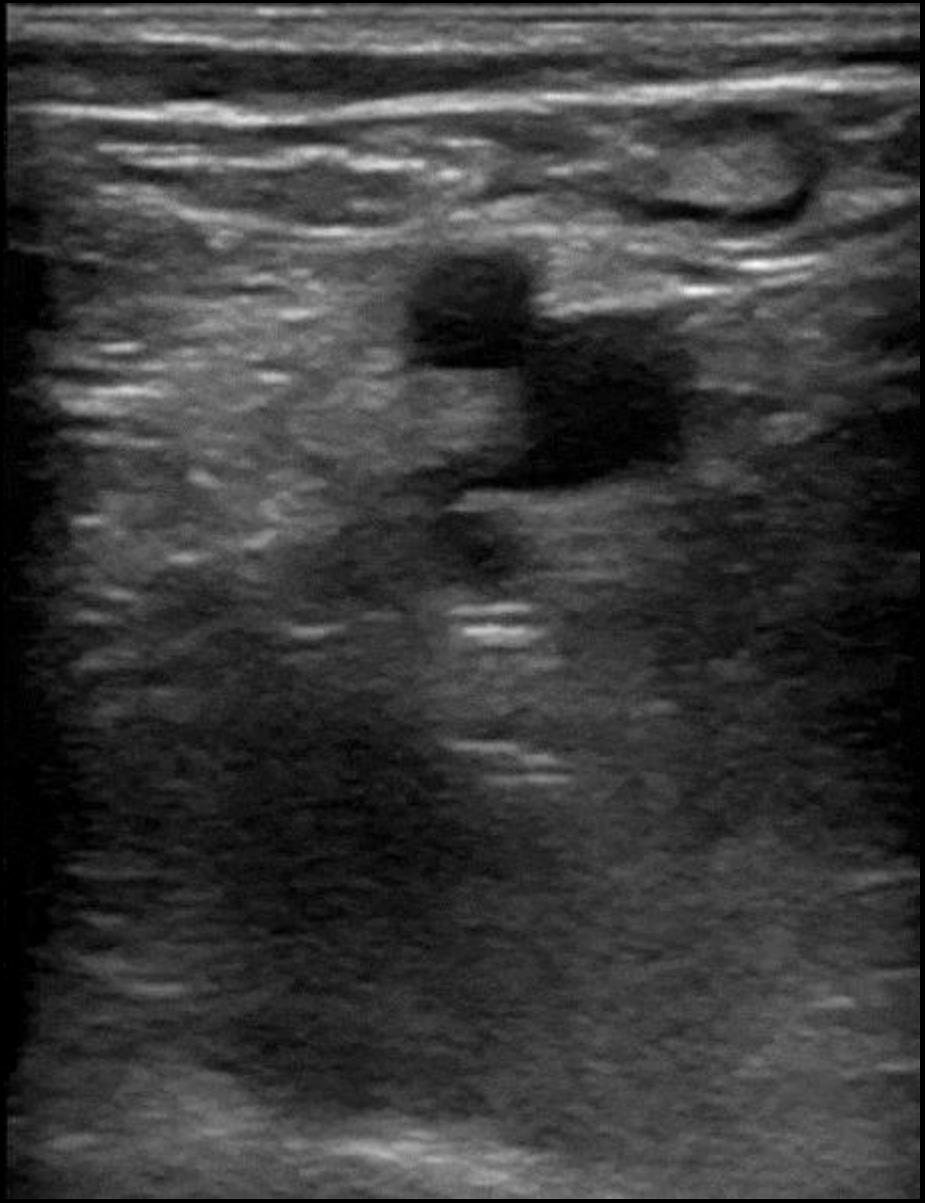

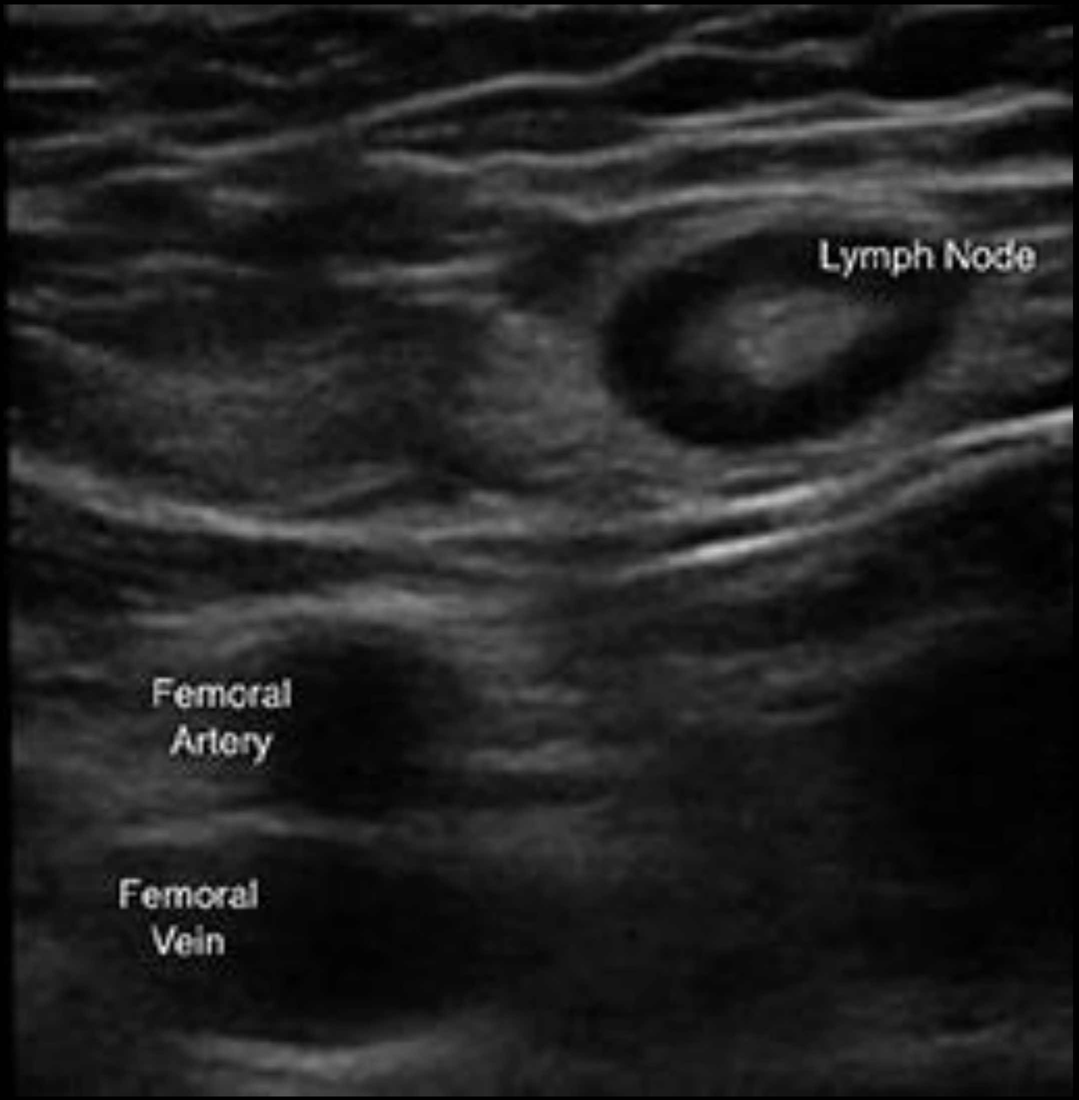
Lymph Node
Femoral
Artery
Femoral
Vein

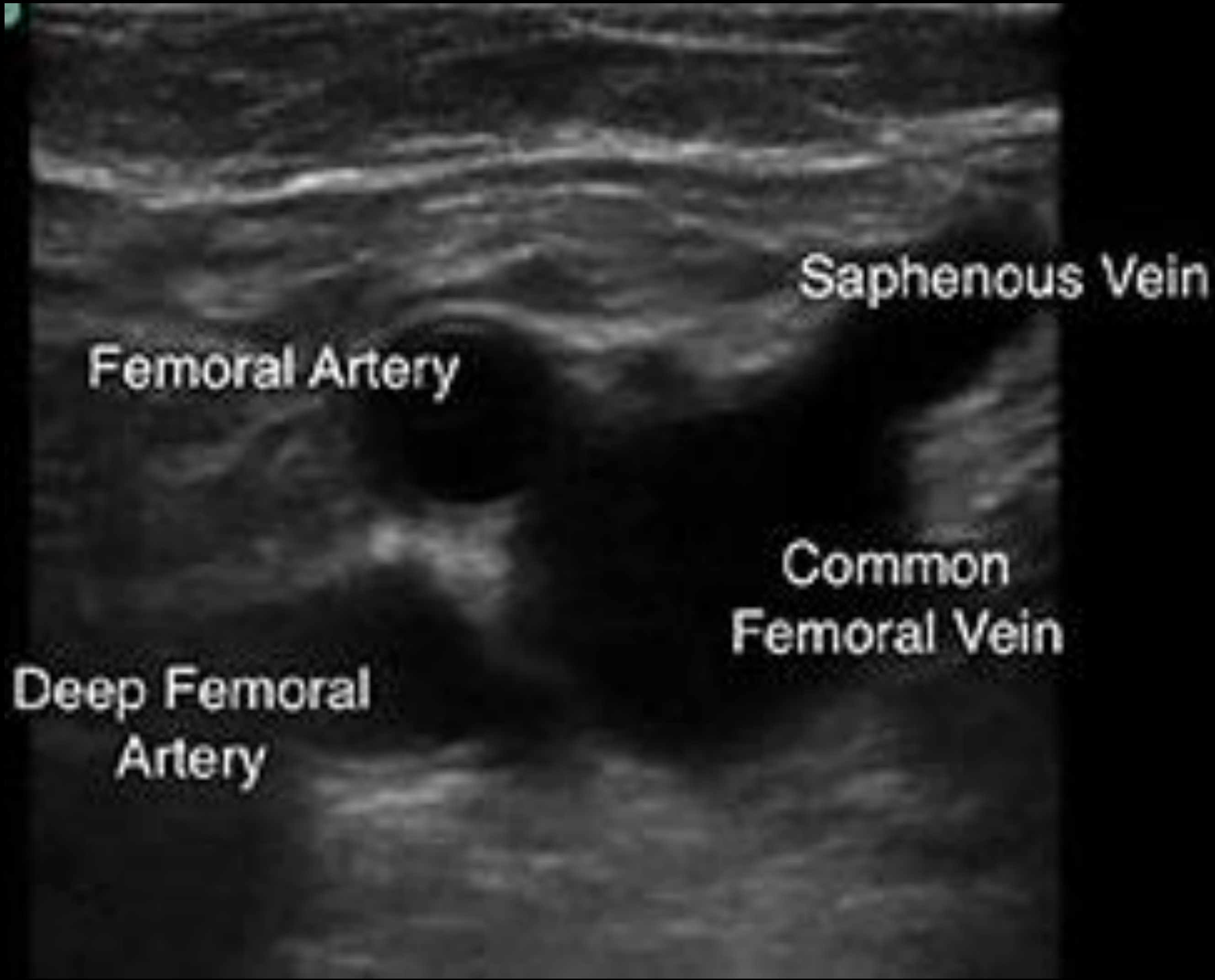
Saphenous Vein
Femoral Artery
Common
Femoral Vein
Deep Femoral
Artery

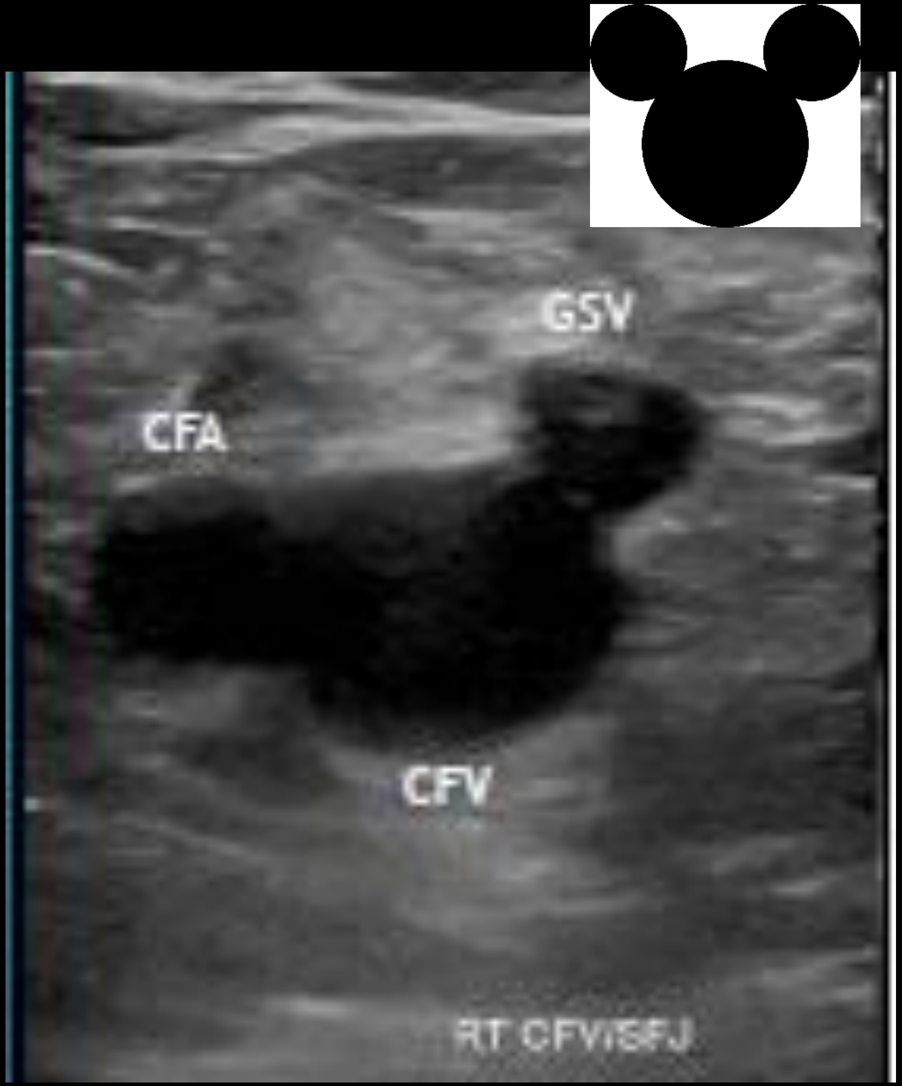
GSV
CFA
CFV
RT CFV/SFJ

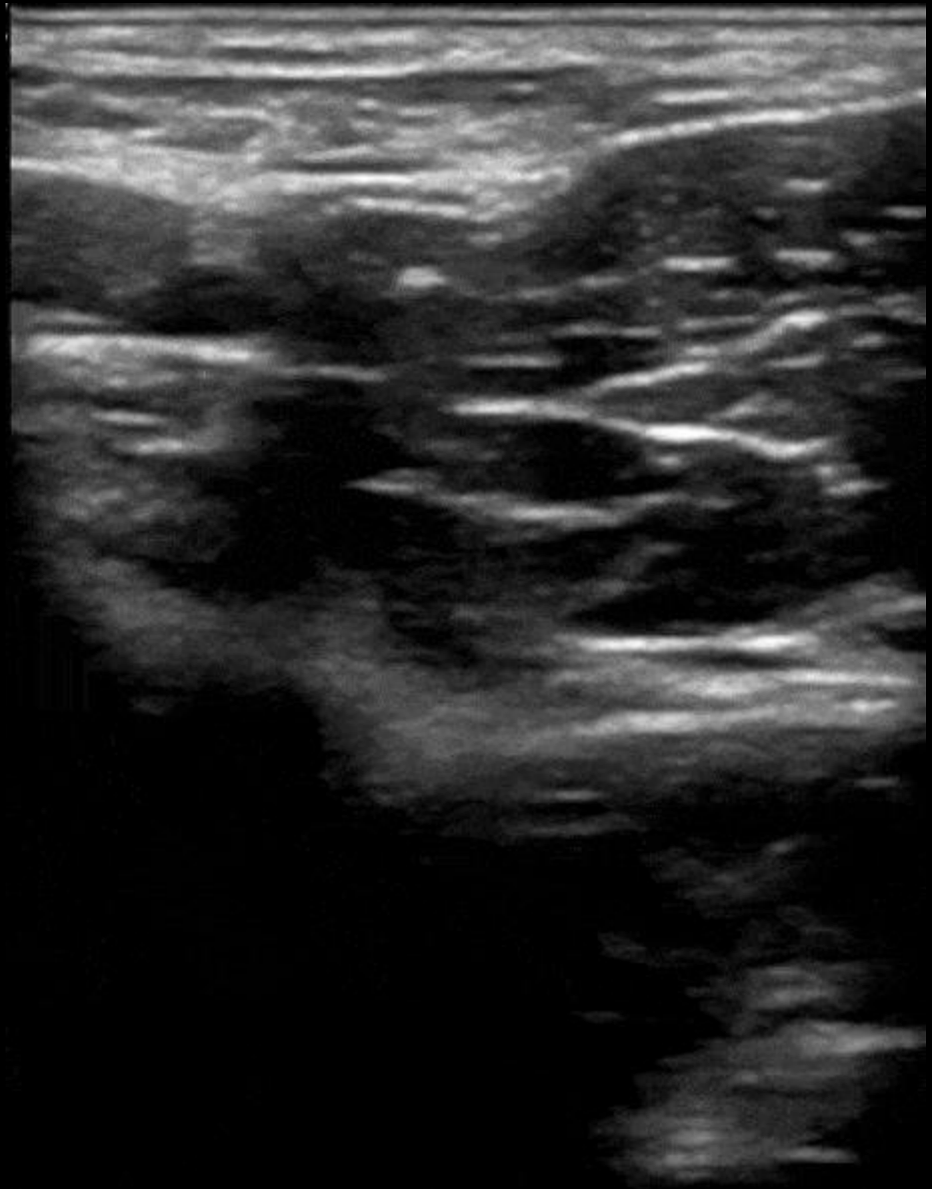

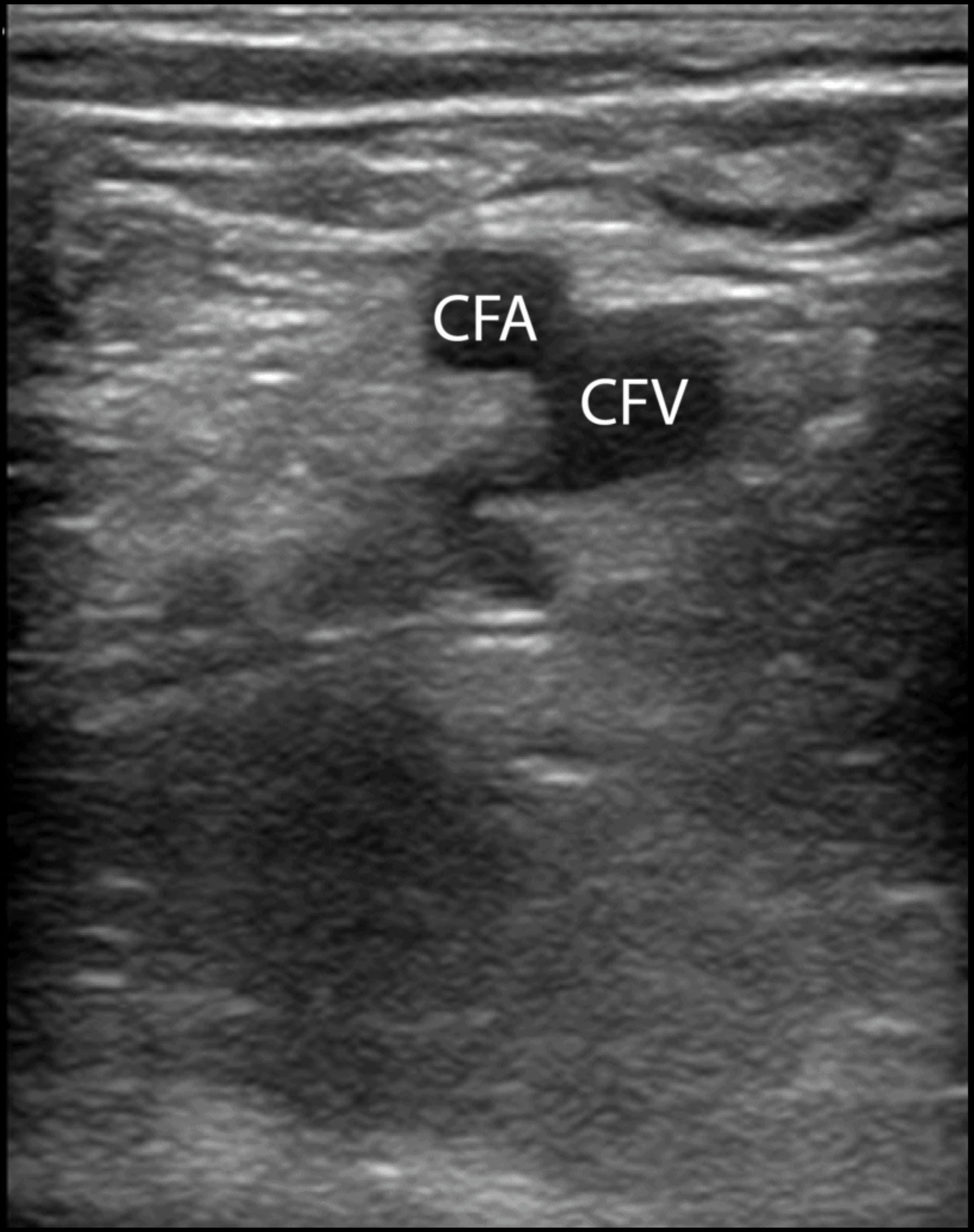
CFA
CFV

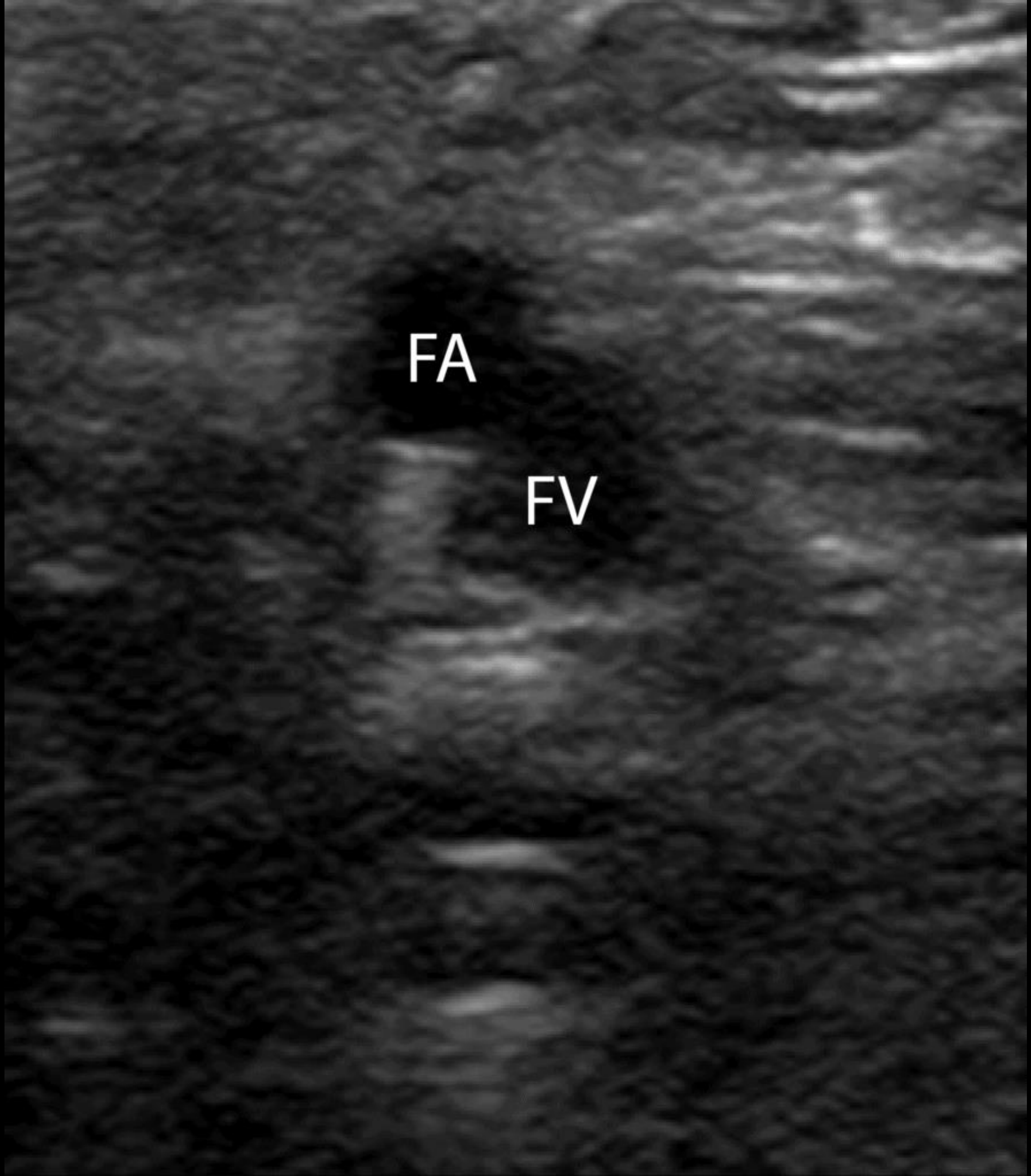
FA
FV

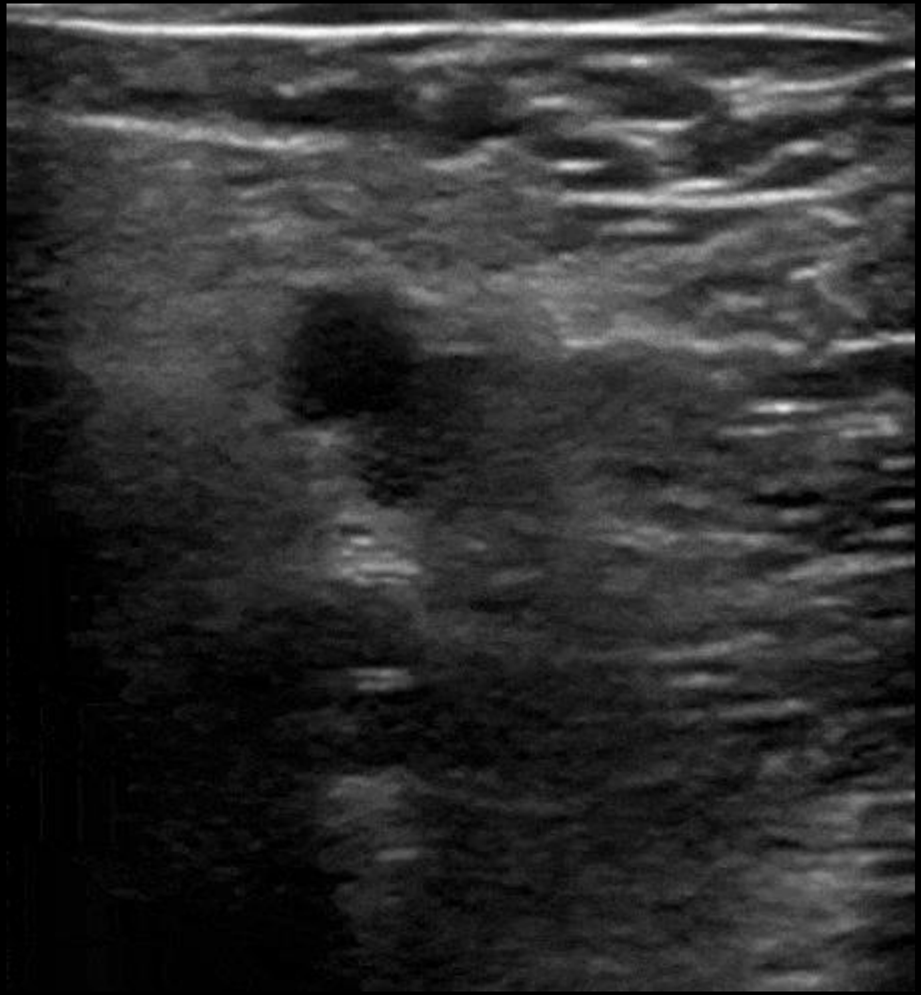

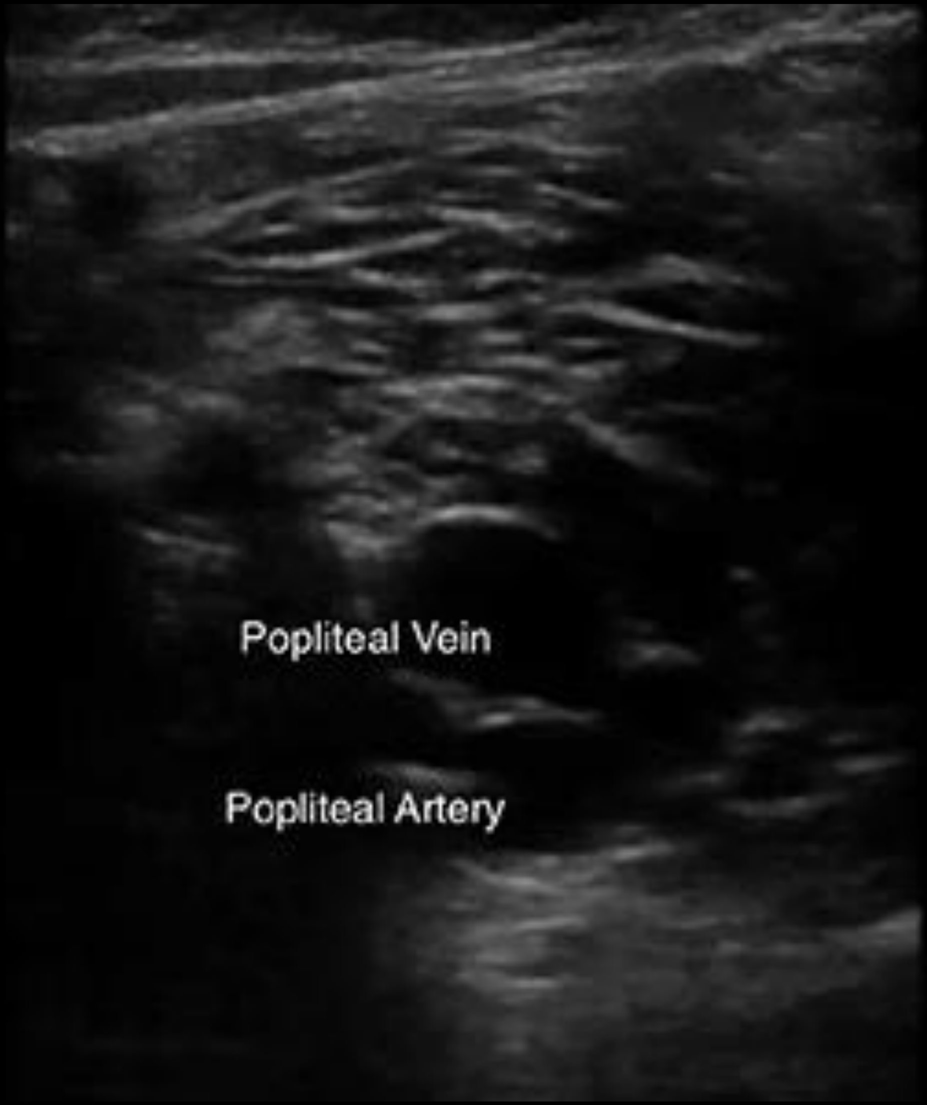
Popliteal Vein
Popliteal Artery

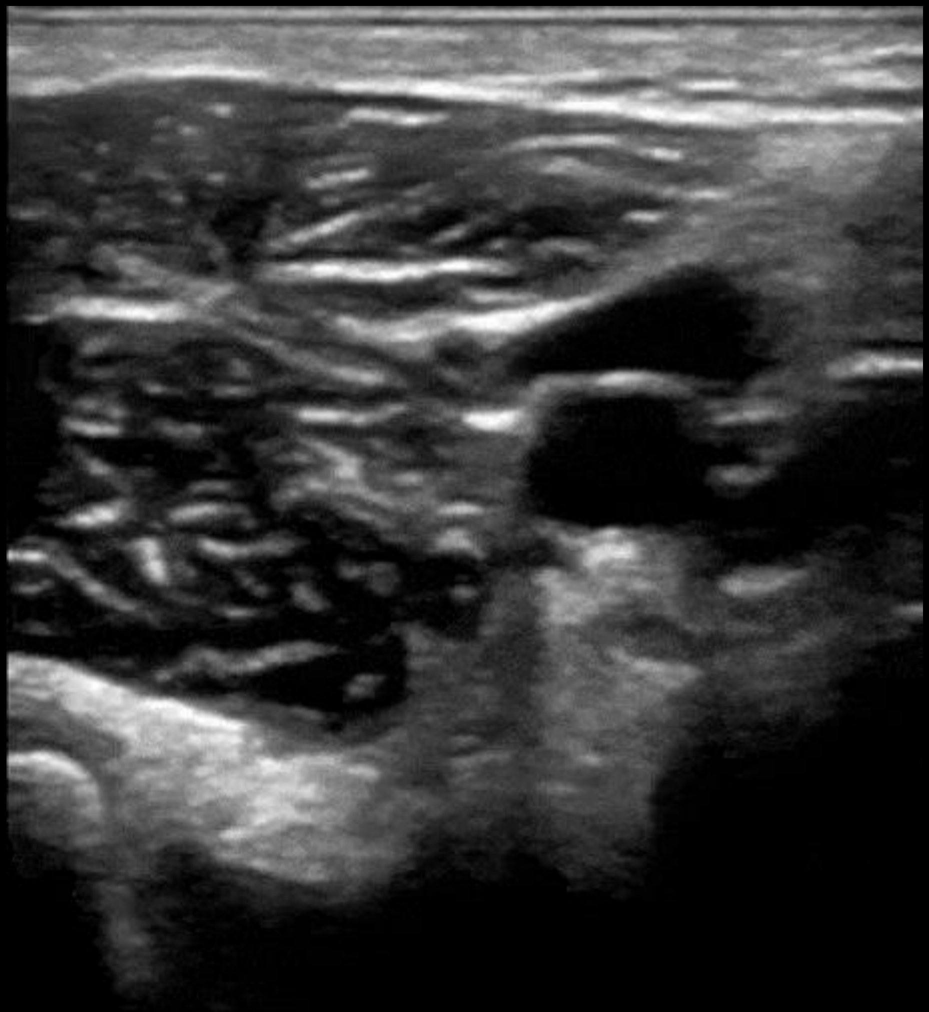

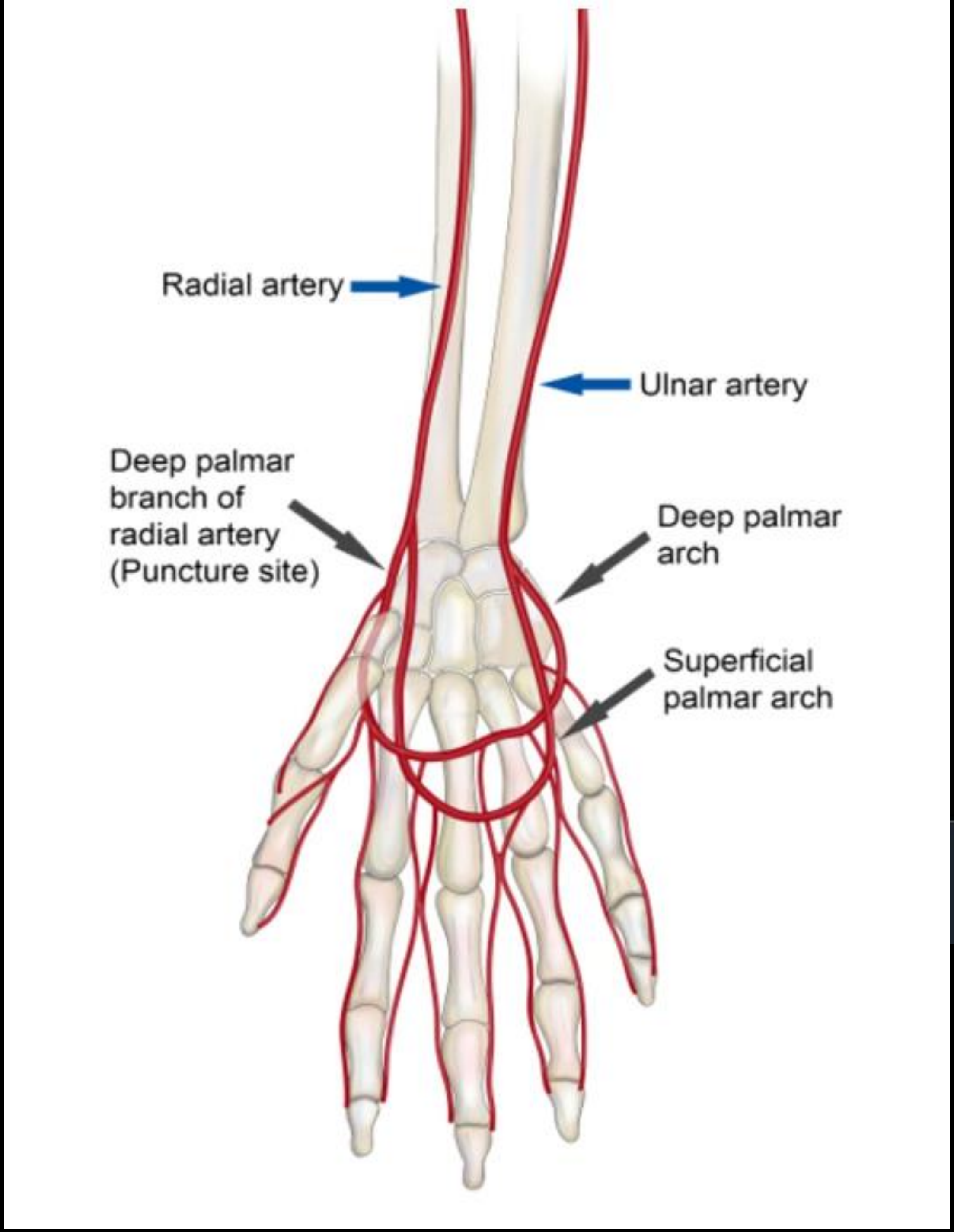
Radial artery
Ulnar artery
Deep palmar branch of radial artery (Puncture site)
Deep palmar arch
Superficial palmar arch

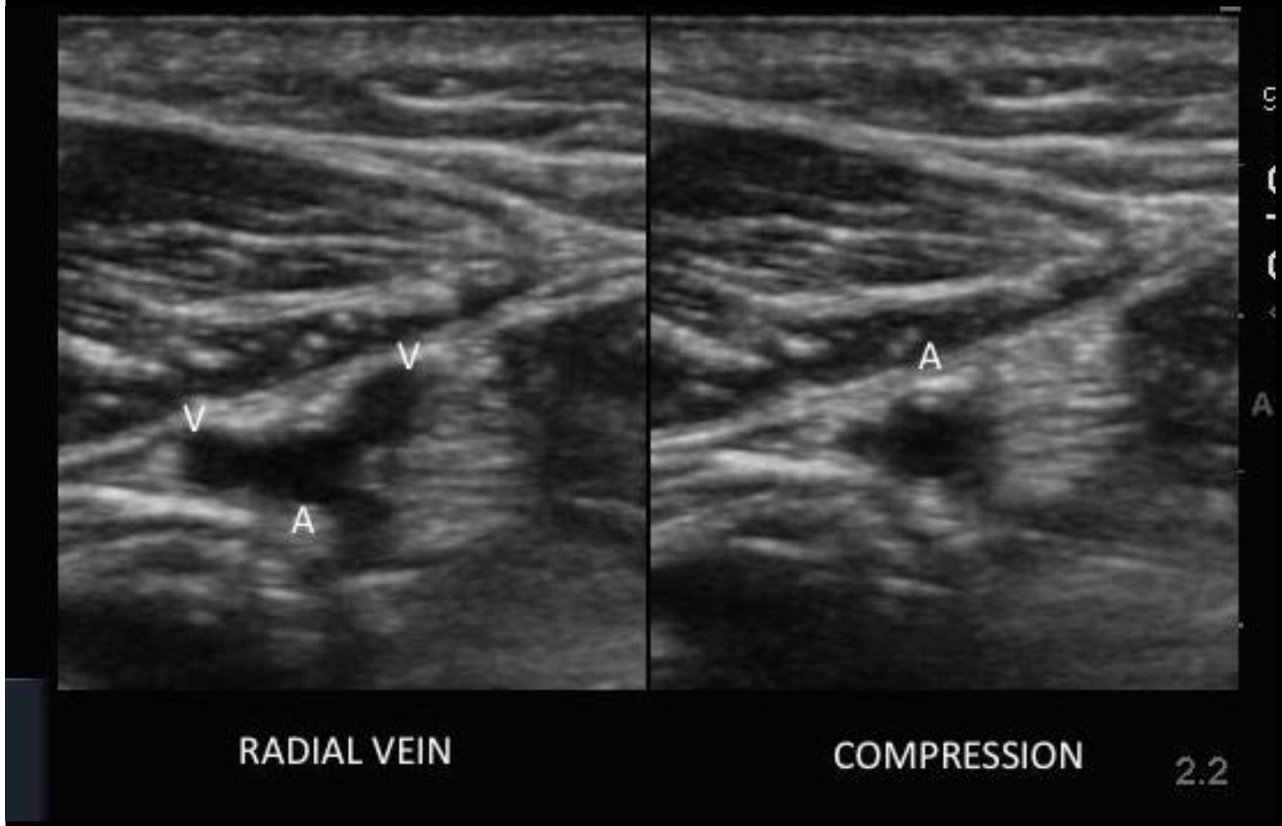
V
V
A
A
RADIAL VEIN
COMPRESSION
2.2

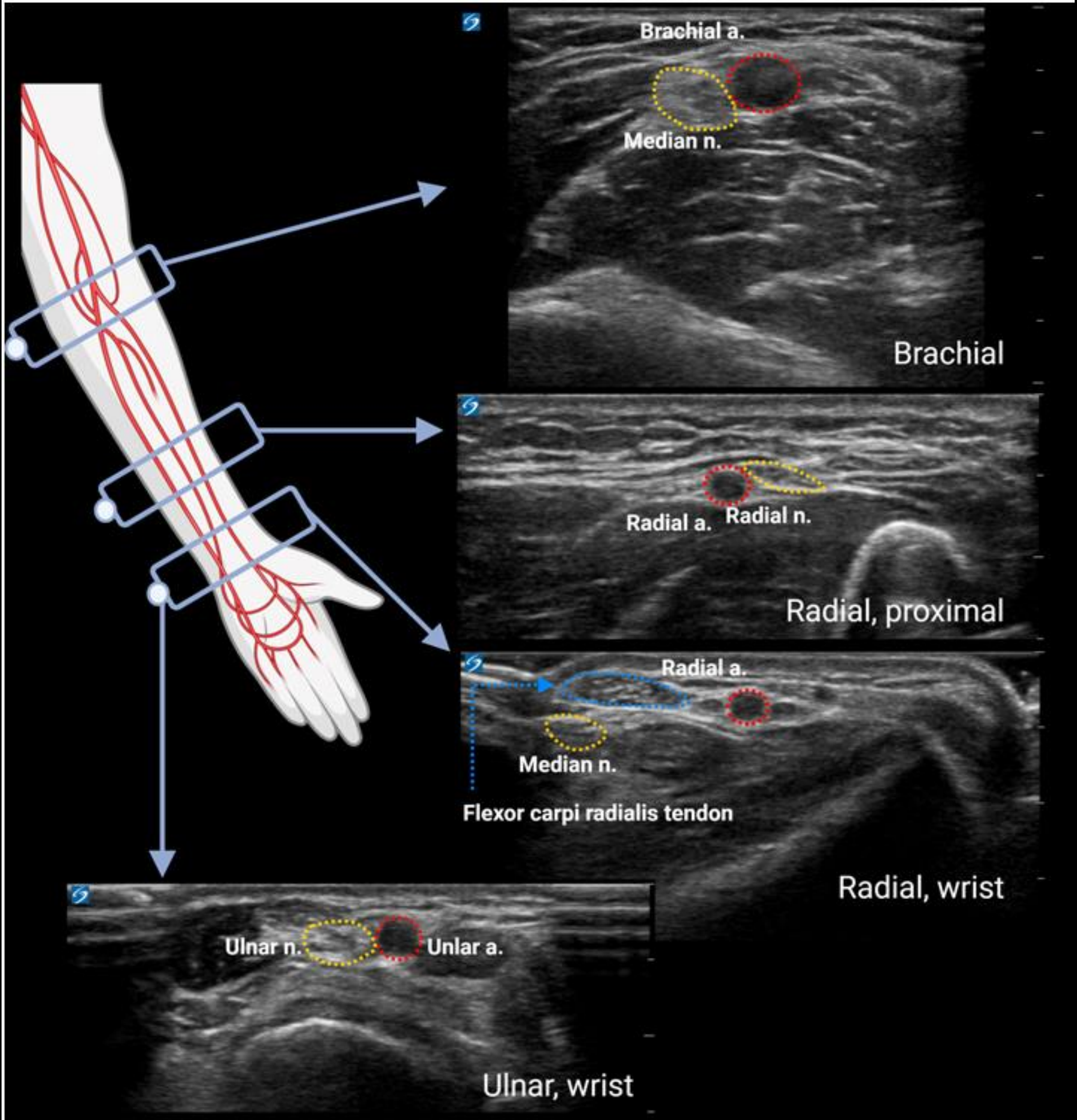

Brachial a.
Median n.
Brachial
Radial a.
Radial n.
Radial, proximal
Radial a.
Median n.
Flexor carpi radialis tendon
Radial, wrist
Ulnar n.
Unlar a.
Ulnar, wrist

Common Vascular Complications & Pitfalls

Hematoma or Pseudoaneurysm?

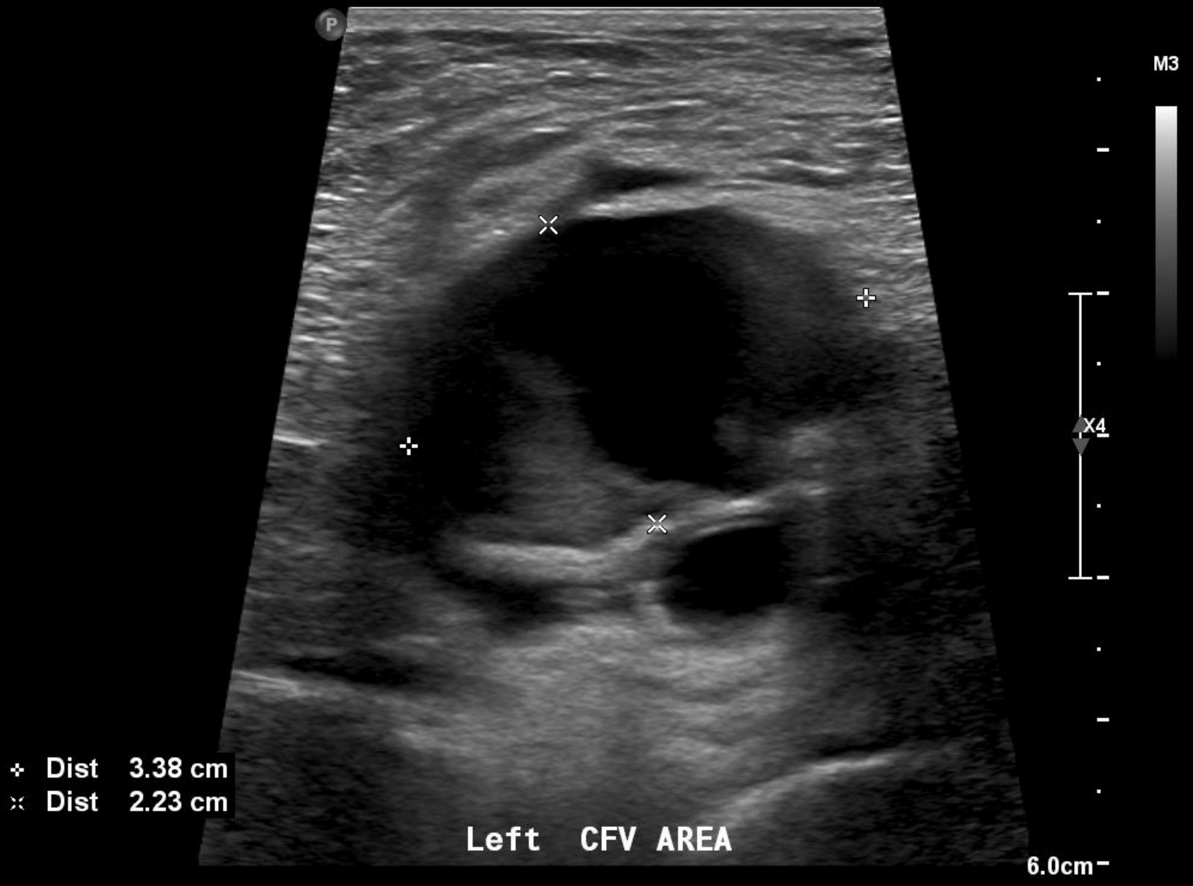

Hematoma or Pseudoaneurysm?

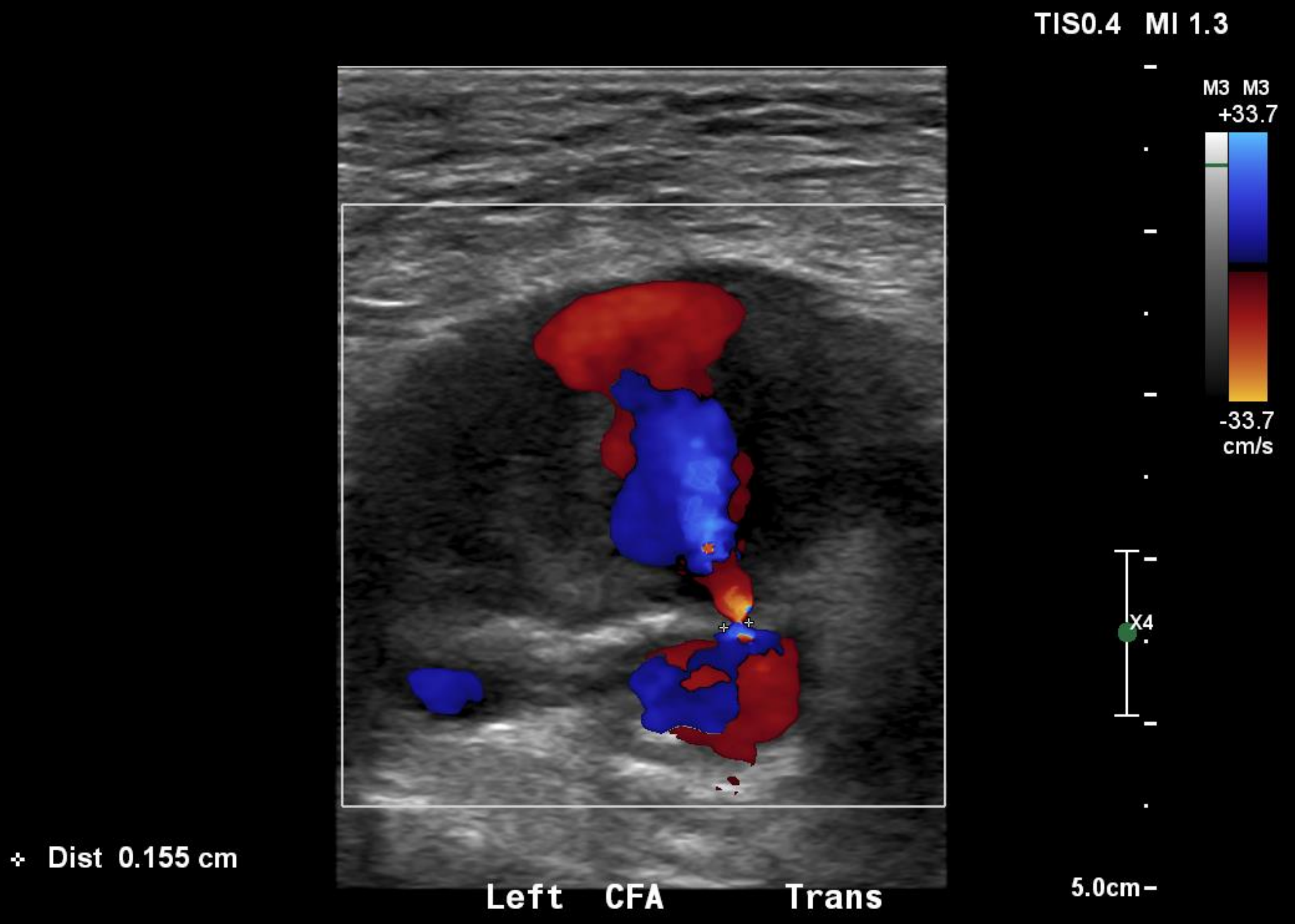

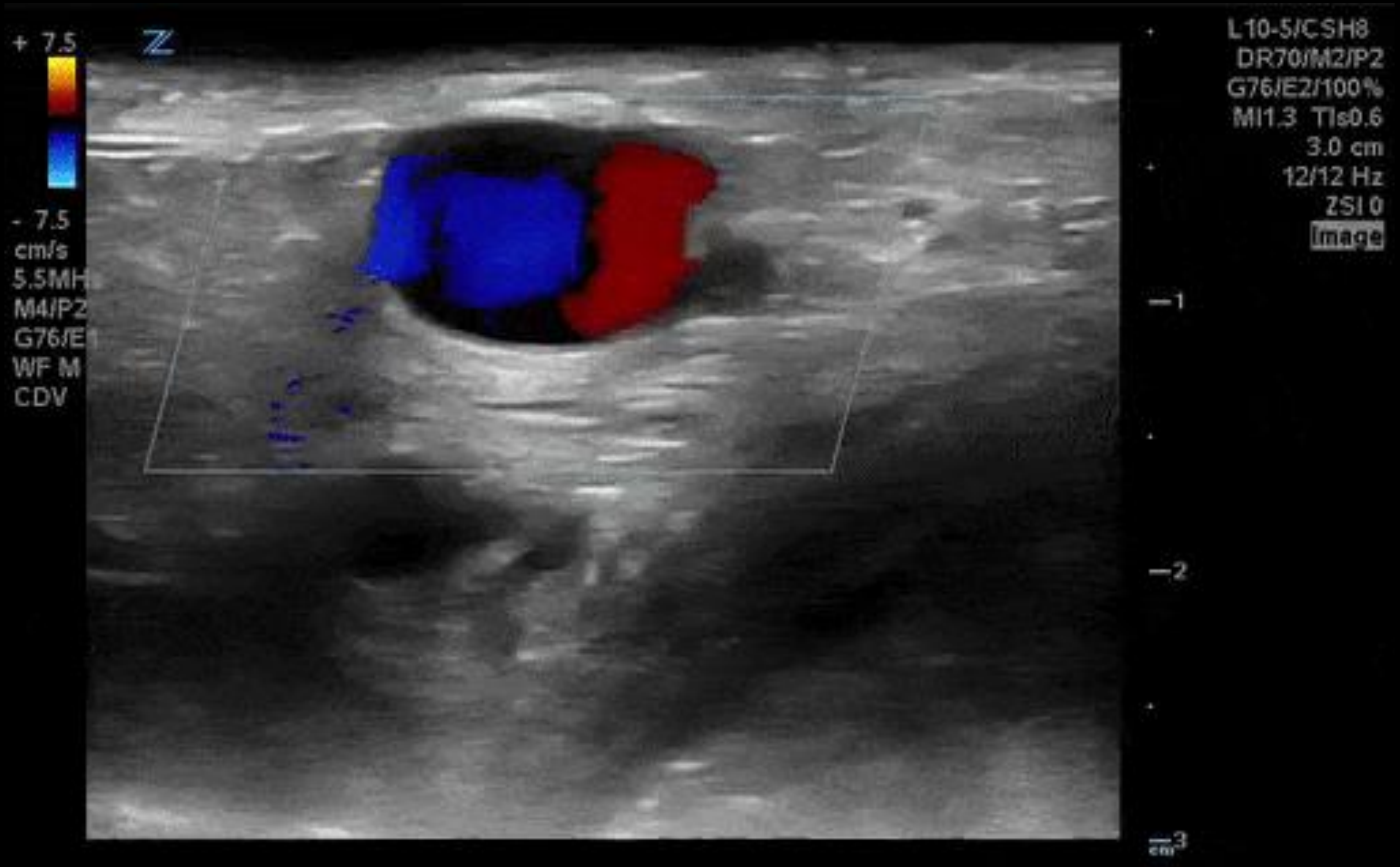
+ 7.5
- 7.5
cm/s
5.5MH
M4/P2
G76/E1
WF M
CDV
L10-5/CSH8
DR70/M2/P2
G76/E2/100%
MI1.3 TIs0.6
3.0 cm
12/12 Hz
ZSI 0
Image
1
2
3
cm

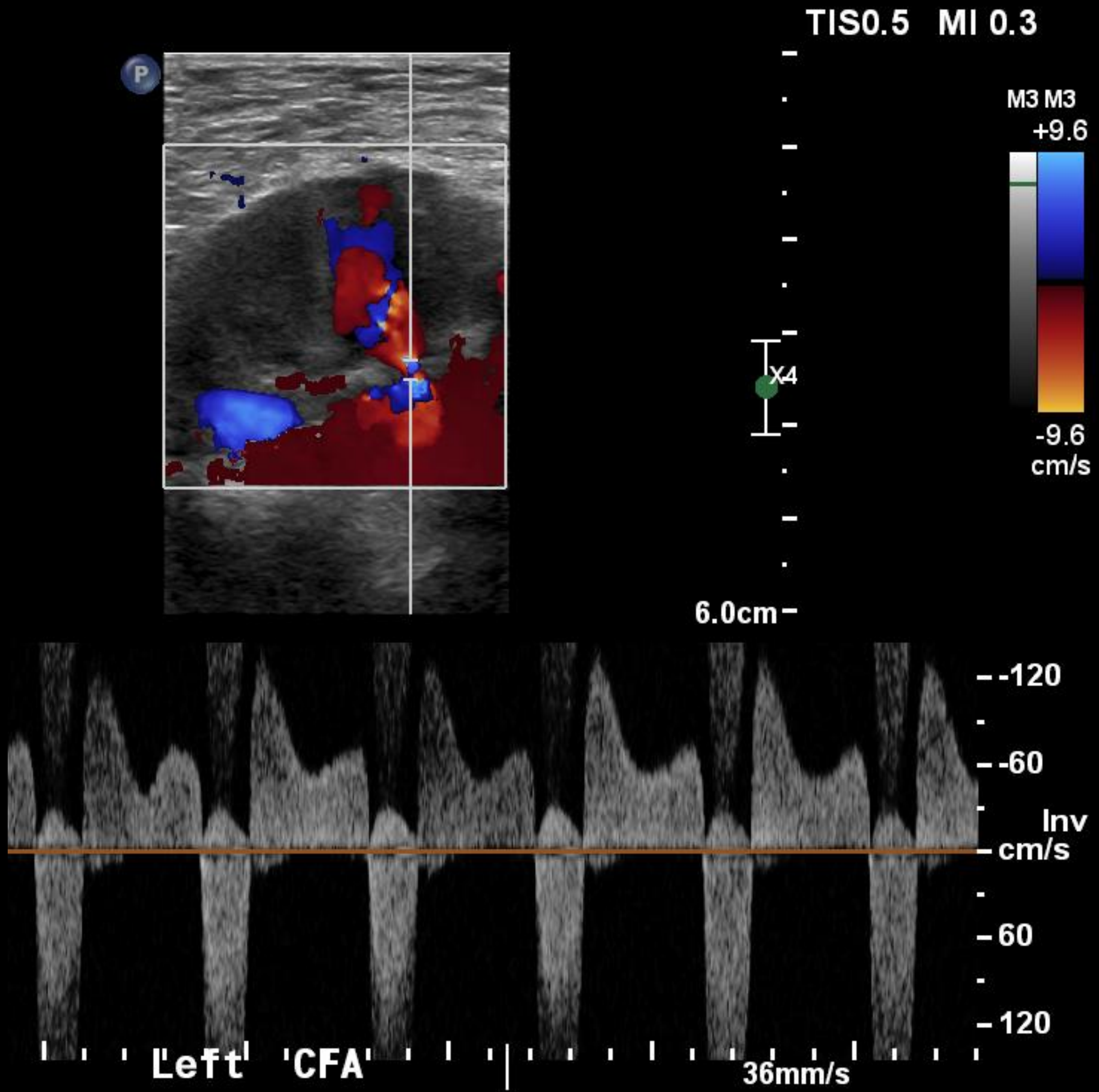
TIS0.5 MI 0.3
M3 M3
+9.6
-9.6
cm/s
X4
6.0cm
-120
-60
Inv
cm/s
60
120
Left CFA
36mm/s

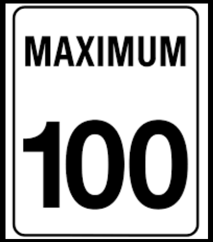
MAXIMUM
100

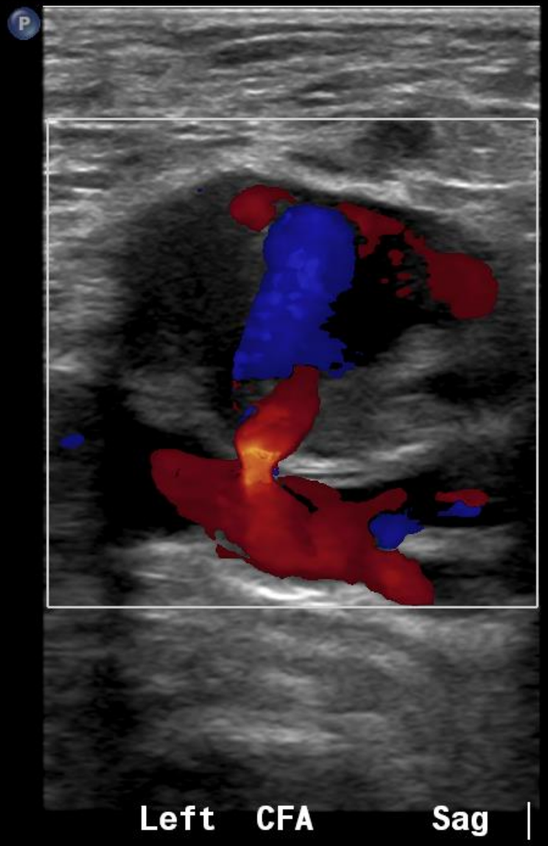

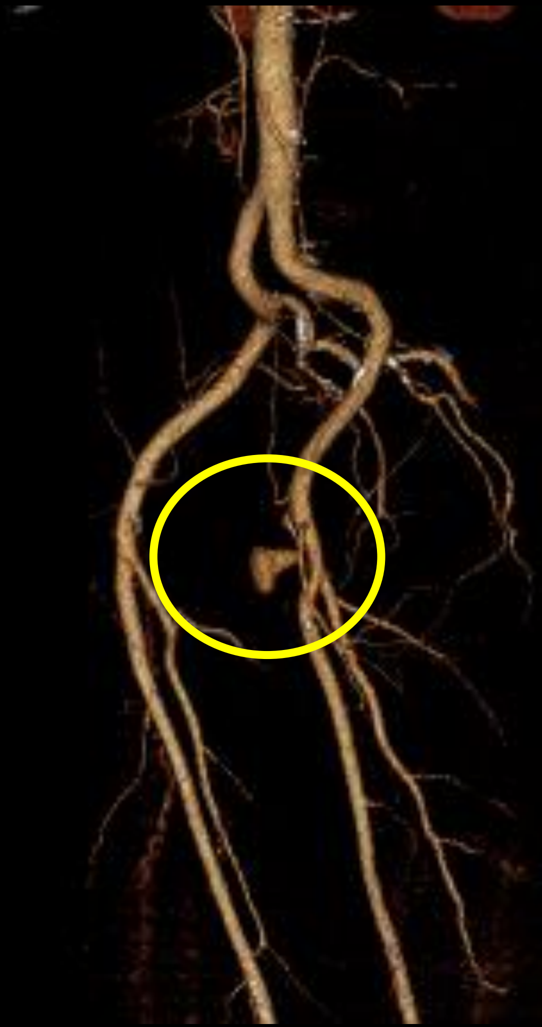
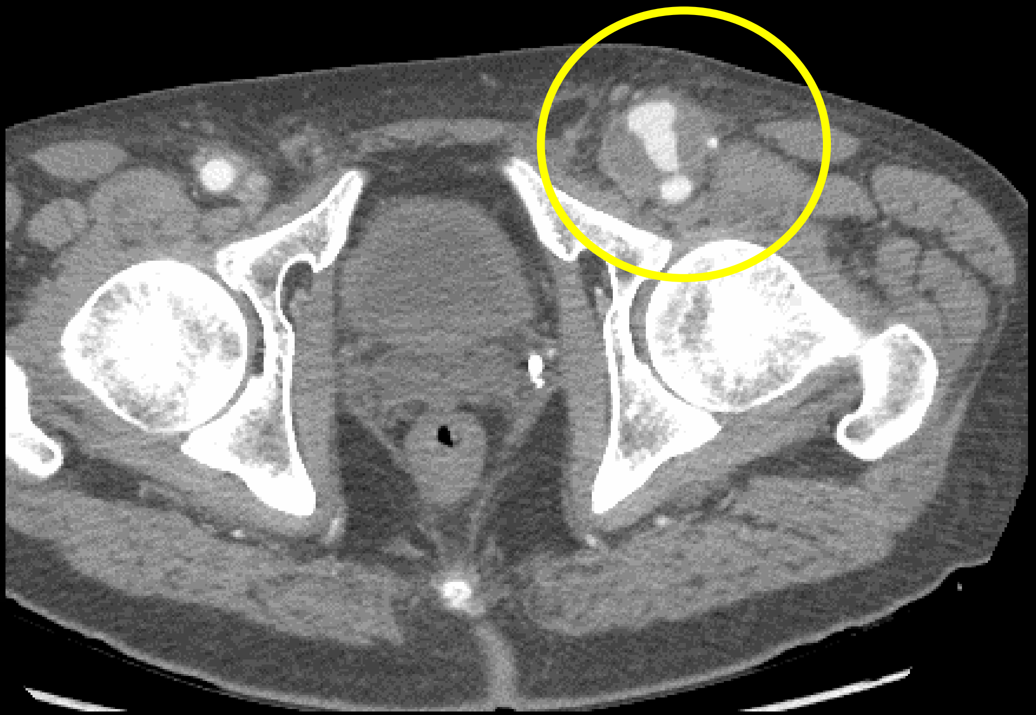

Pseudoaneurysm of the CFA

Pseudoaneurysm – Key features

- Grayscale: rounded hypoechoic structure, close to vessel
- Ying-Yang sign (bidirectional flow)
- Neck
  - Connects to adjacent vessel
  - Aliasing (turbulent flow)
  - High velocity
  - Bi-directional flow

Pseudoaneurysm: Ying-Yang

- Bidirectional flow due to swirling of blood in the sac

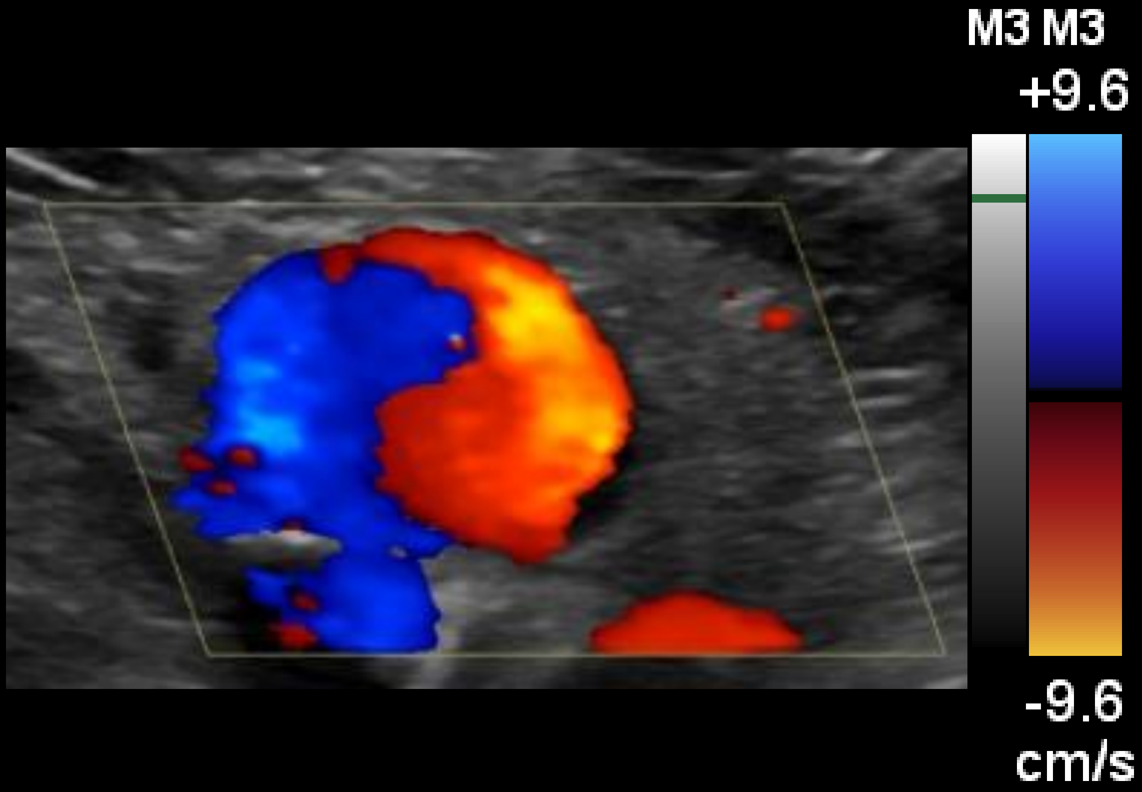

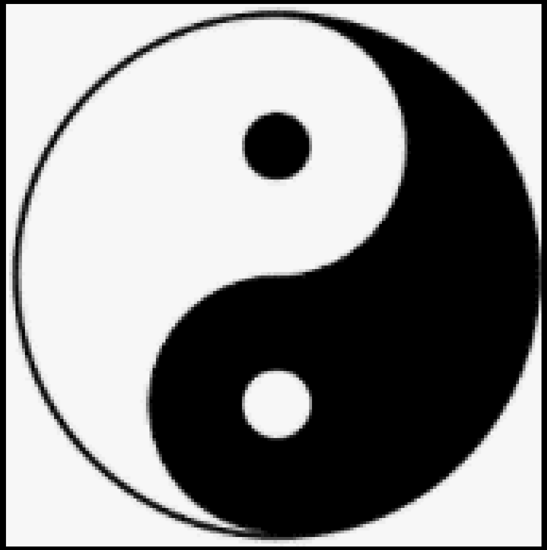

Pseudoaneurysm: Neck

- Linear hypoechoic tract leading from a vessel to the sac
- Can be short or long, narrow or thick
- Will show:
  - Turbulent flow or aliasing on colour evaluation
  - High velocities
  - Bi-directional flow

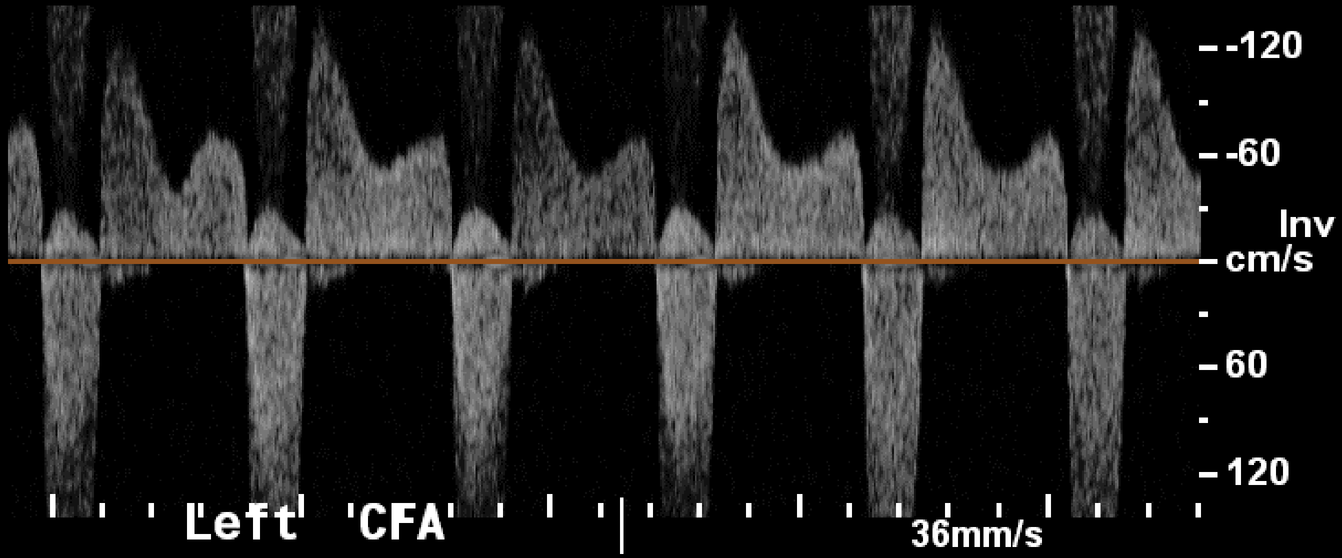

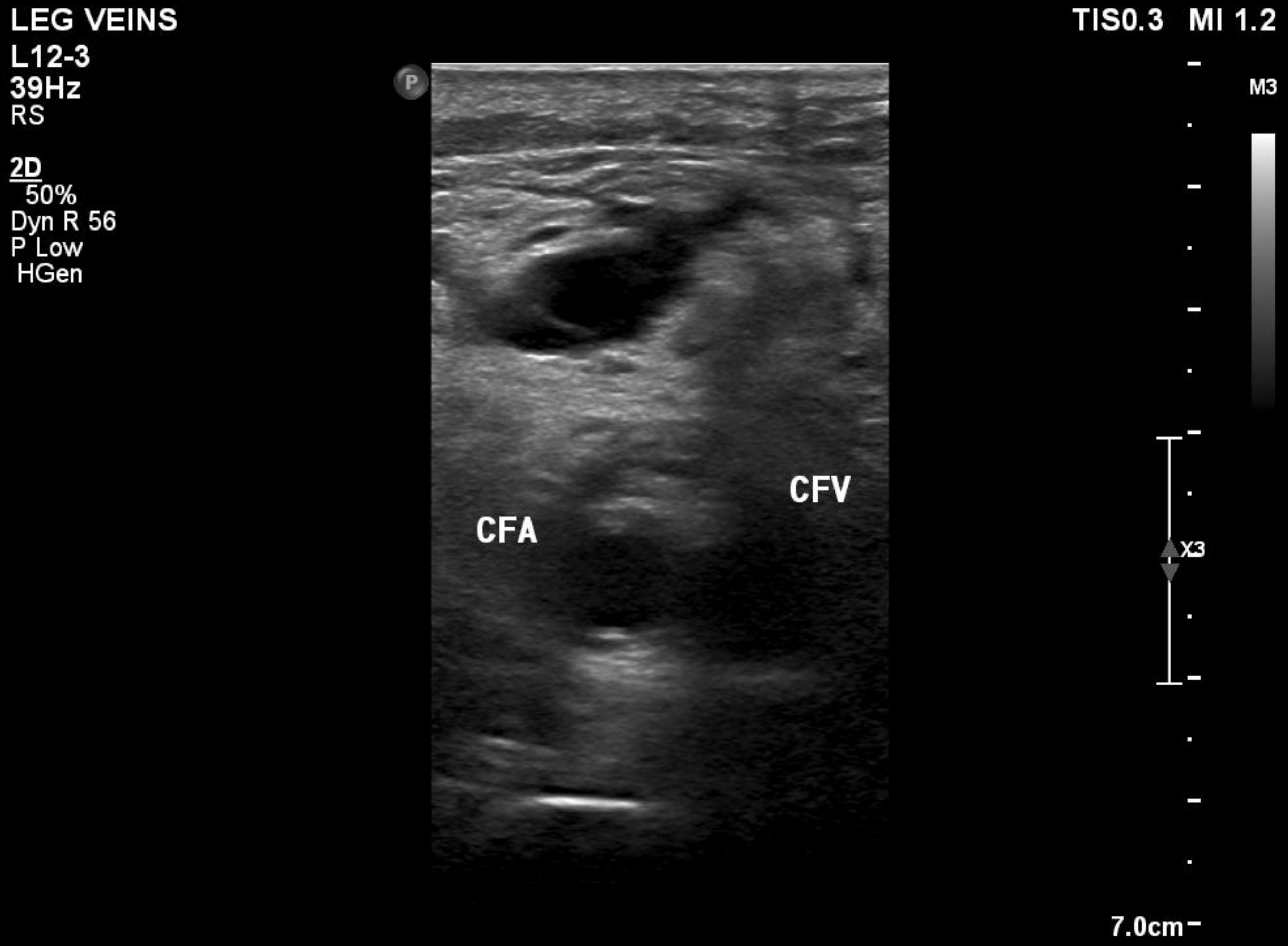
LEG VEINS
L12-3
39Hz
RS
2D
50%
Dyn R 56
P Low
HGen
TIS0.3 MI 1.2
M3
P
CFA
CFV
X3
7.0cm

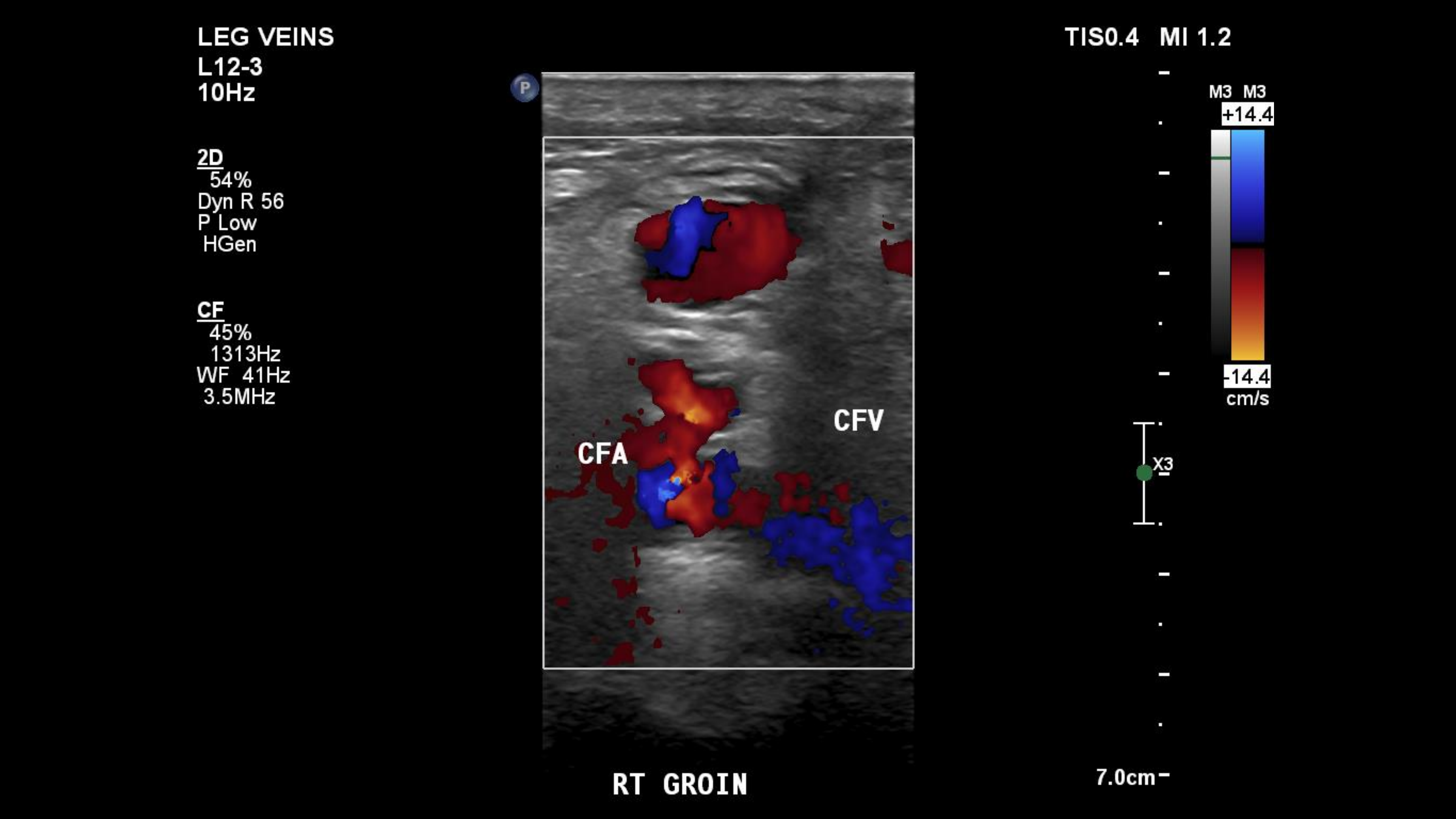
LEG VEINS
L12-3
10Hz
2D
54%
Dyn R 56
P Low
HGen
CF
45%
1313Hz
WF 41Hz
3.5MHz
TIS0.4 MI 1.2
M3 M3
+14.4
-14.4
cm/s
X3
CFA
CFV
RT GROIN
7.0cm

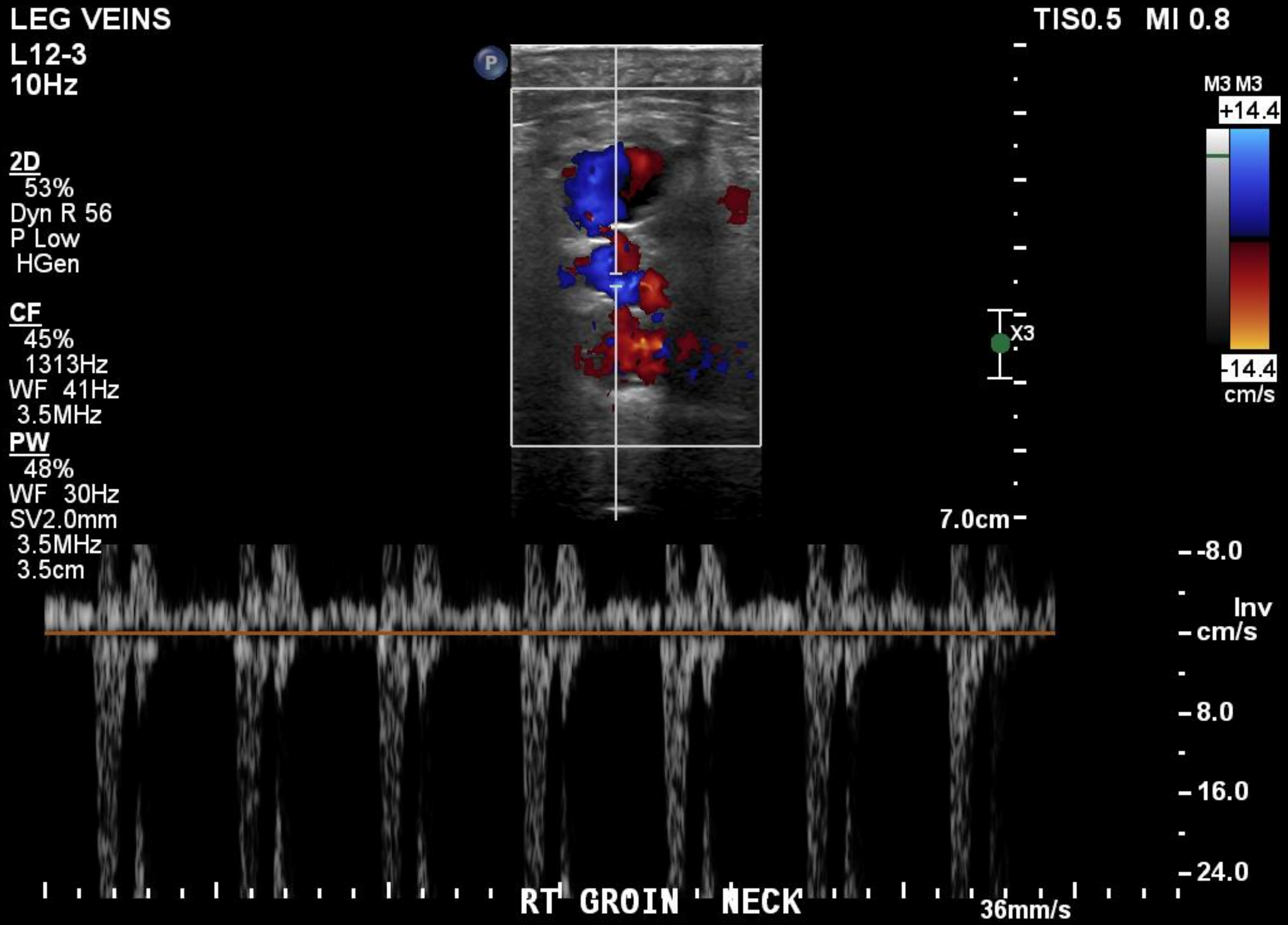
LEG VEINS
L12-3
10Hz
2D
53%
Dyn R 56
P Low
HGen
CF
45%
1313Hz
WF 41Hz
3.5MHz
PW
48%
WF 30Hz
SV2.0mm
3.5MHz
3.5cm
TIS0.5 MI 0.8
M3 M3
+14.4
-14.4
cm/s
X3
7.0cm
-8.0
Inv
cm/s
8.0
16.0
24.0
RT GROIN NECK
36mm/s

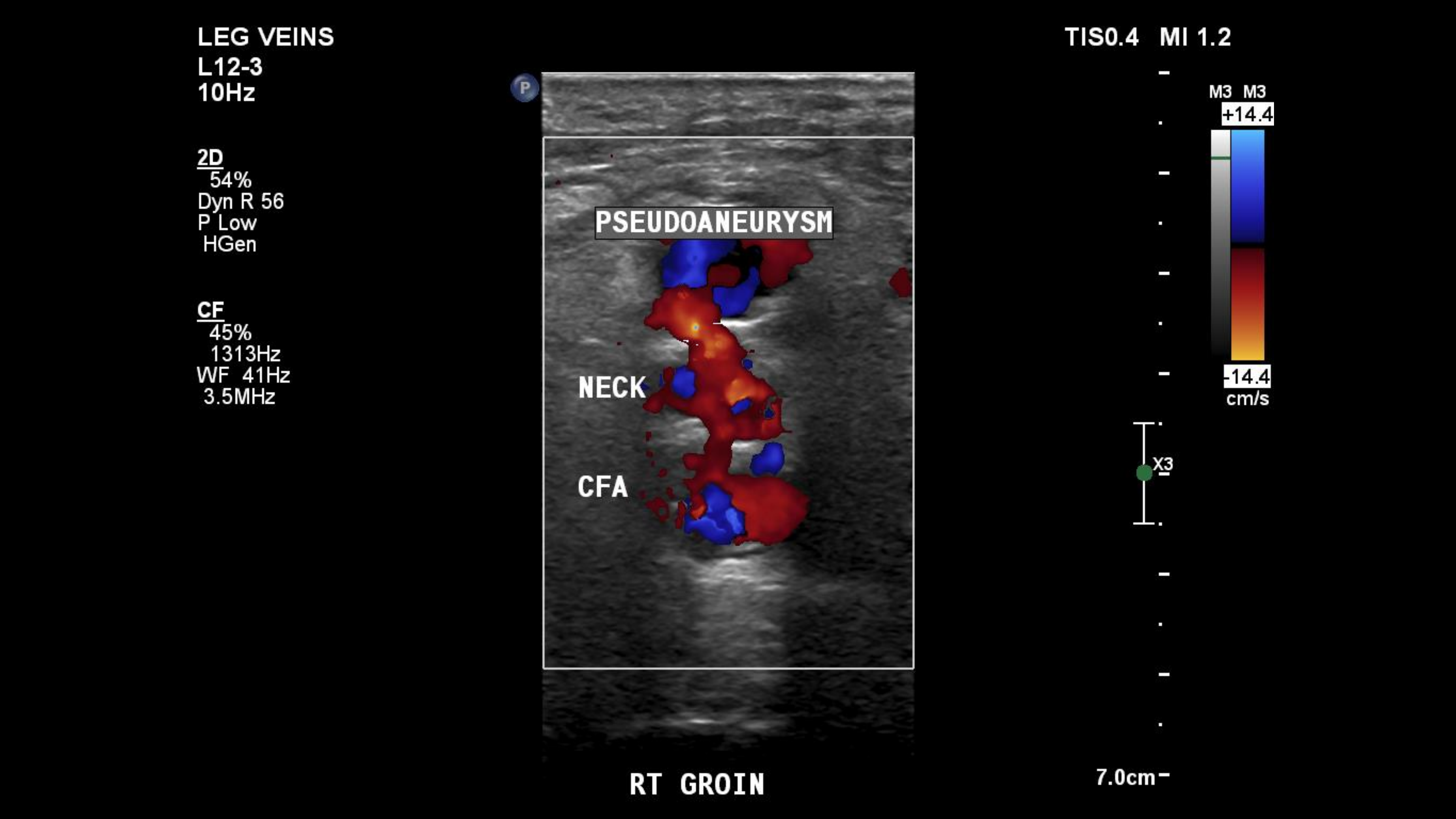
LEG VEINS
L12-3
10Hz
2D
54%
Dyn R 56
P Low
HGen
CF
45%
1313Hz
WF 41Hz
3.5MHz
TIS0.4 MI 1.2
M3 M3
+14.4
-14.4
cm/s
X3
7.0cm
PSEUDOANEURYSM
NECK
CFA
RT GROIN

Pseudoaneurysms look the same anywhere

Liver

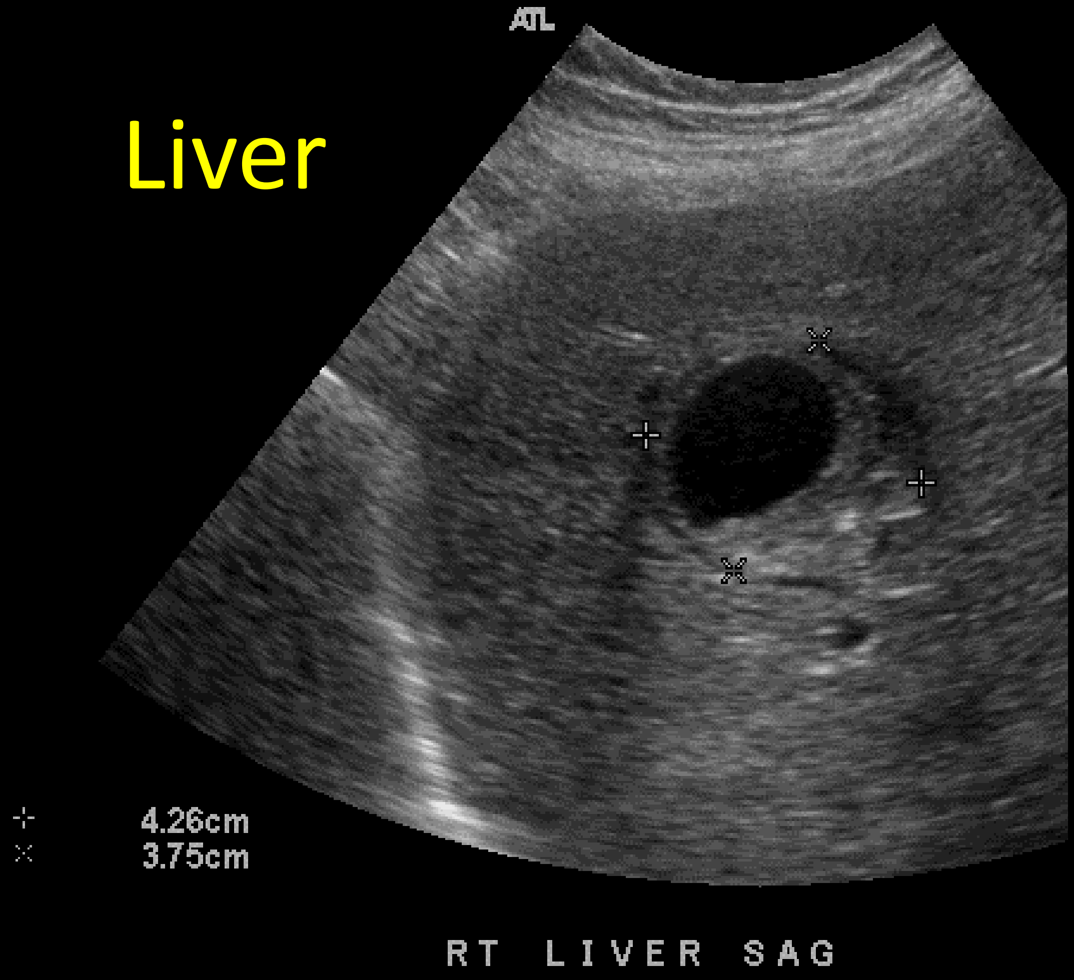

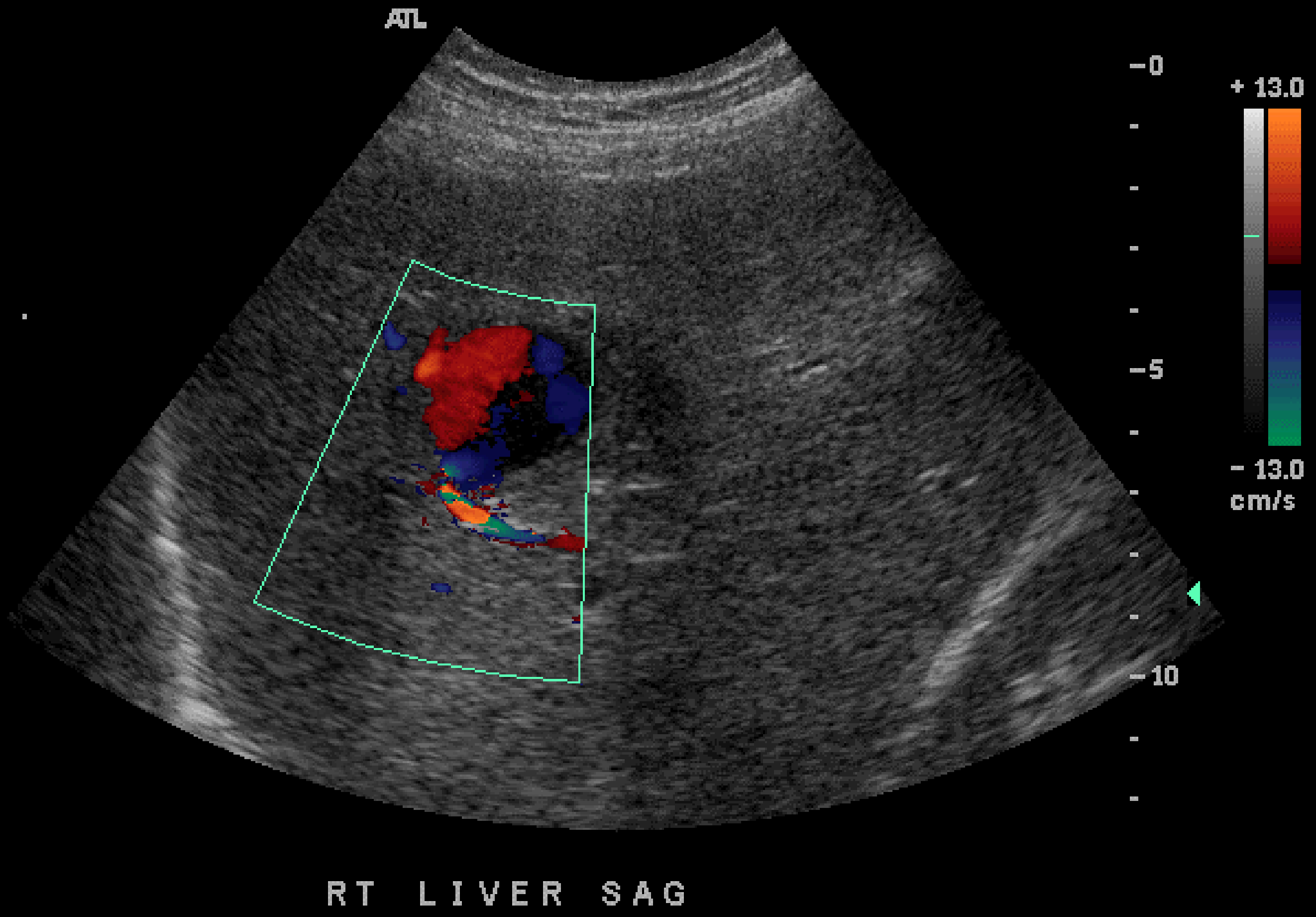

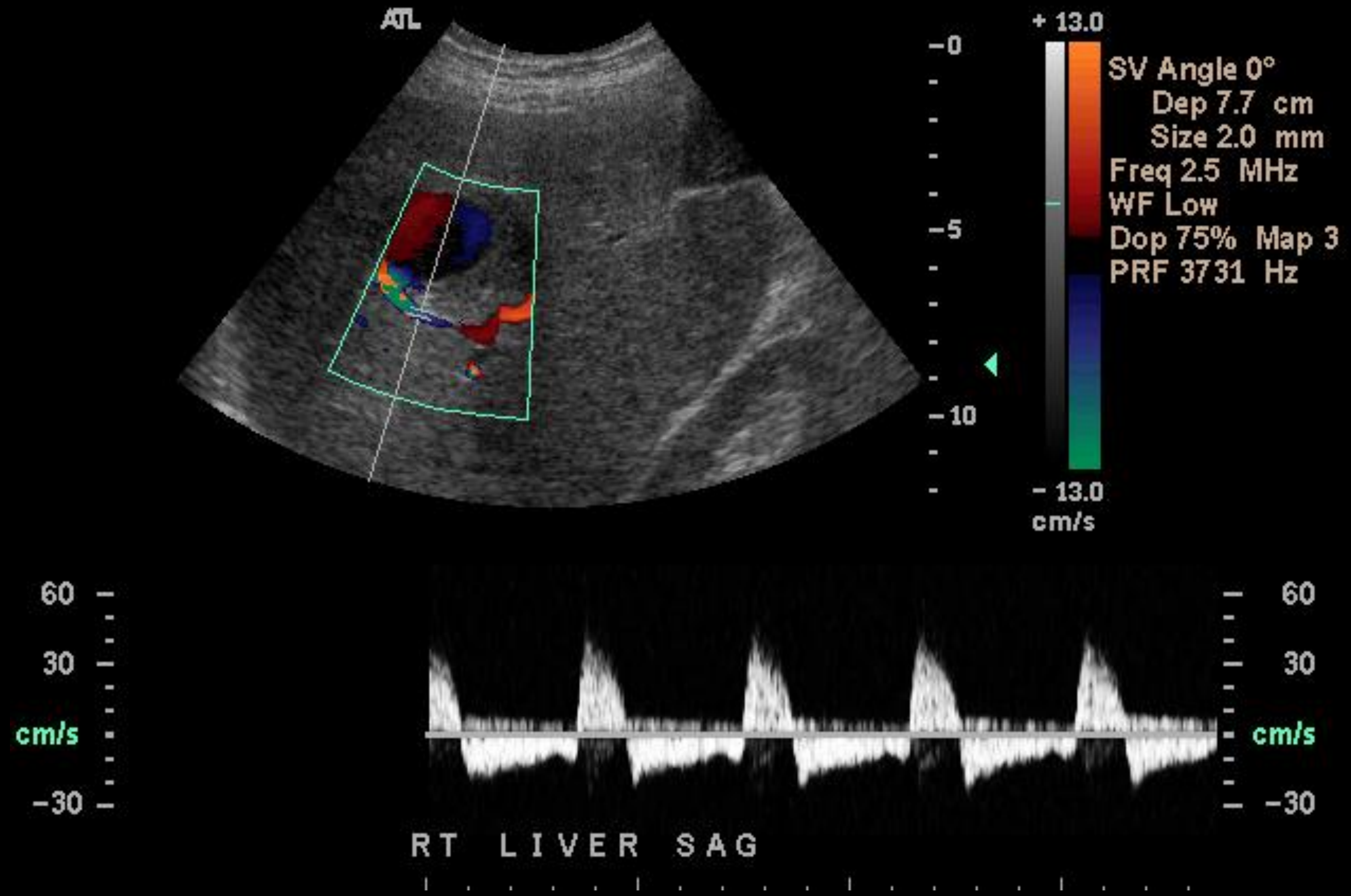
ATL
SV Angle 0°
Dep 7.7 cm
Size 2.0 mm
Freq 2.5 MHz
WF Low
Dop 75% Map 3
PRF 3731 Hz
+ 13.0
- 13.0
cm/s
0
5
10
60
30
cm/s
−30
RT LIVER SAG

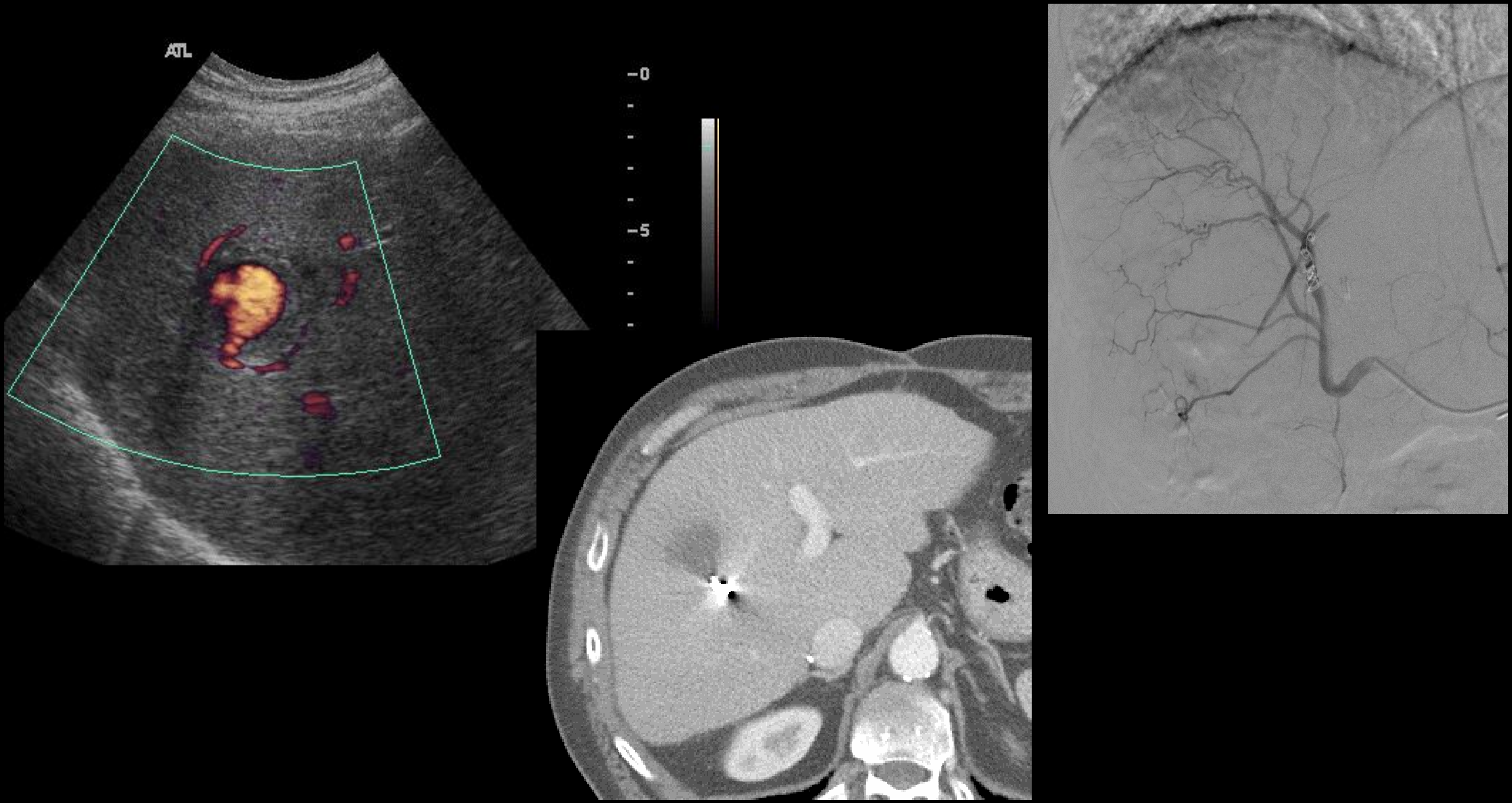
ATL
0
5

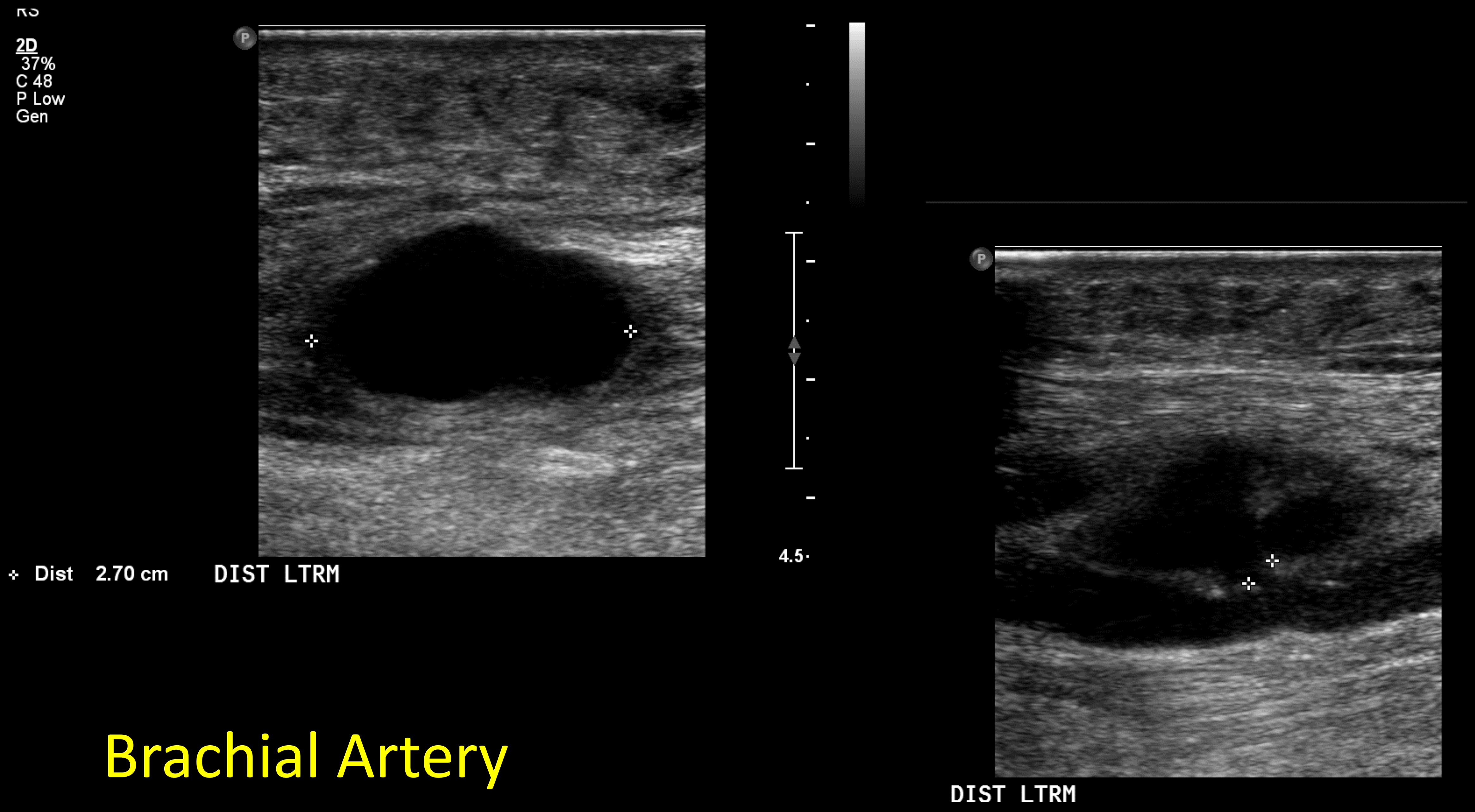

Brachial Artery

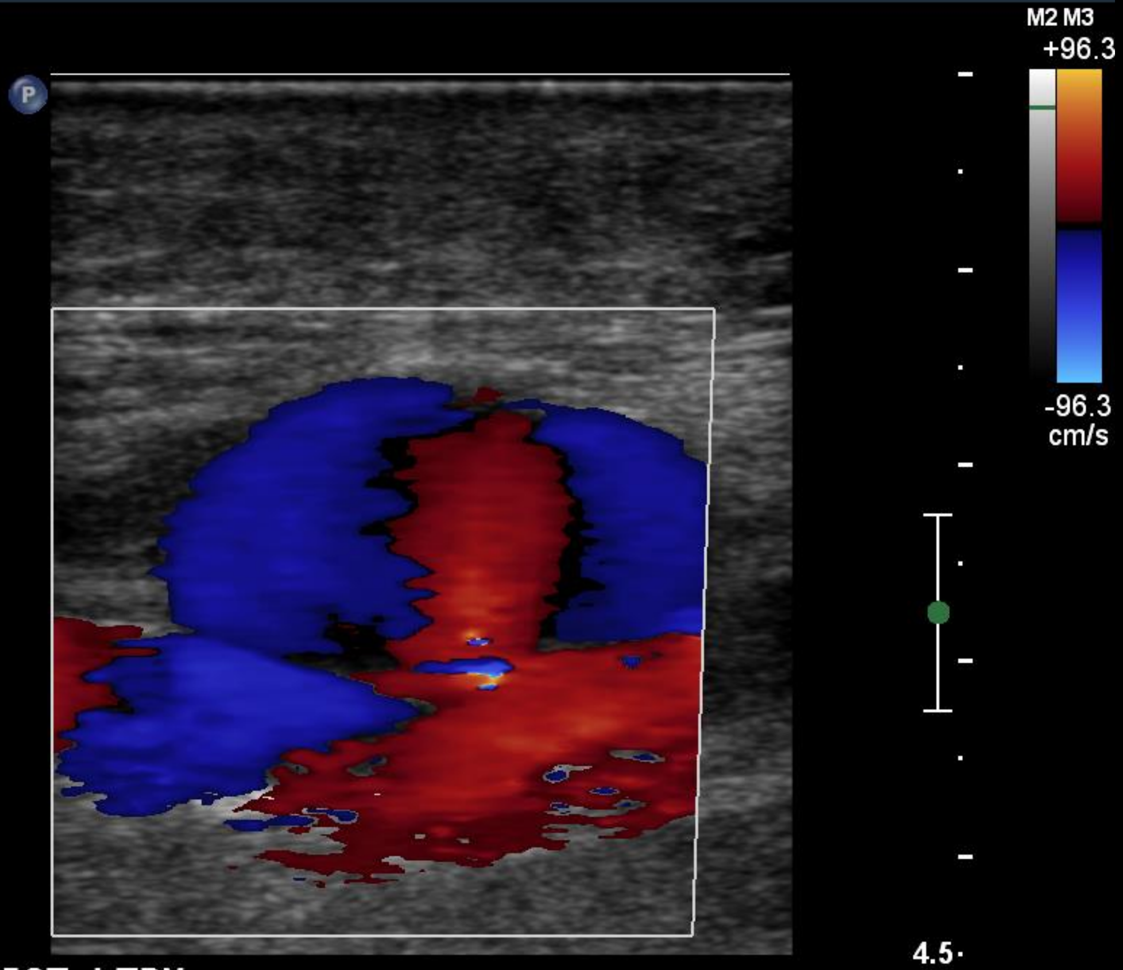

DIST LTRM

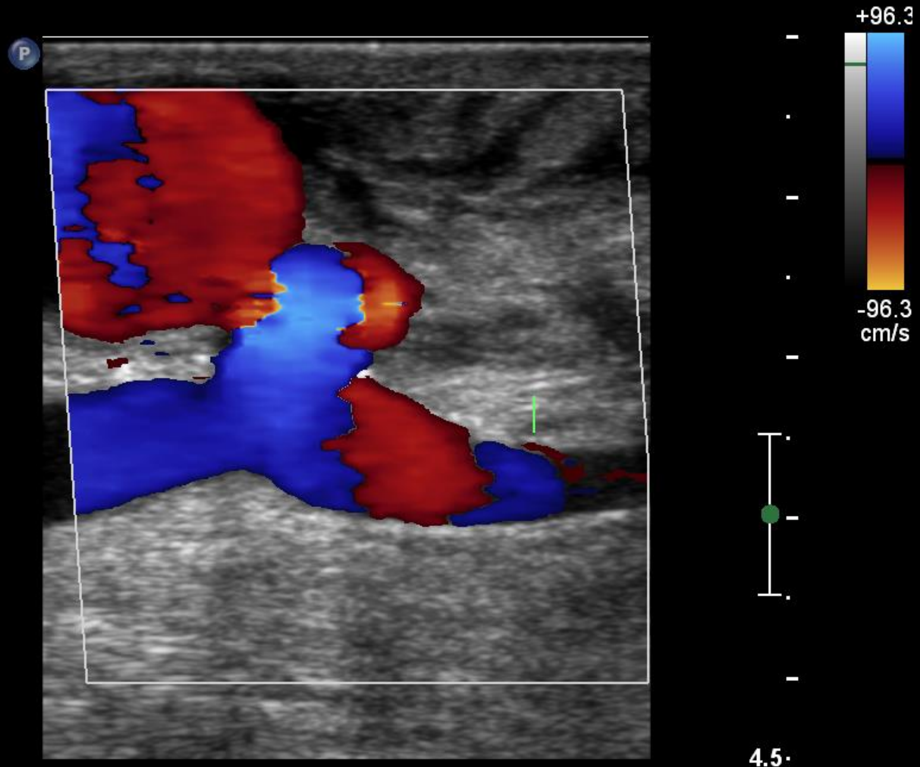

LT BR ARTERY /CEPH FISTULA

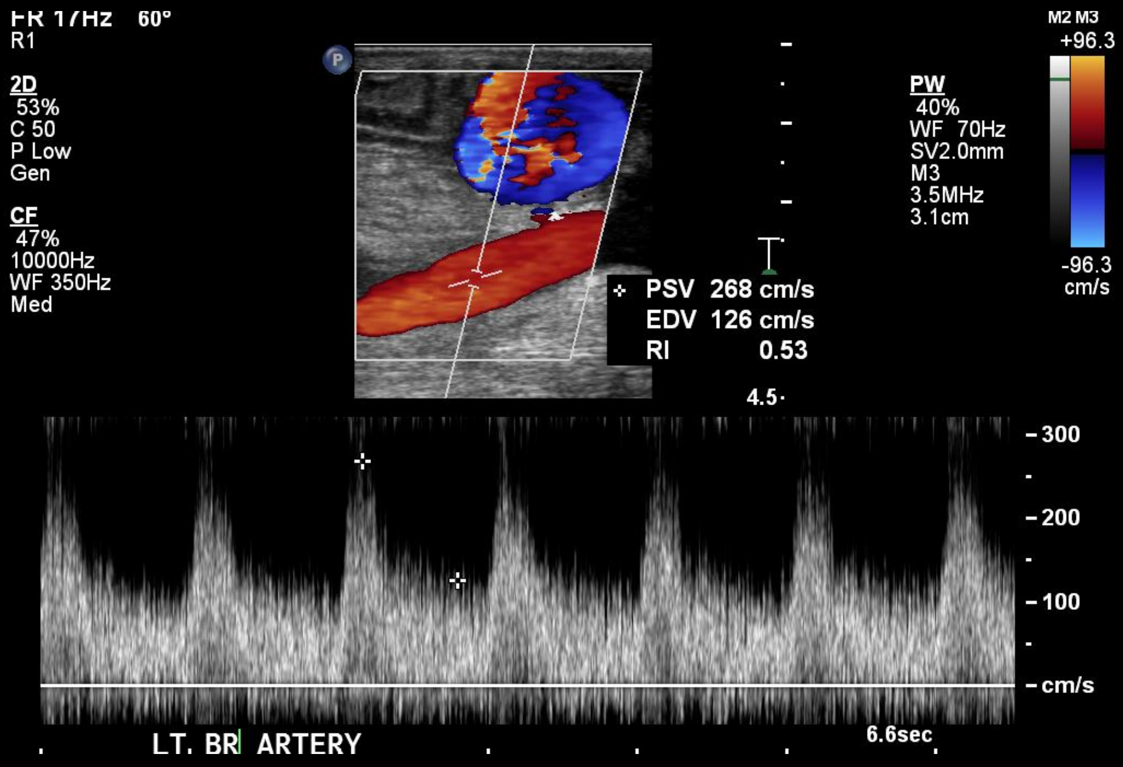
FR 17Hz 60°
R1
2D
53%
C 50
P Low
Gen
CF
47%
10000Hz
WF 350Hz
Med
PW
40%
WF 70Hz
SV2.0mm
M3
3.5MHz
3.1cm
M2 M3
+96.3
-96.3
cm/s
PSV 268 cm/s
EDV 126 cm/s
RI 0.53
4.5
300
200
100
cm/s
LT. BR ARTERY
6.6sec

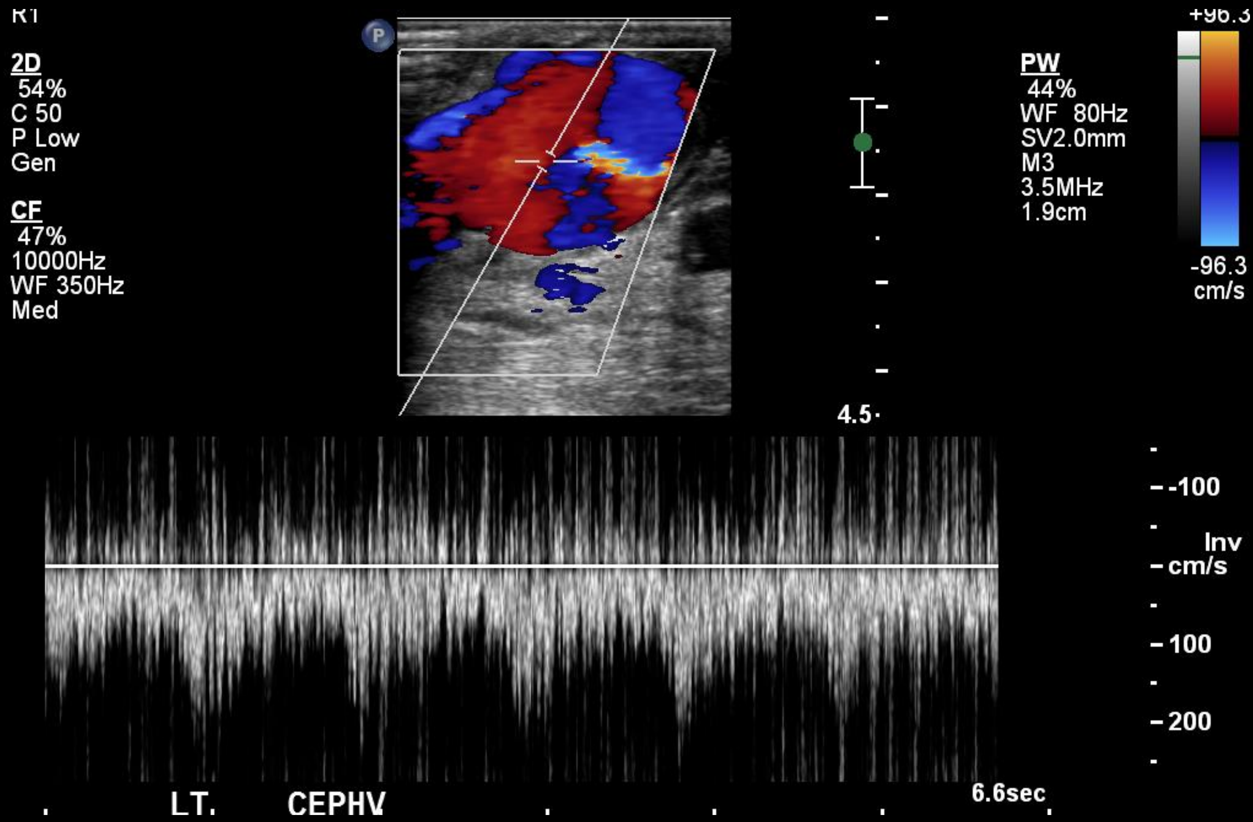
R1
2D
54%
C 50
P Low
Gen
CF
47%
10000Hz
WF 350Hz
Med
PW
44%
WF 80Hz
SV2.0mm
M3
3.5MHz
1.9cm
+96.3
-96.3
cm/s
4.5
-100
Inv
cm/s
100
200
LT. CEPHV
6.6sec

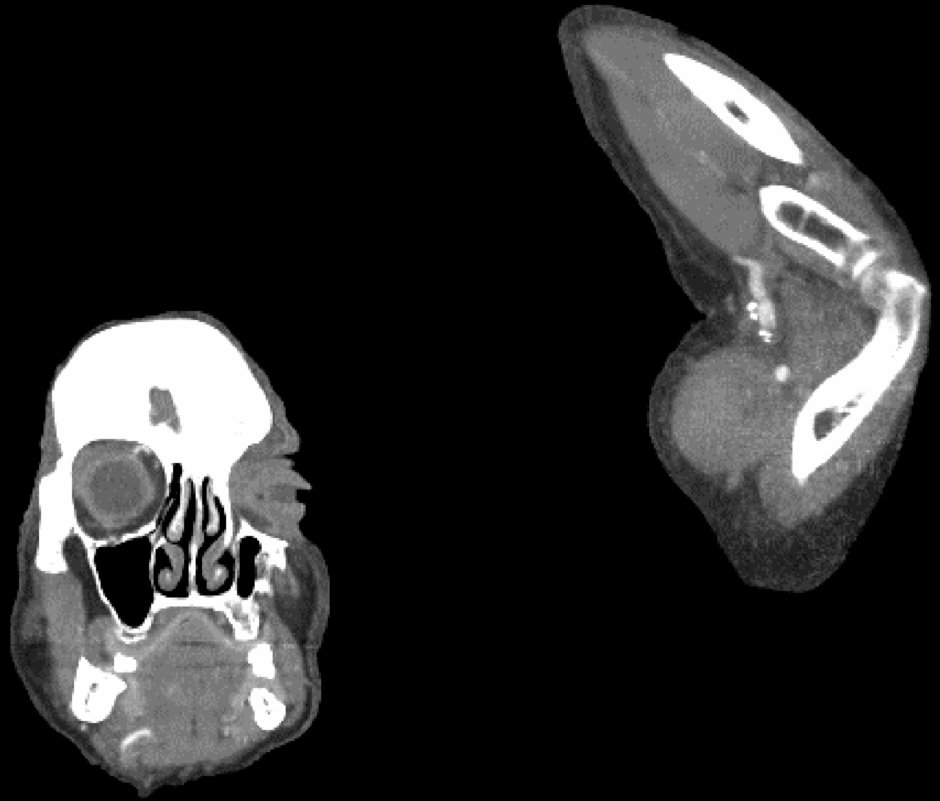

Hematoma or Pseudoaneurysm?

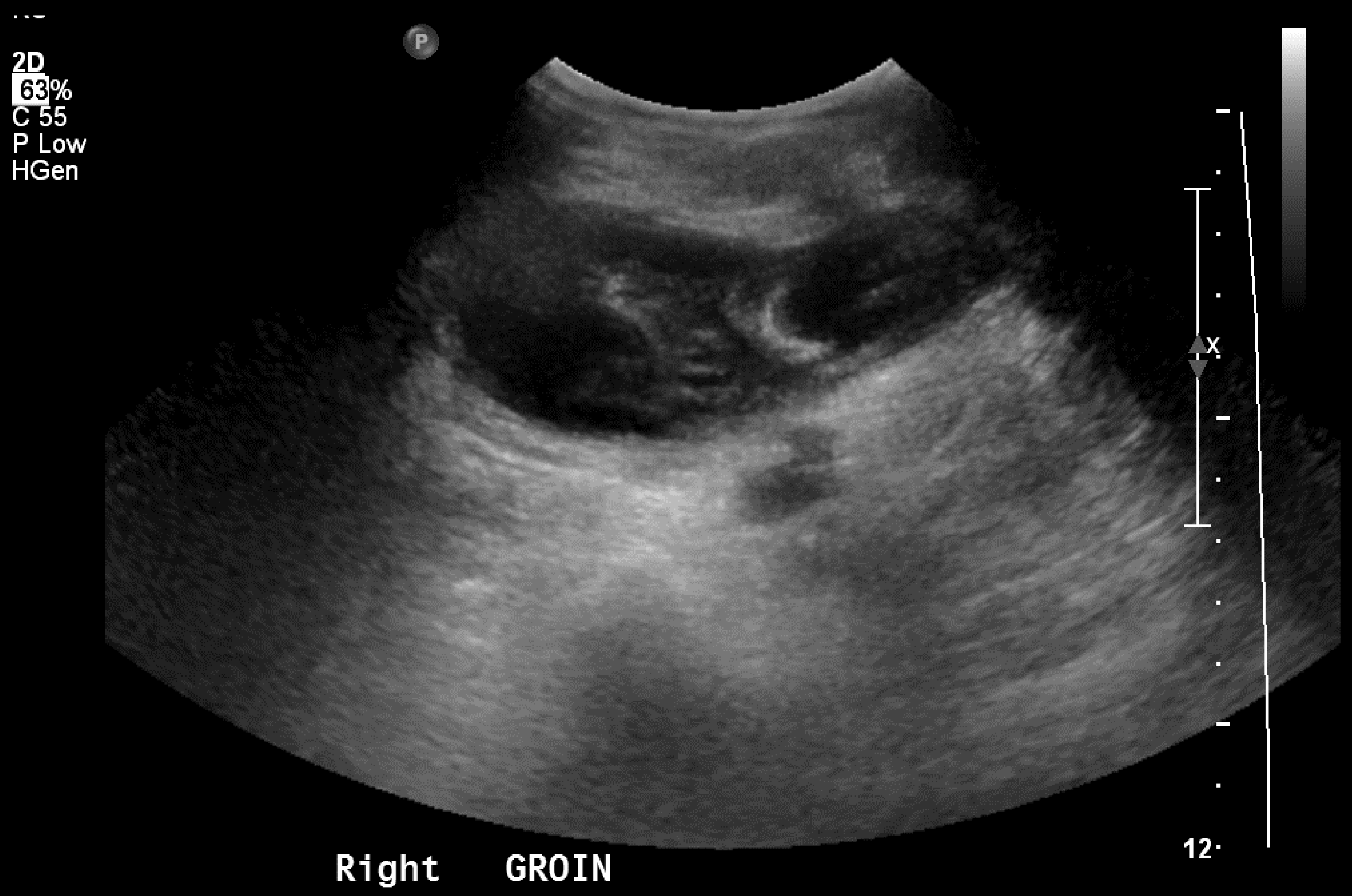

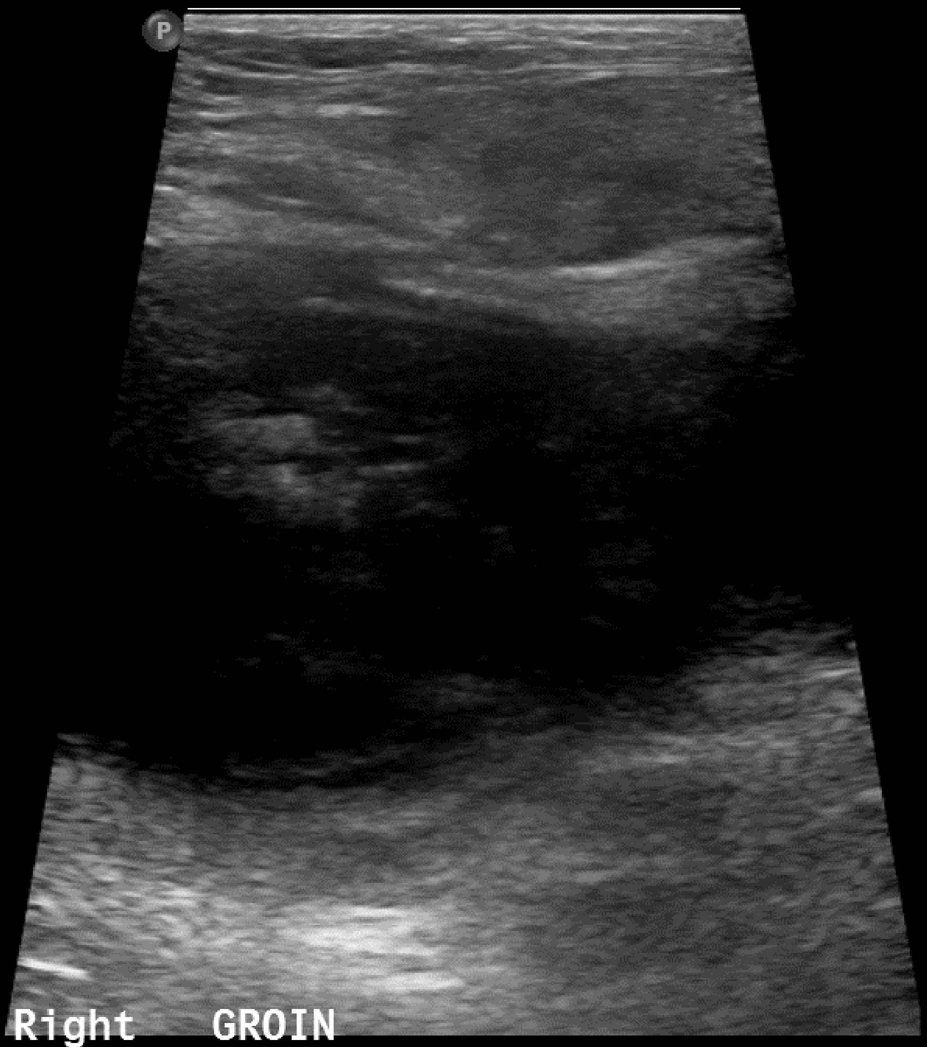

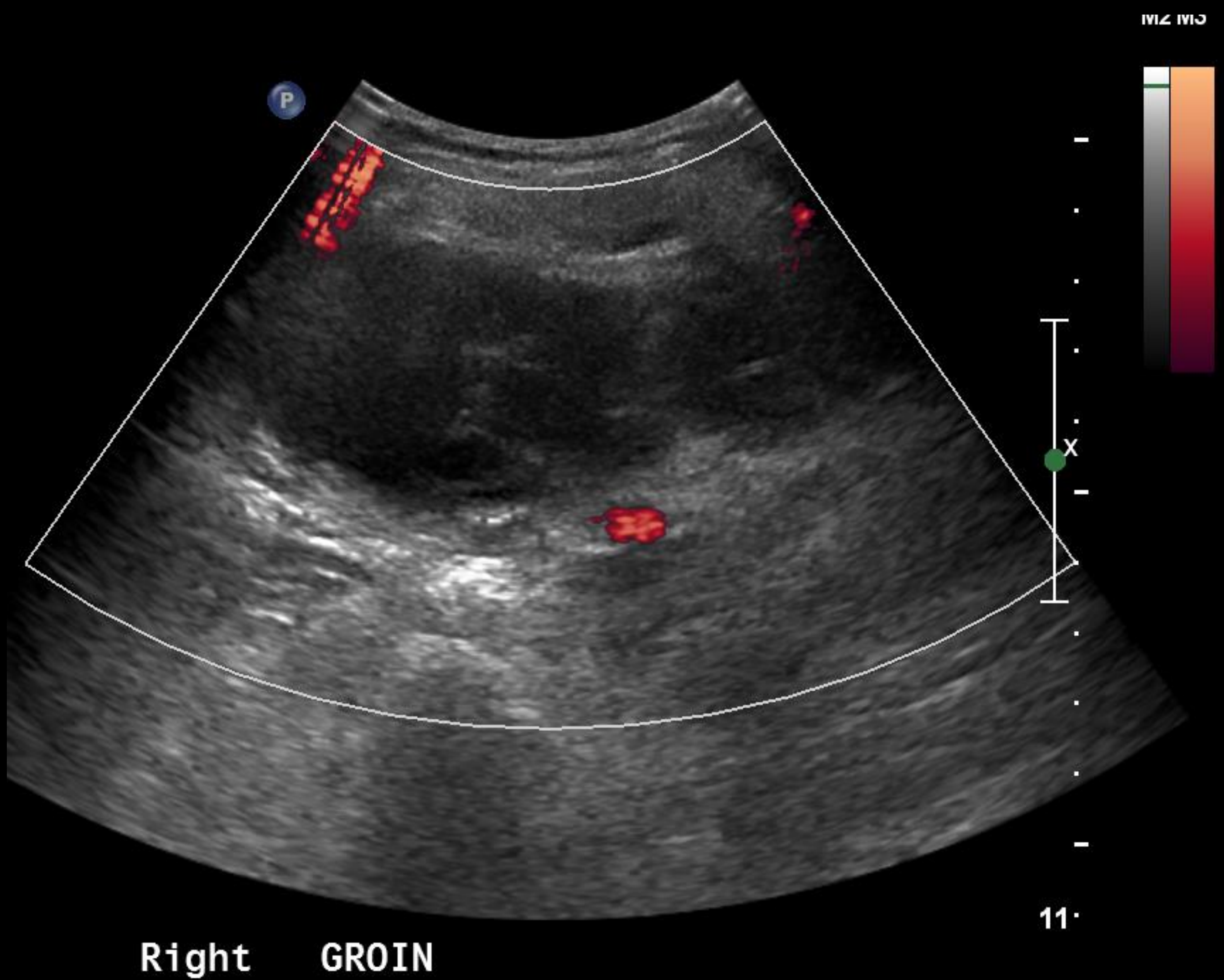

Hematoma*

Hematoma

- Complex fluid collection
- Mixed echogenicity
  - Acute and subacute → hyperechoic
  - Hyperacute → anechoic components, fluid-fluid levels
  - Chronic, liquefying → can also be ‘near anechoic’
- No internal flow (unless actively bleeding)

Edema

Subcutaneous Edema

- Linear areas of fluid within subcutaneous fat
- Cobblestone or striated appearance

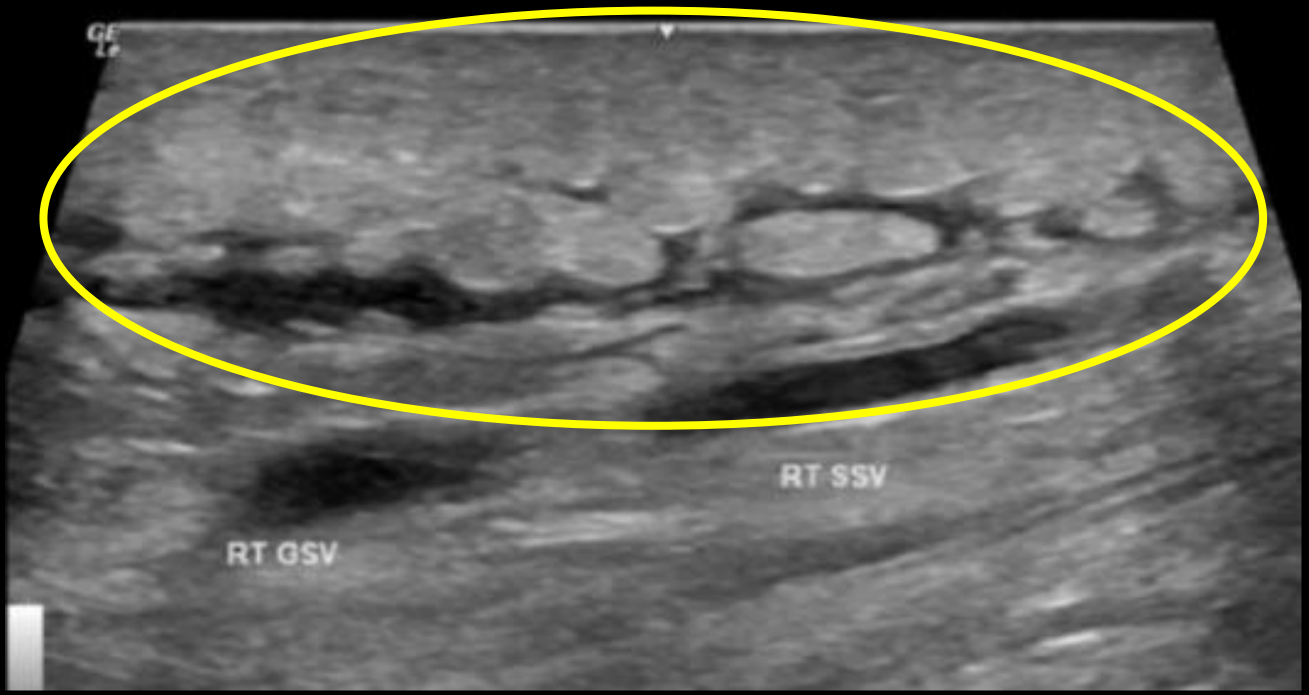

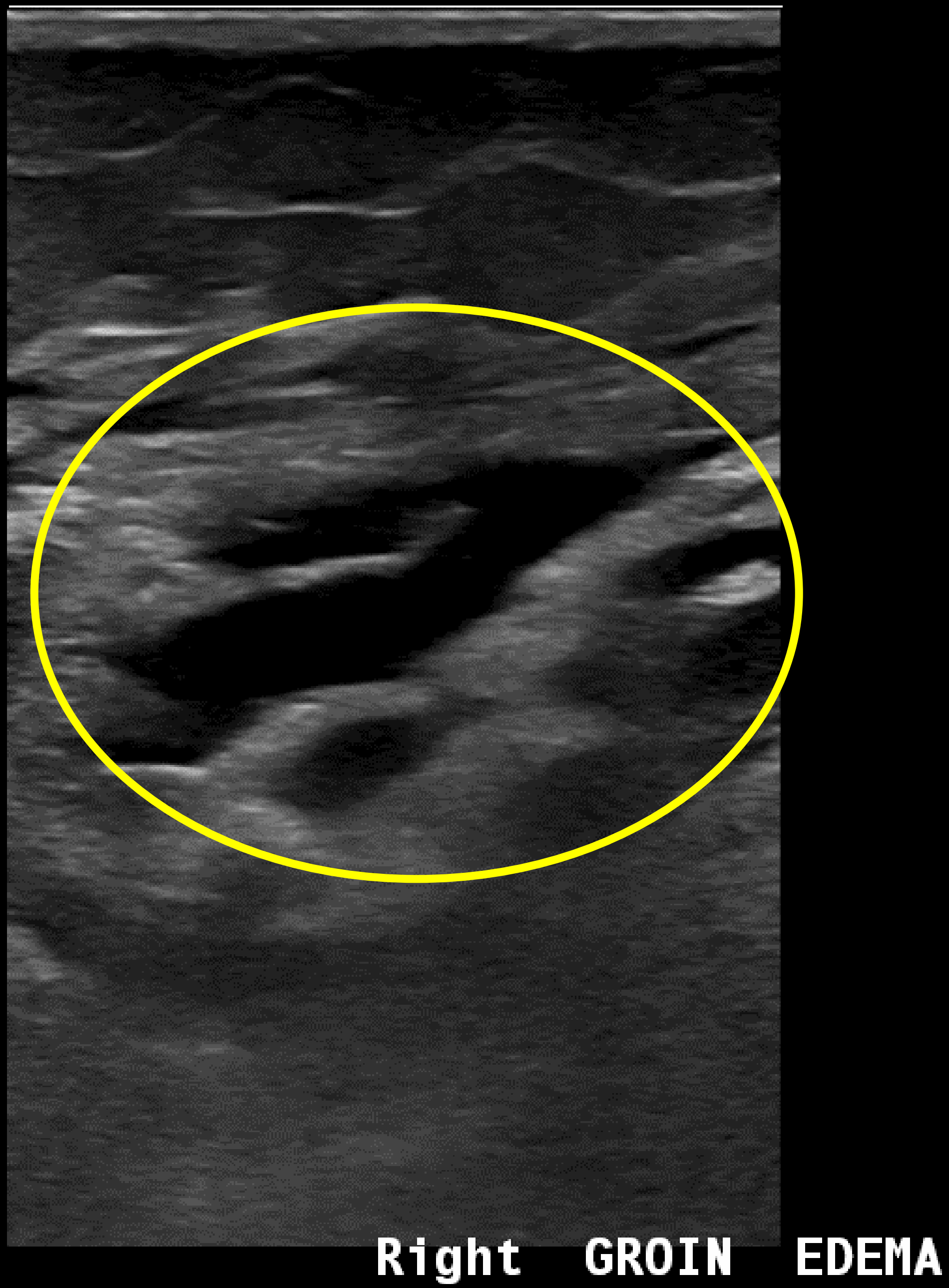

Tricky Case

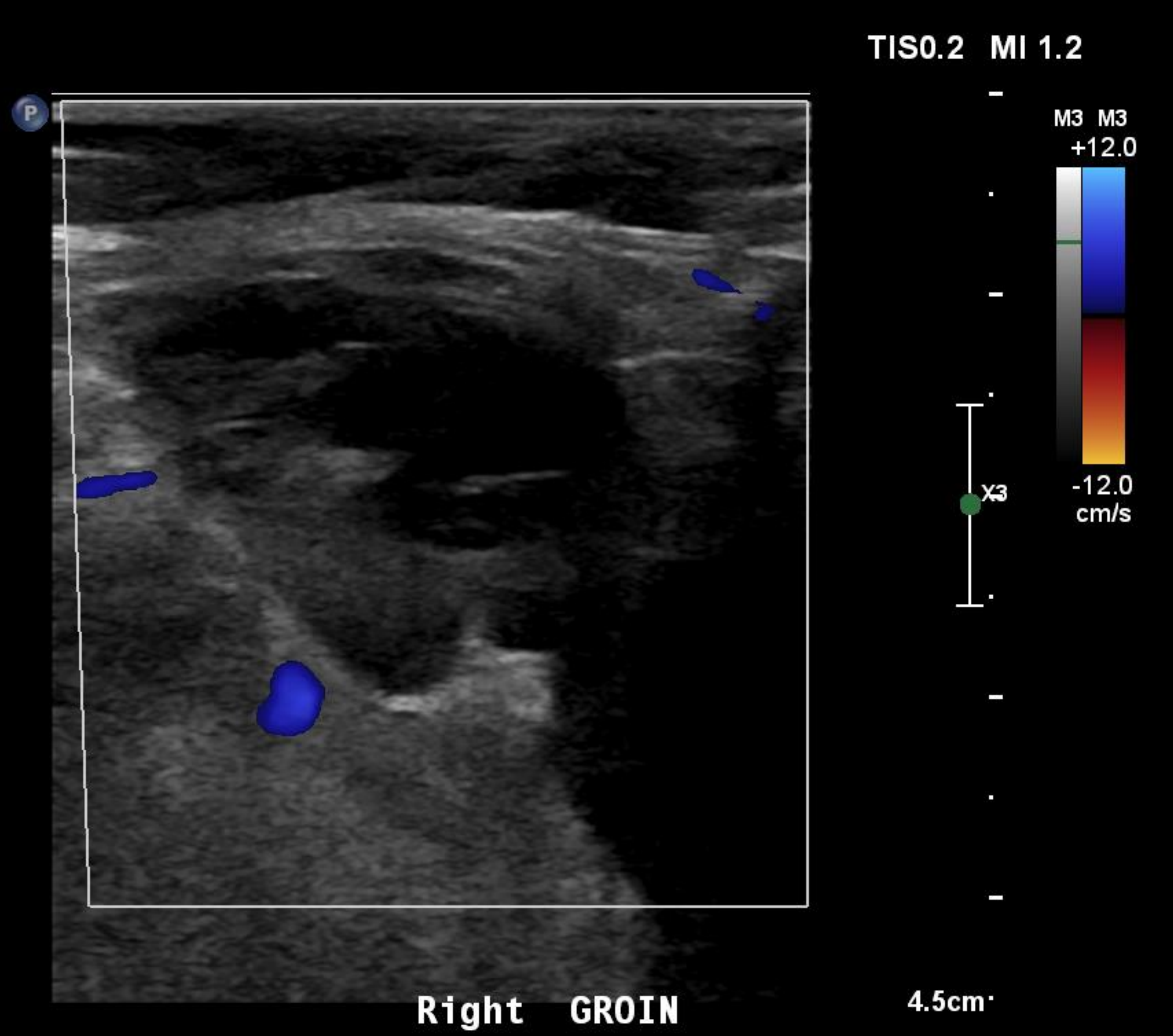
TIS0.2 MI 1.2
M3 M3
+12.0
-12.0
cm/s
X3
4.5cm
Right GROIN

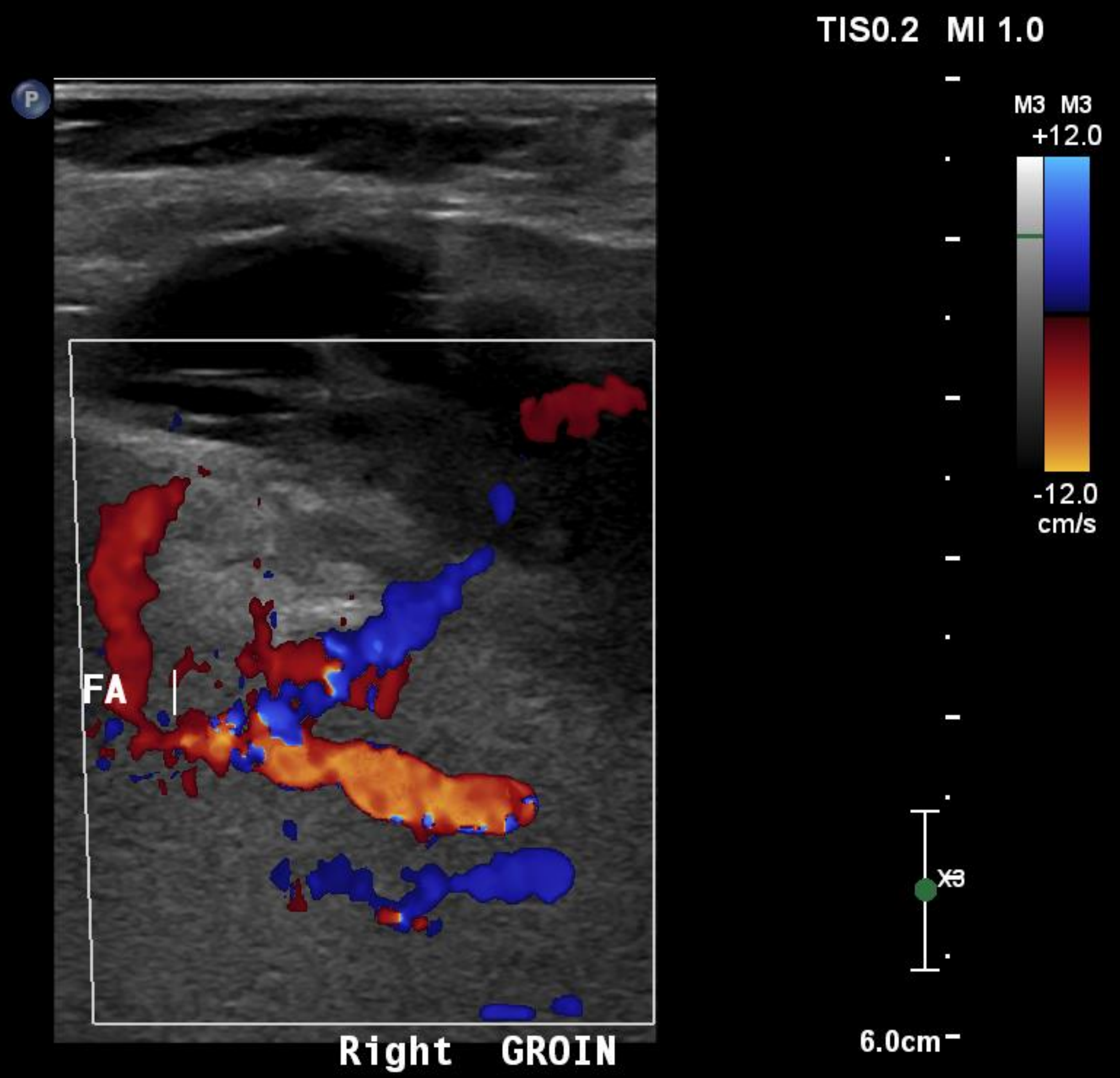
TIS0.2 MI 1.0
M3 M3
+12.0
-12.0
cm/s
FA
X3
6.0cm
Right GROIN

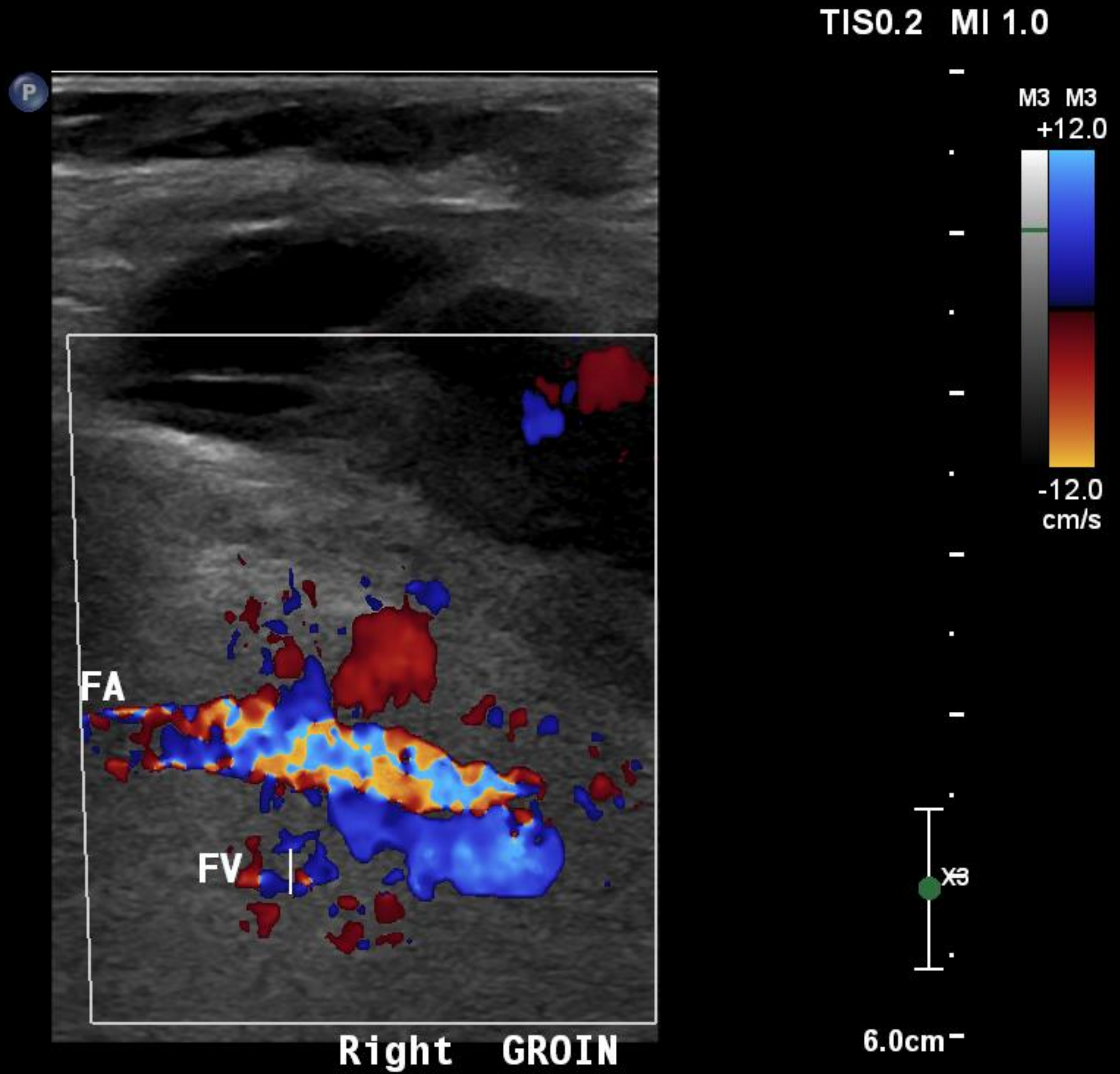
TIS0.2 MI 1.0
M3 M3
+12.0
-12.0
cm/s
FA
FV
Right GROIN
6.0cm

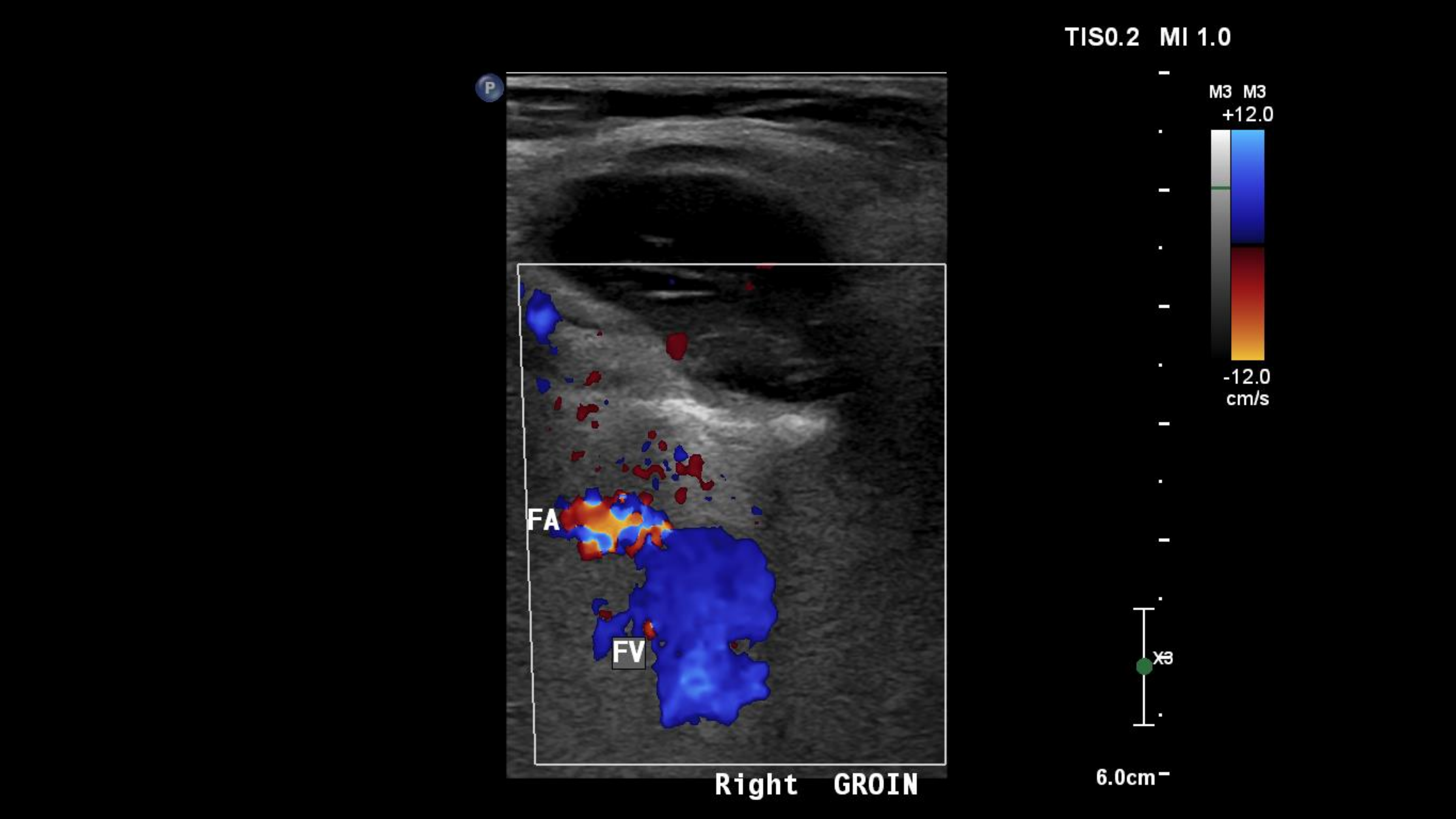
TIS0.2 MI 1.0
M3 M3
+12.0
-12.0
cm/s
FA
FV
X3
6.0cm
Right GROIN

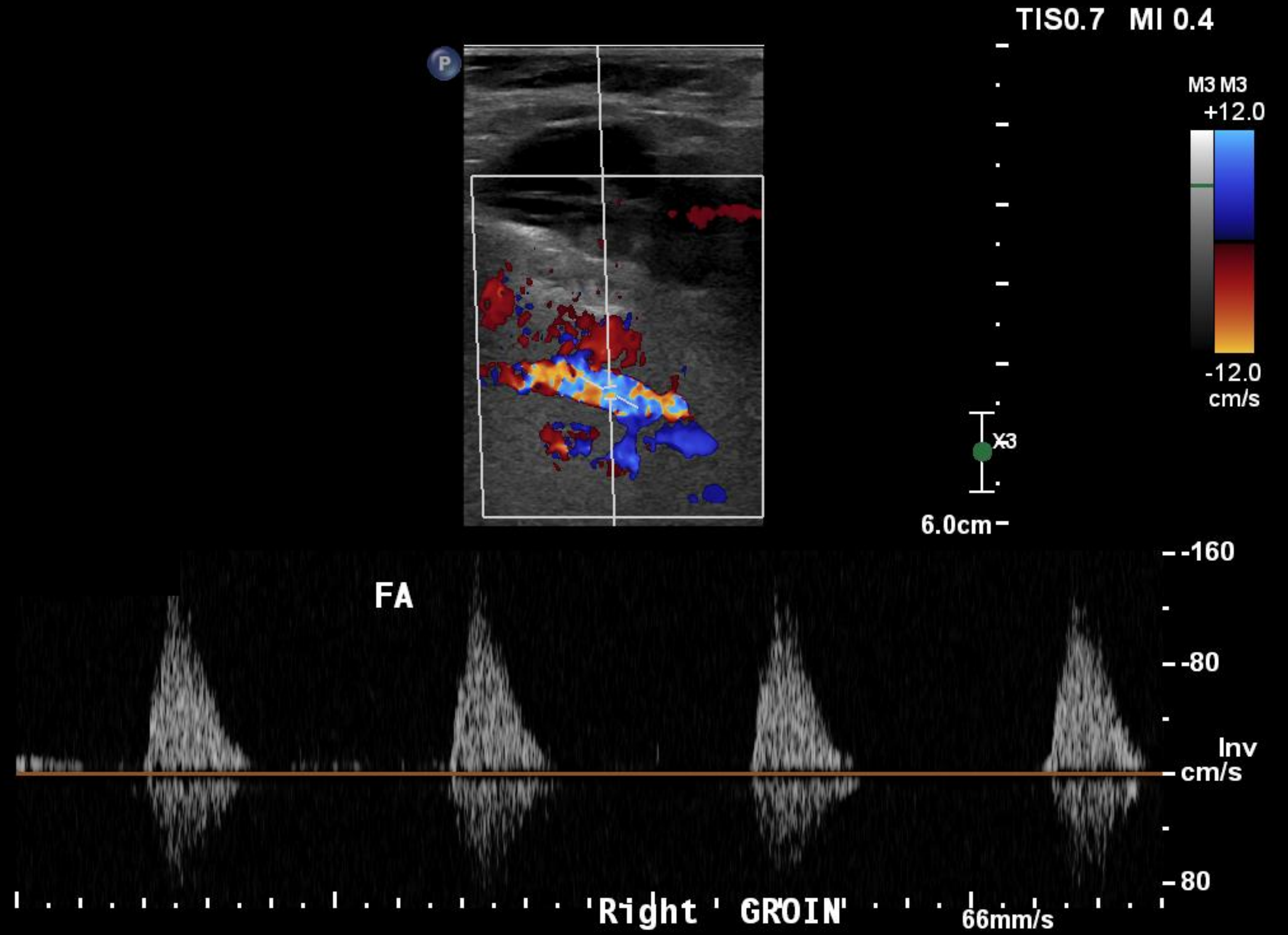

TIS0.7 MI 0.4
M3 M3
+12.0
-12.0
cm/s
X3
6.0cm
-160
FA
-80
Inv
cm/s
80
Right GROIN
66mm/s

Is this:

- A hematoma?
- A pseudoaneurysm?
- A partially thrombosed pseudoaneurysm?
- Other?

Hint:

The collection is getting bigger as you scan!!

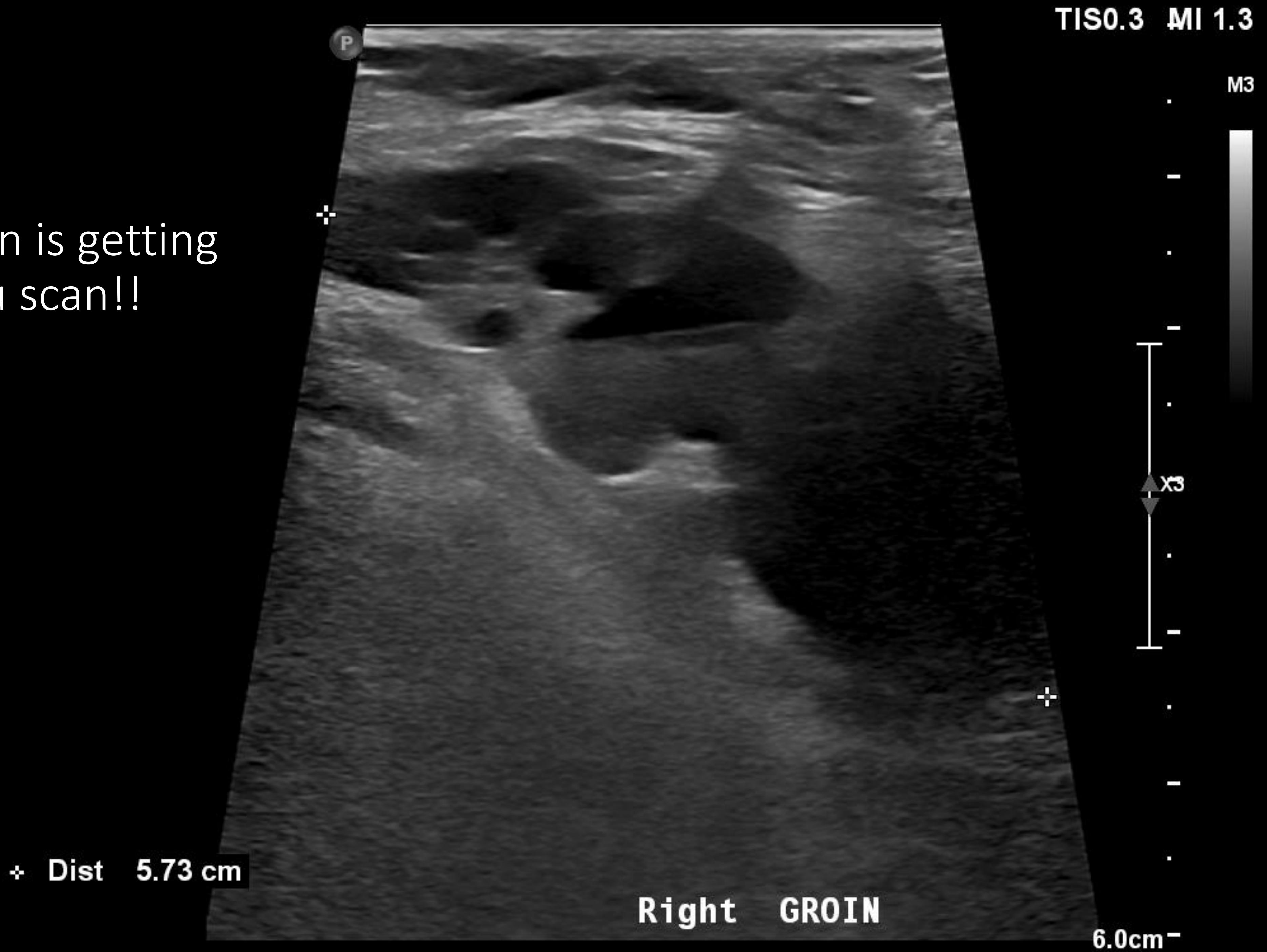

Answer:

Hematoma with active bleeding

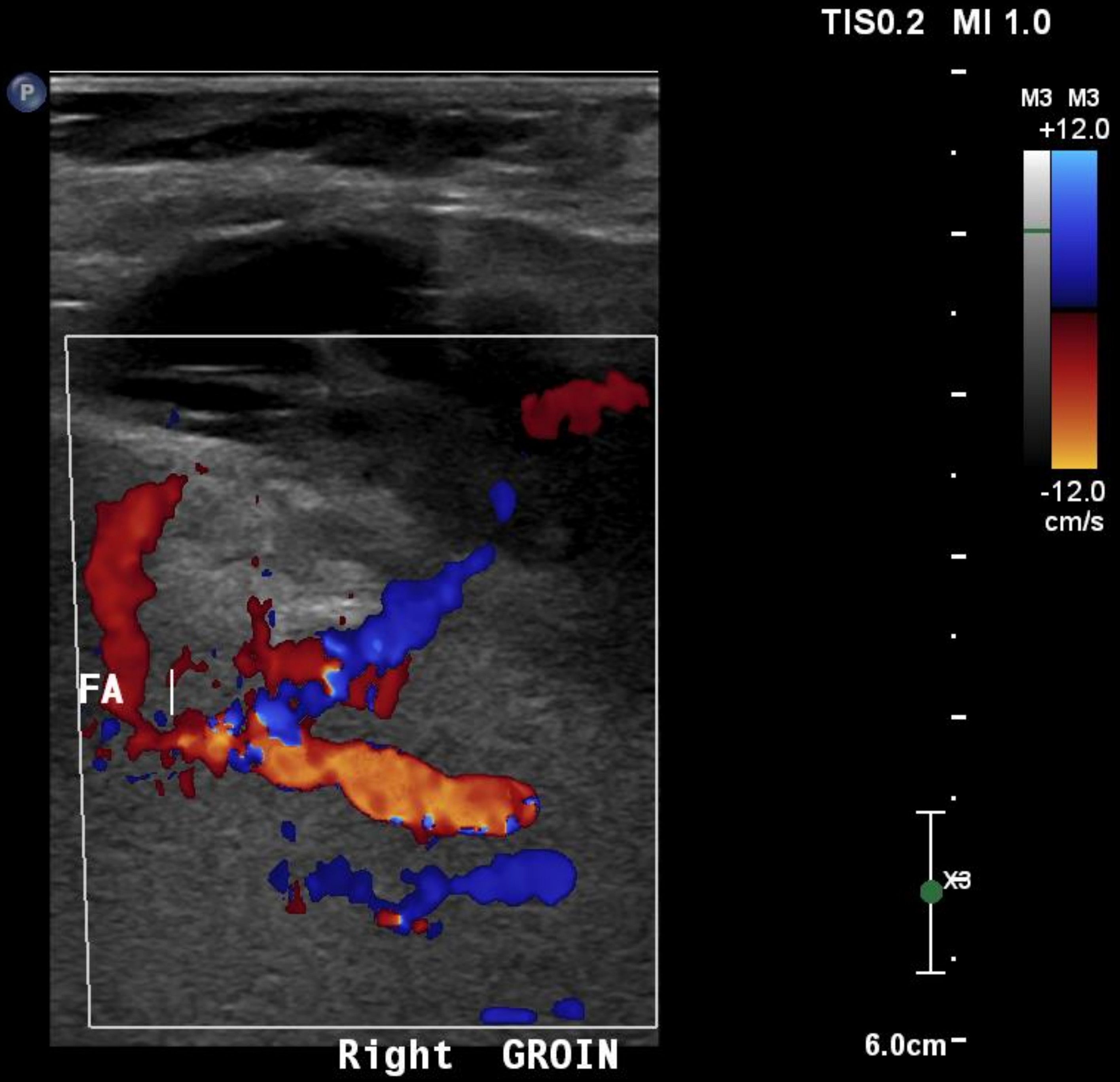

Reality

Complications can be found in combination together

(eg. pseudoaneurysm + hematoma)

(pseudoaneurysm + hematoma + active rupture/bleeding)

Pseudoaneurysm AND AV-fistula

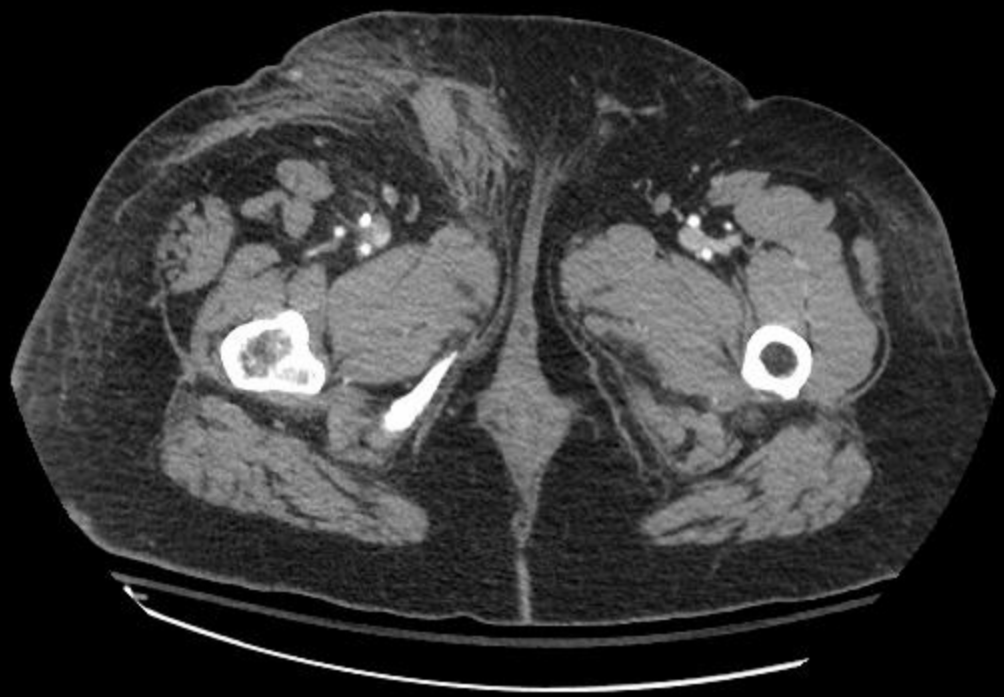

What's going on here?

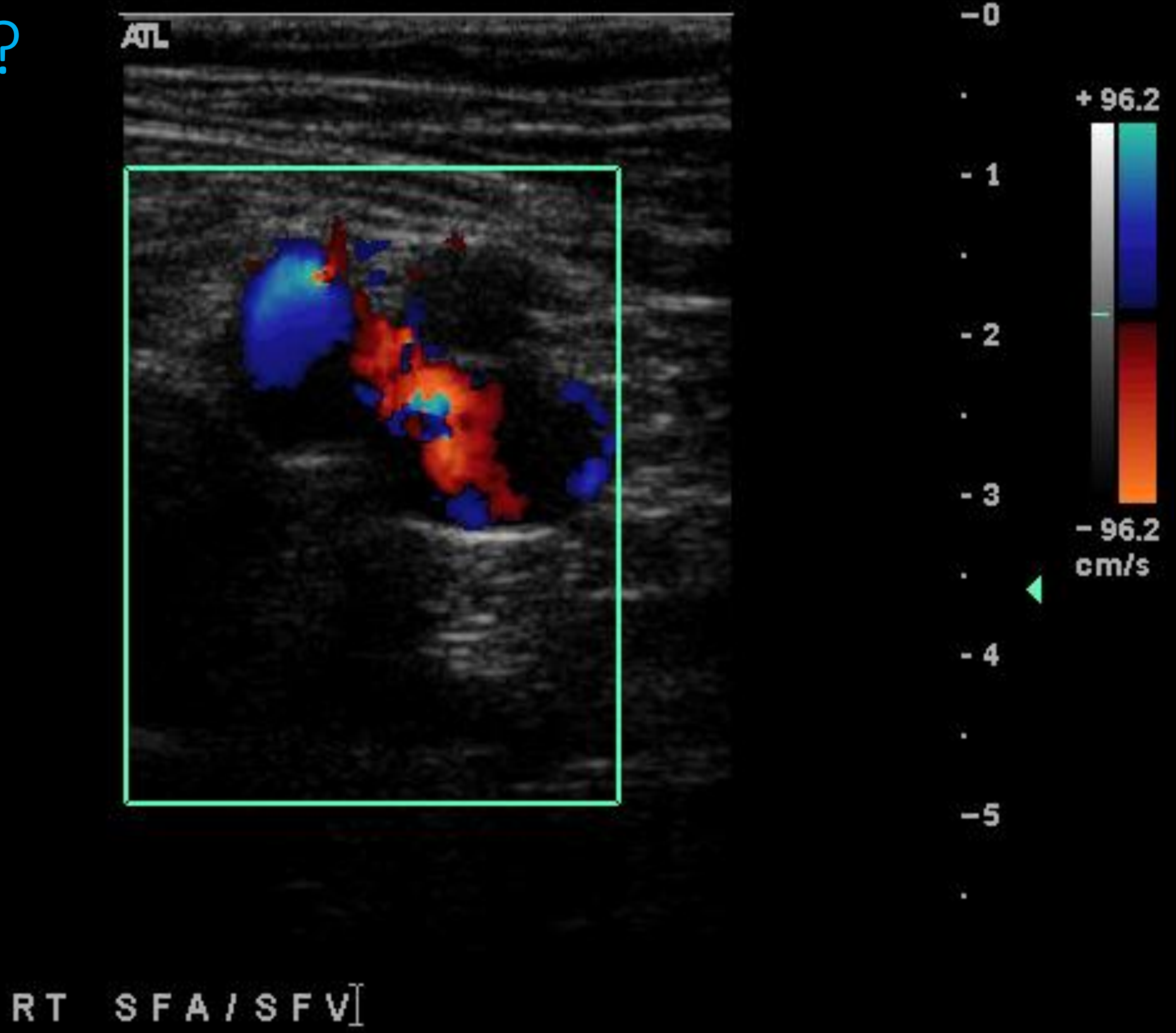

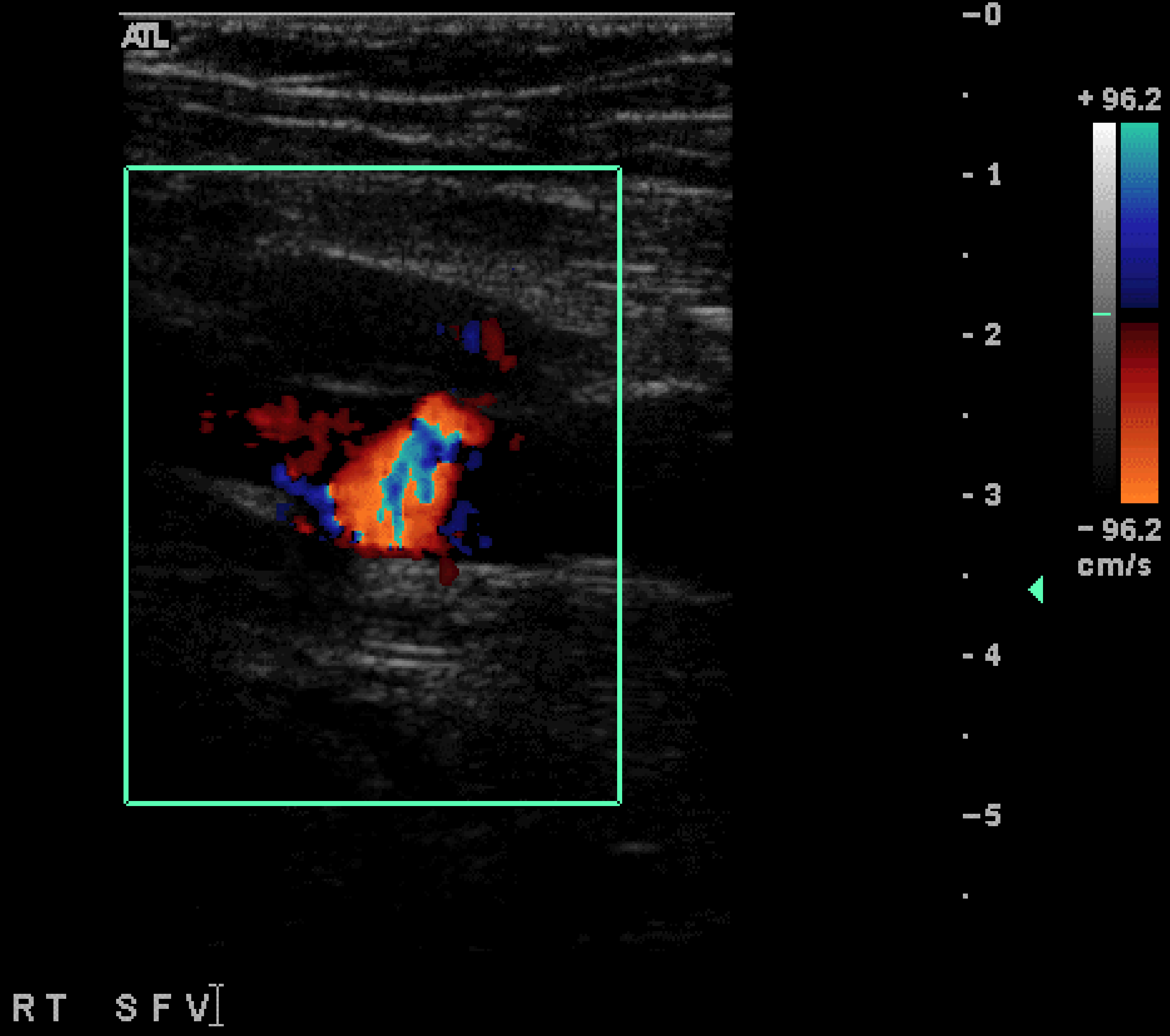
ATL
0
1
2
3
4
5
+ 96.2
− 96.2
cm/s
RT SFV

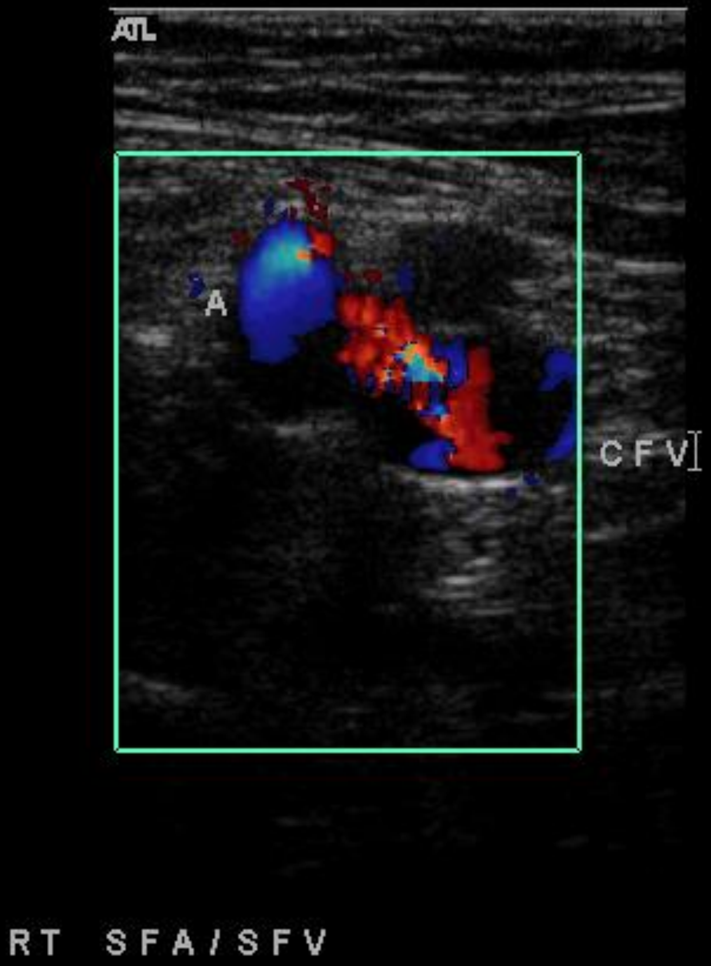

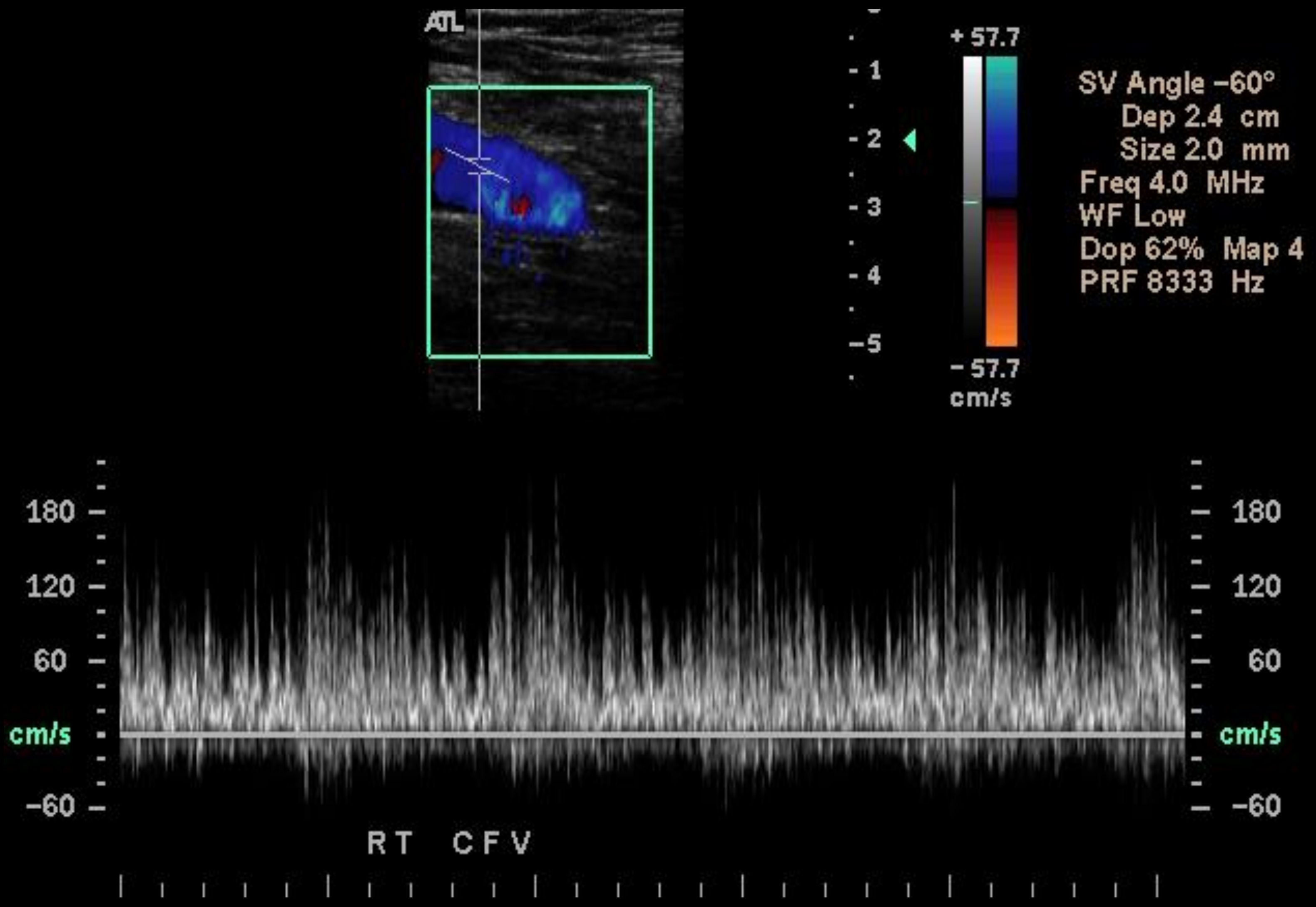

Is this a normal waveform? → Femoral vein

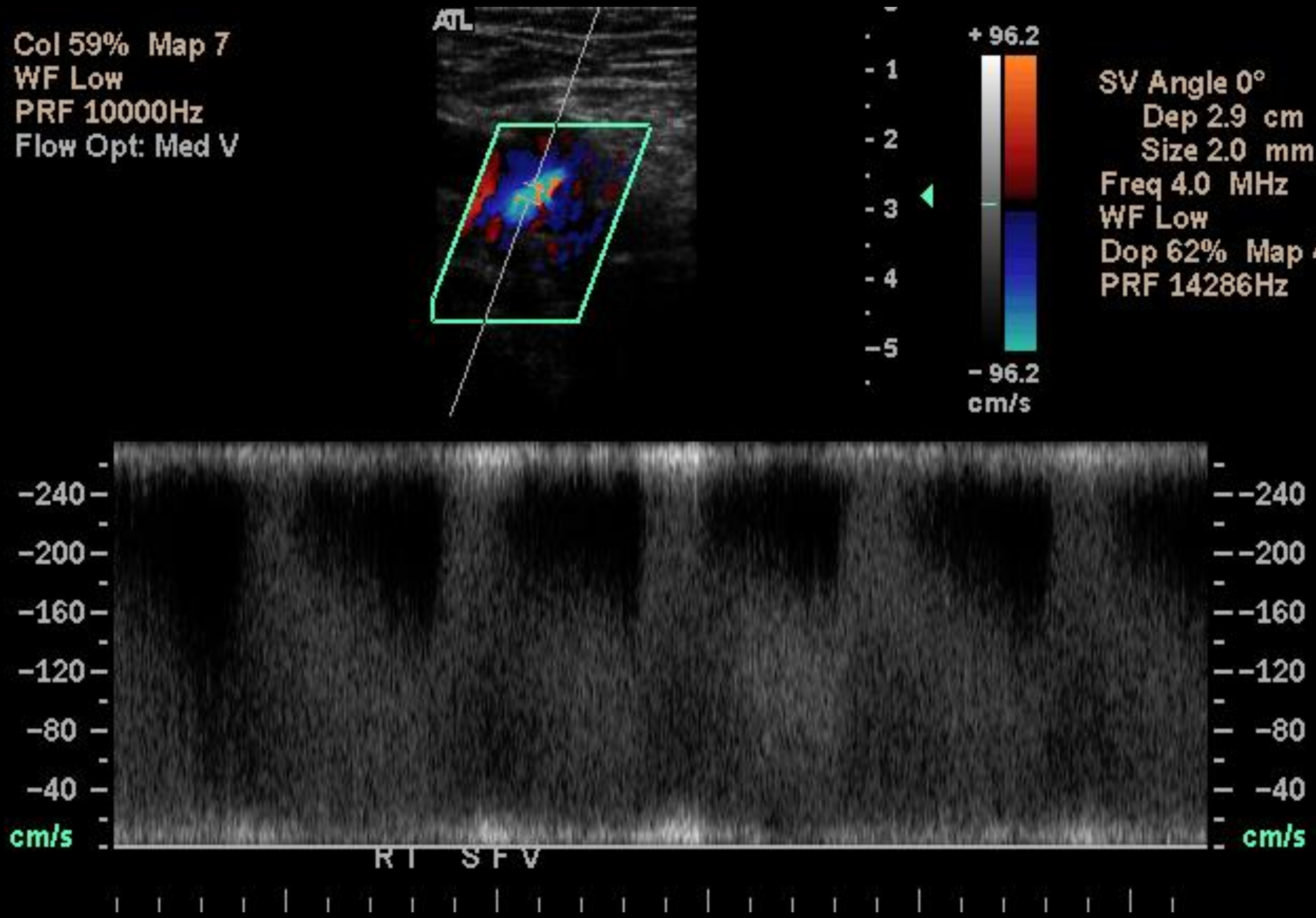

Area of 'aliasing' found between artery and vein

Arteriovenous Fistula

Arteriovenous Fistula (AVF)

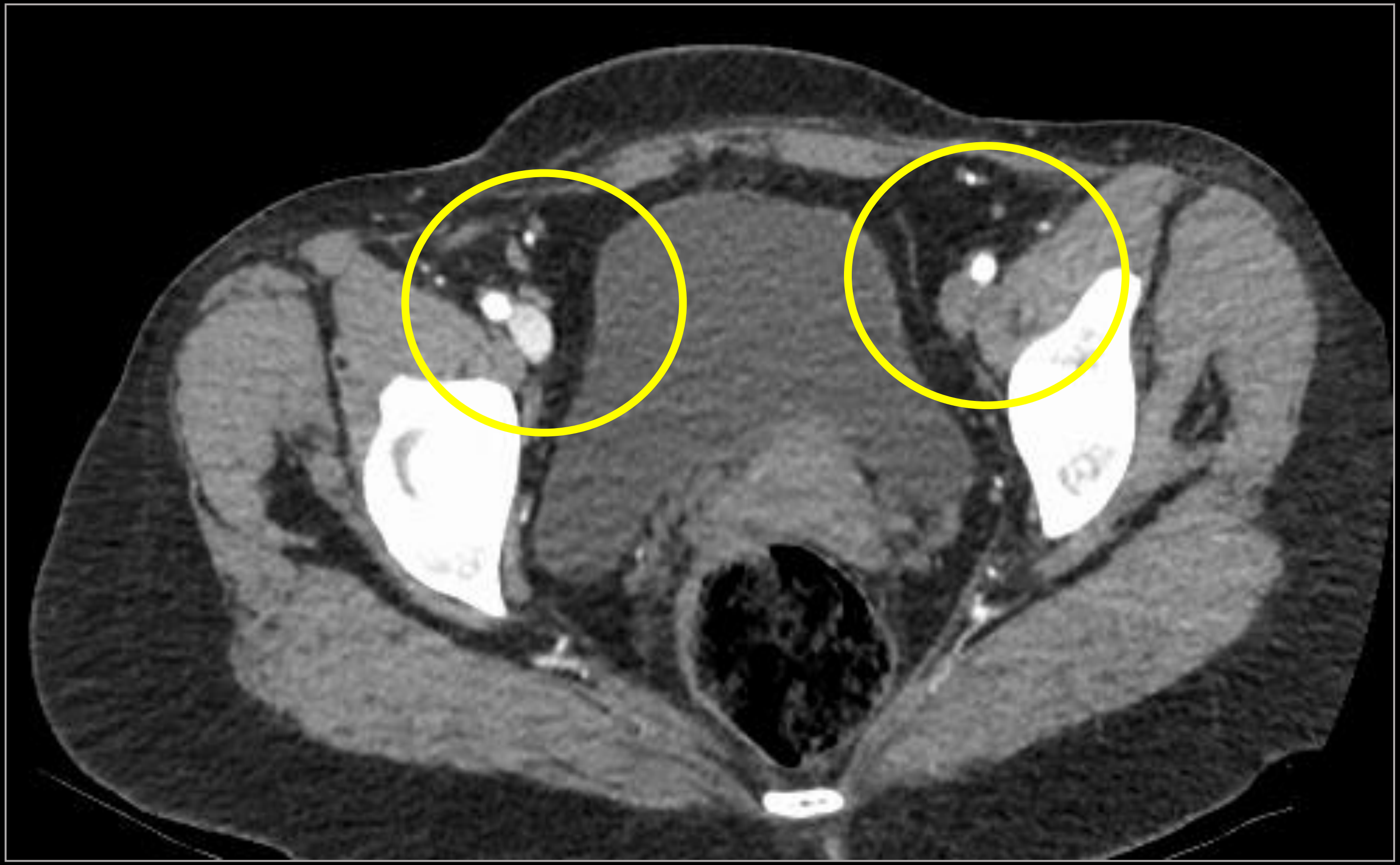

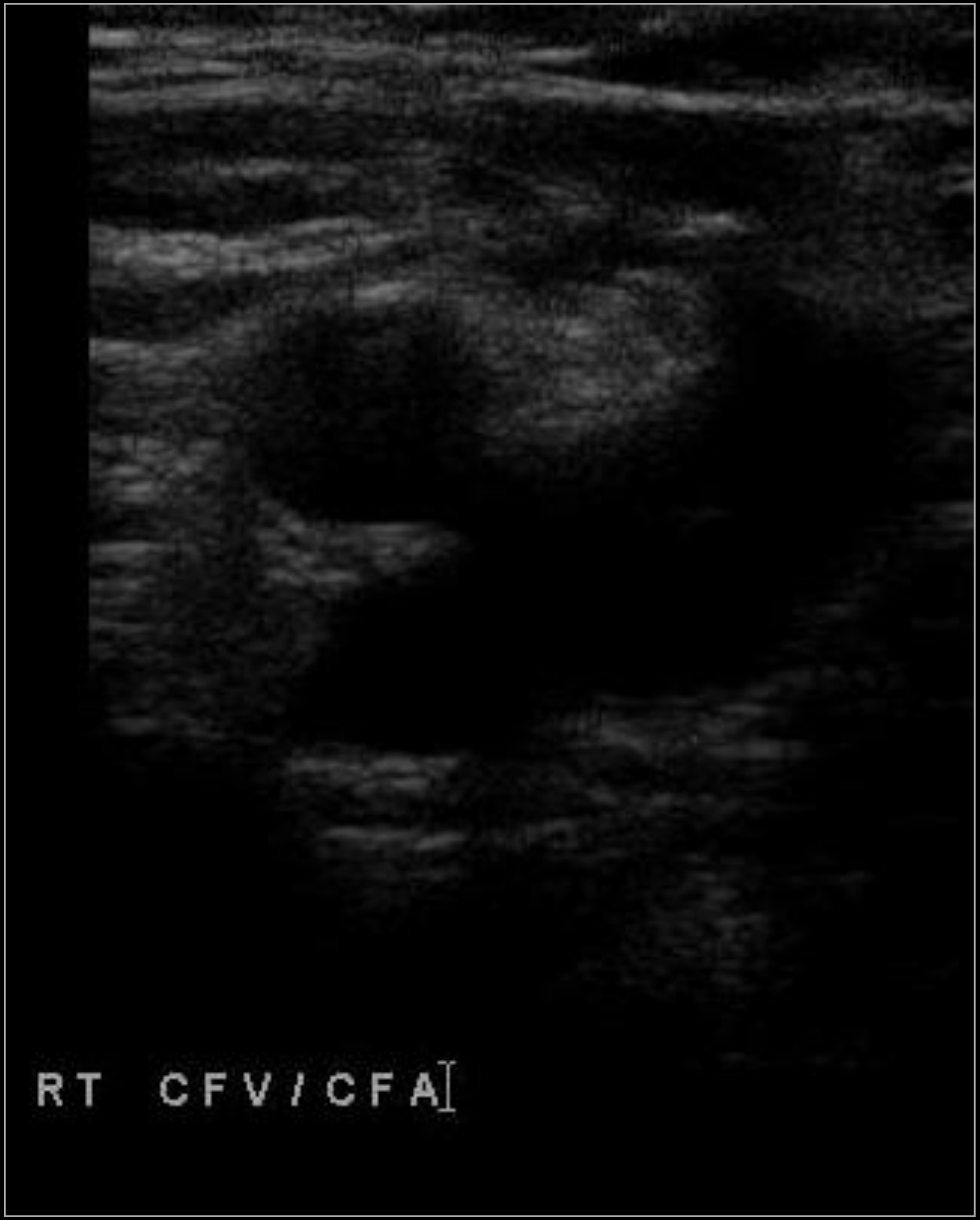
RT CFV/CFA

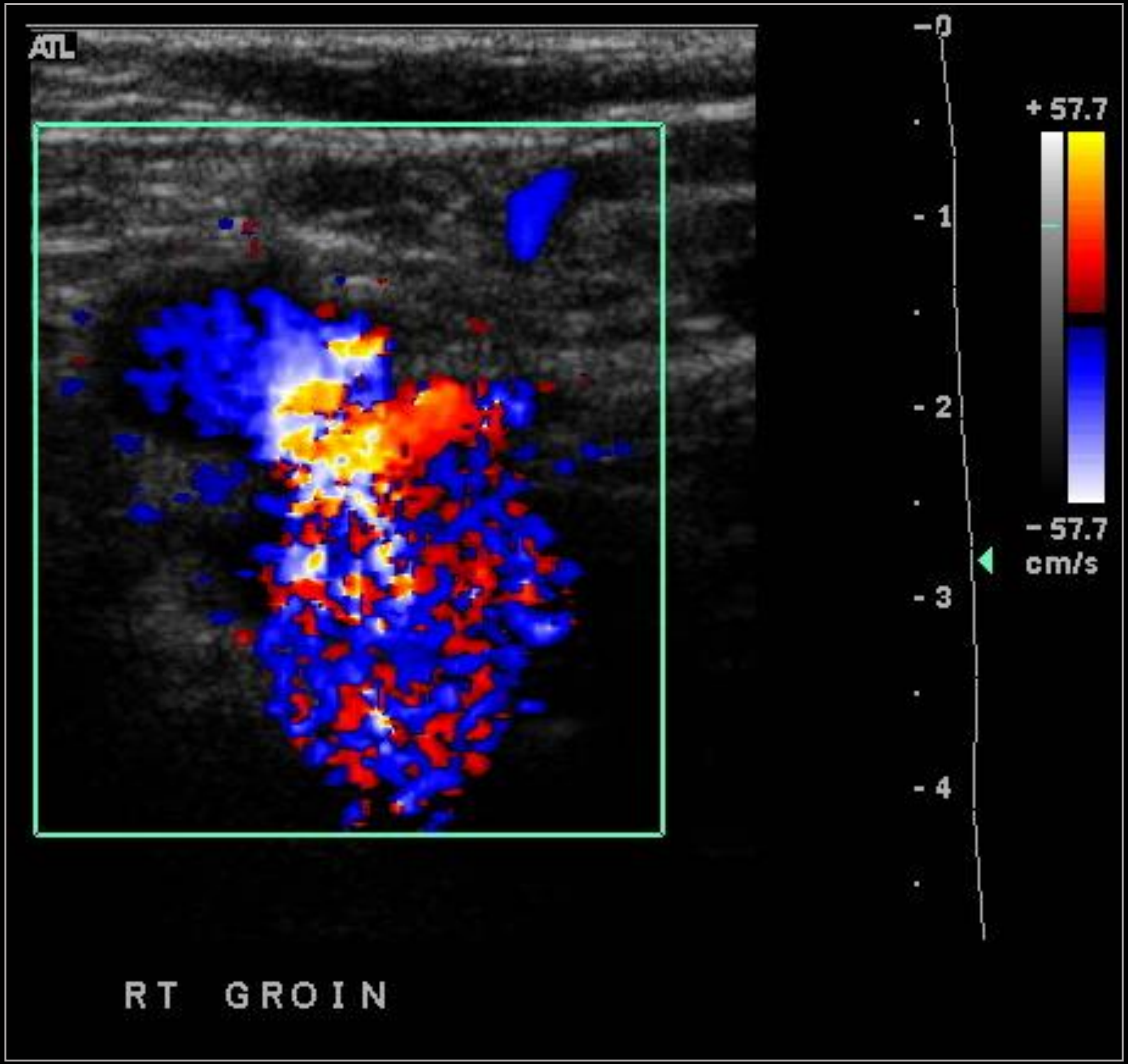
ATL
0
1
2
3
4
+ 57.7
- 57.7
cm/s
RT GROIN

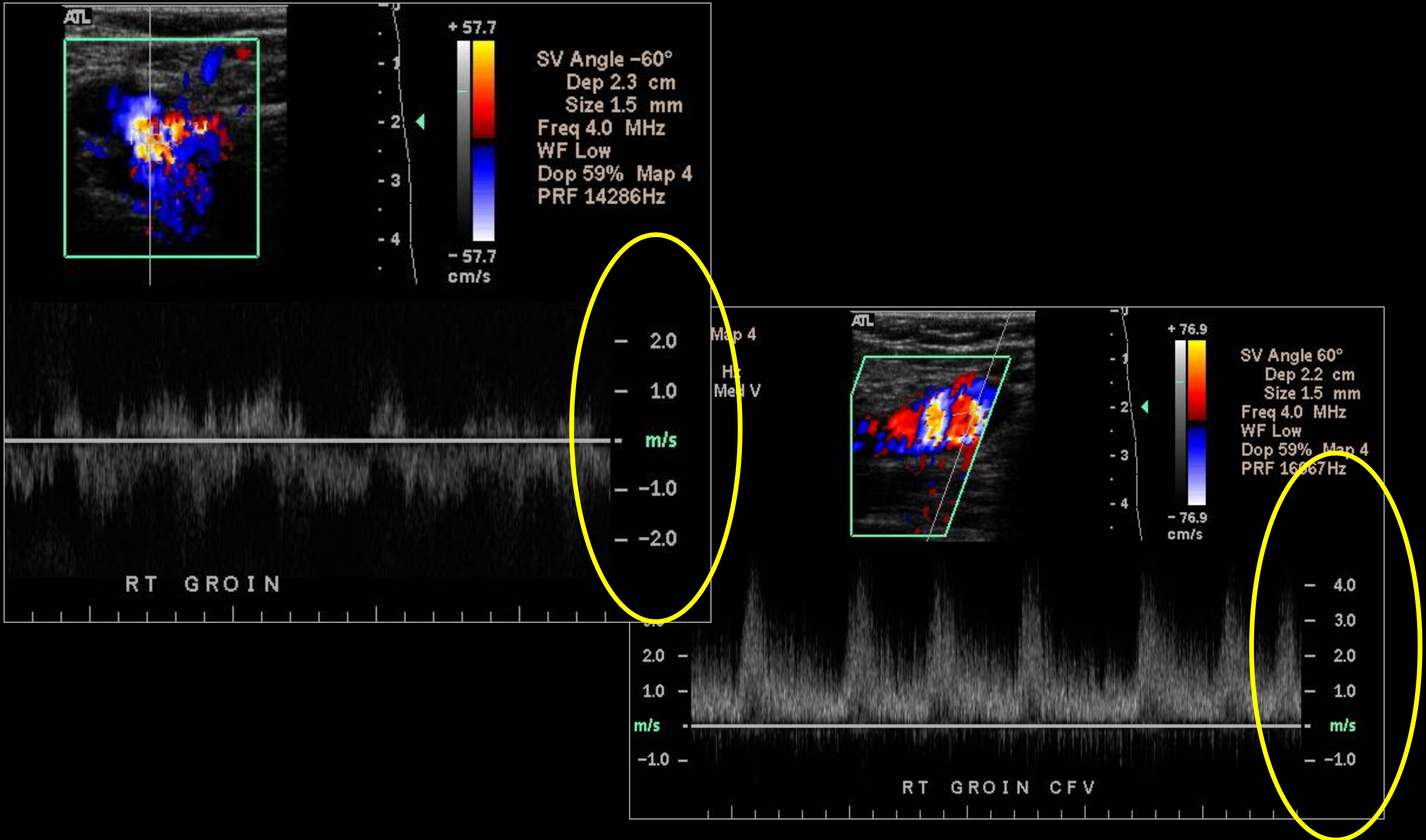
ATL
+ 57.7
SV Angle -60°
Dep 2.3 cm
Size 1.5 mm
Freq 4.0 MHz
WF Low
Dop 59% Map 4
PRF 14286Hz
- 57.7
cm/s
2.0
1.0
m/s
-1.0
-2.0
RT GROIN
ATL
+ 76.9
SV Angle 60°
Dep 2.2 cm
Size 1.5 mm
Freq 4.0 MHz
WF Low
- 76.9
cm/s
4.0
3.0
2.0
1.0
m/s
-1.0
RT GROIN CFV

Let’s look at a normal vein

A normal vein:

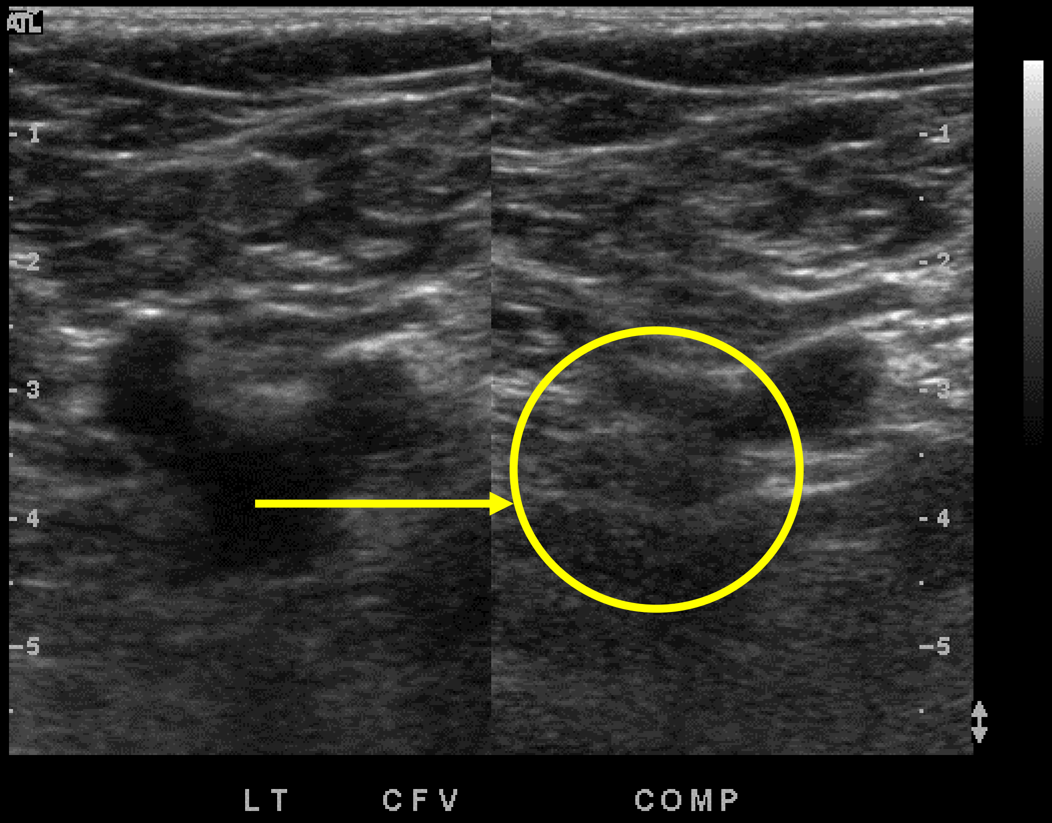

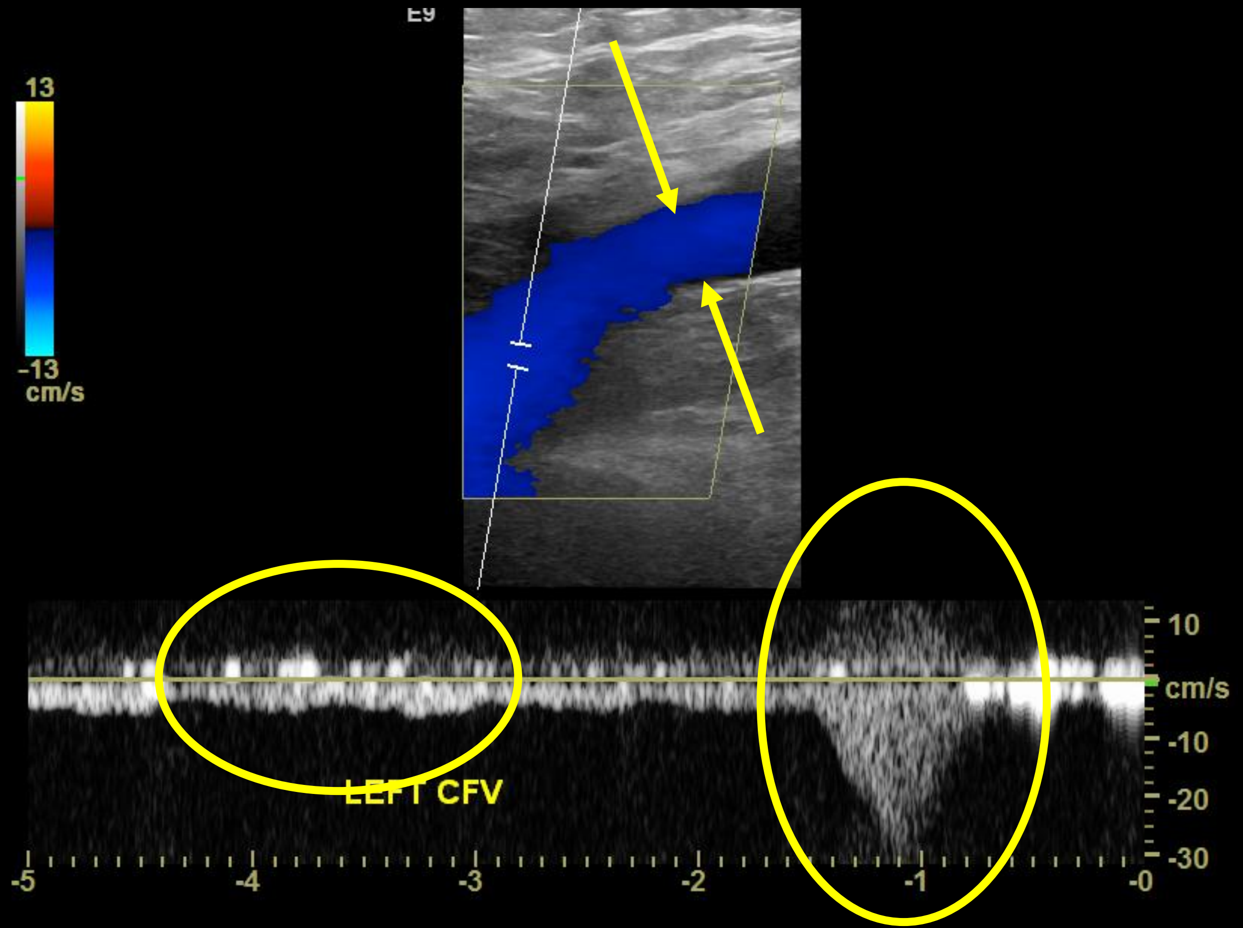

- Compresses
- Shows flow (fills uniformly on colour Doppler)
- Venous waveform (low velocity, phasicity – NOT pulsatile*)
- Augments

If pulsatility seen within a vein, consider:

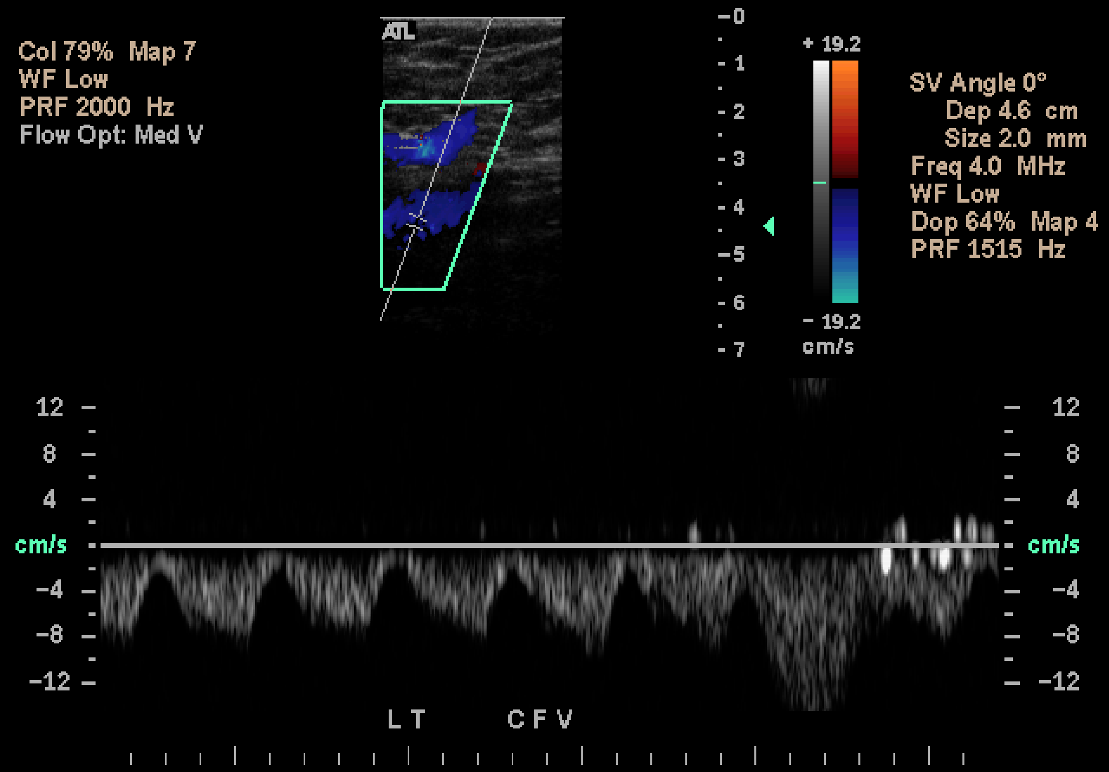

Right sided HF
(low velocity, gently “pulsatile”)

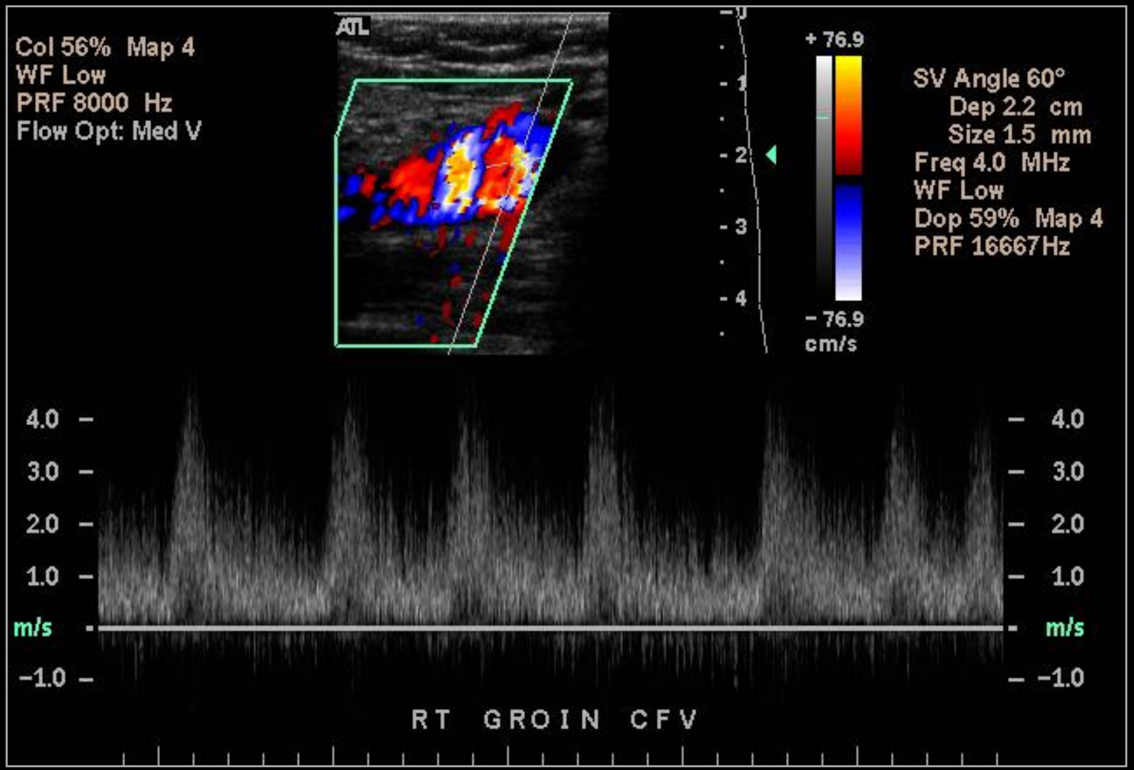

AVF
(high velocity, turbulent)

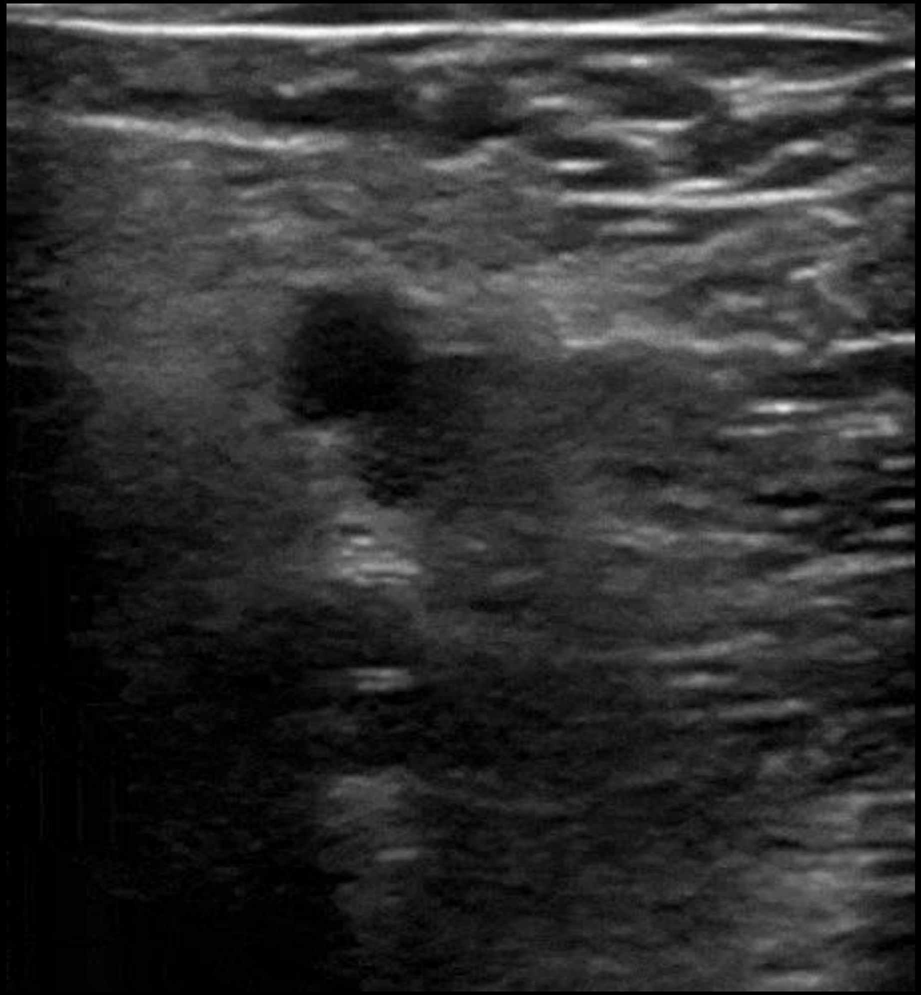

Diagnosis?

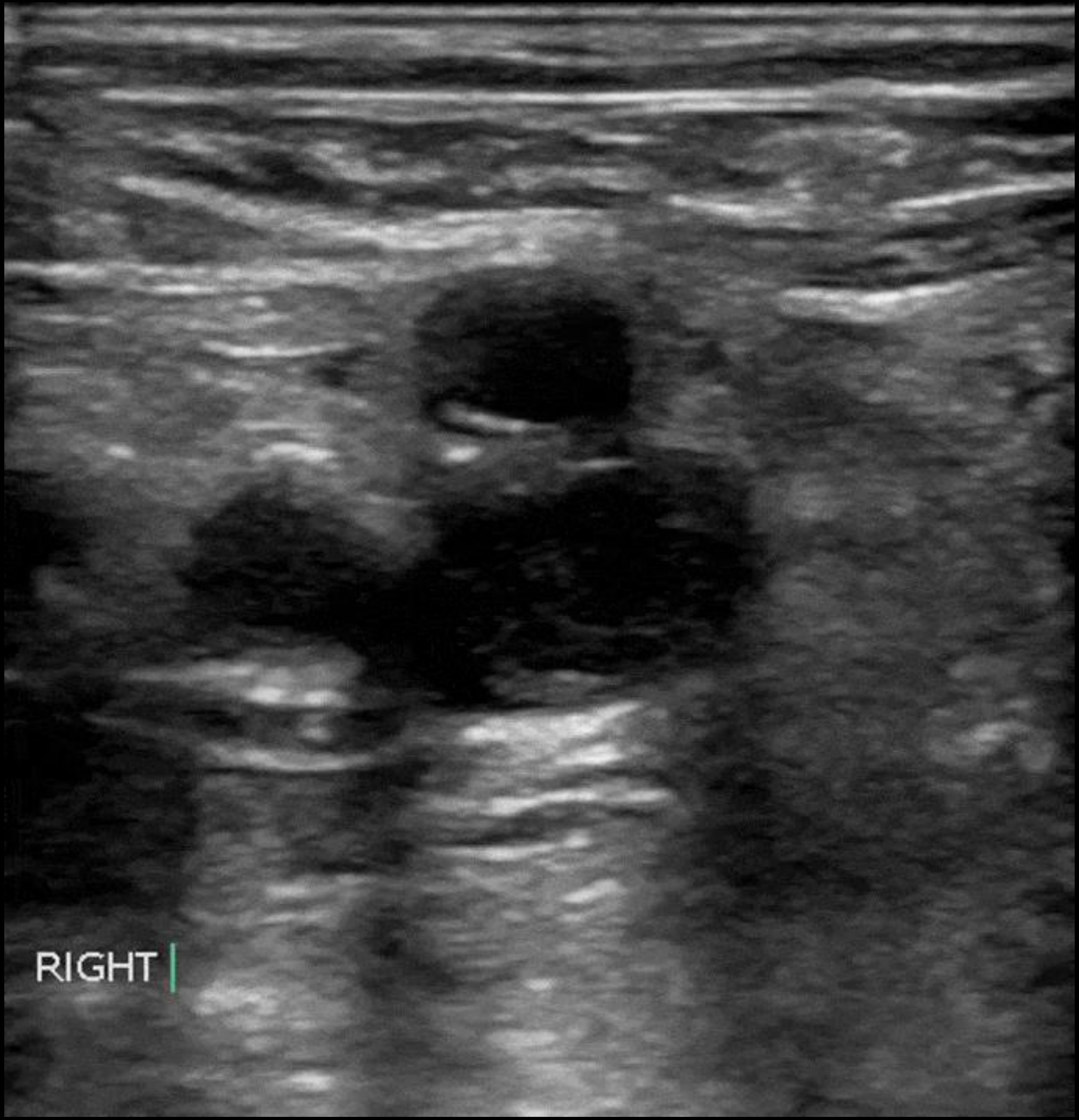

Venous Thrombosis

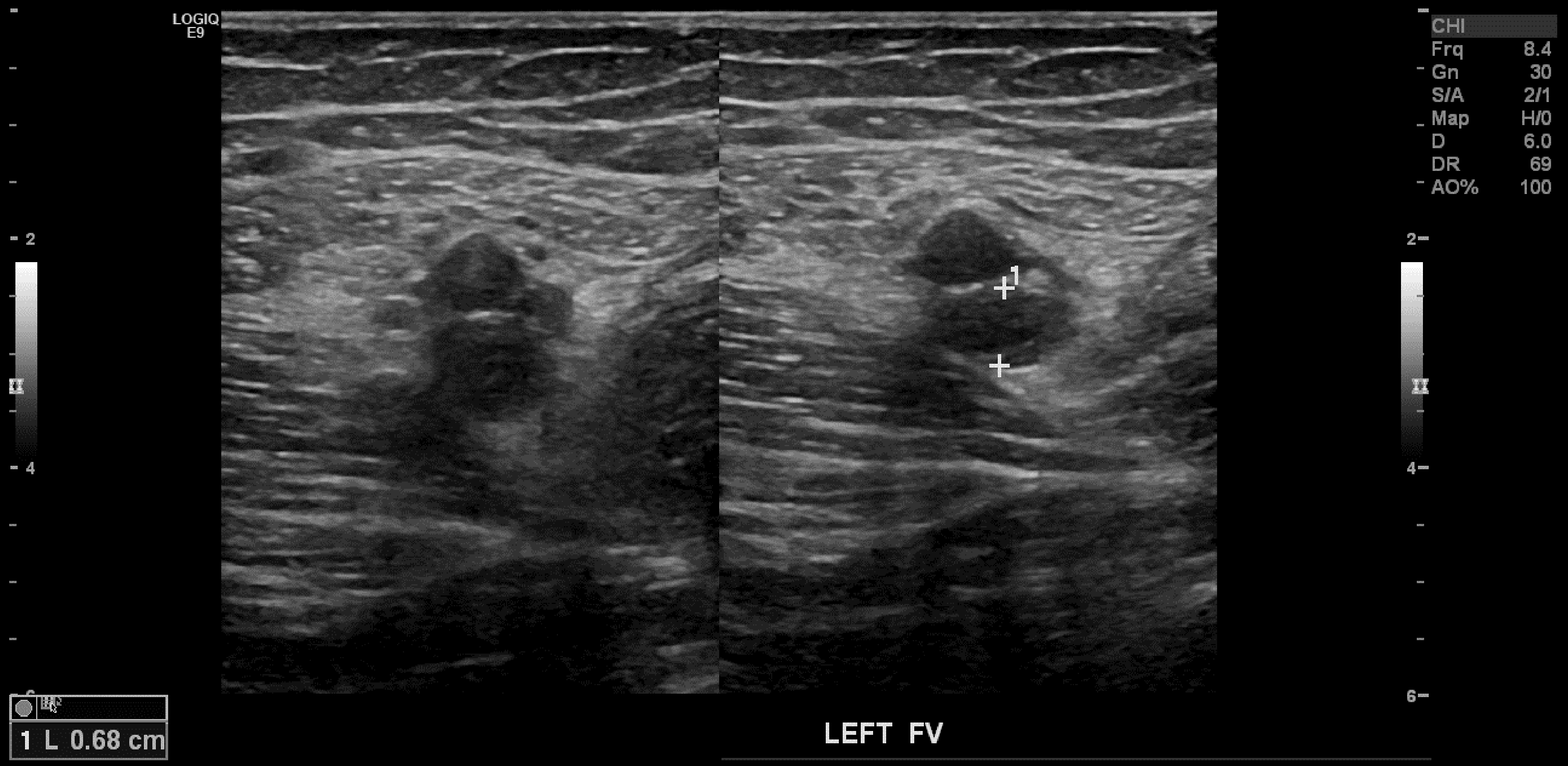
LOGIQ
E9
CHI
Frq 8.4
Gn 30
S/A 2/1
Map H/0
D 6.0
DR 69
AO% 100
1 L 0.68 cm
LEFT FV

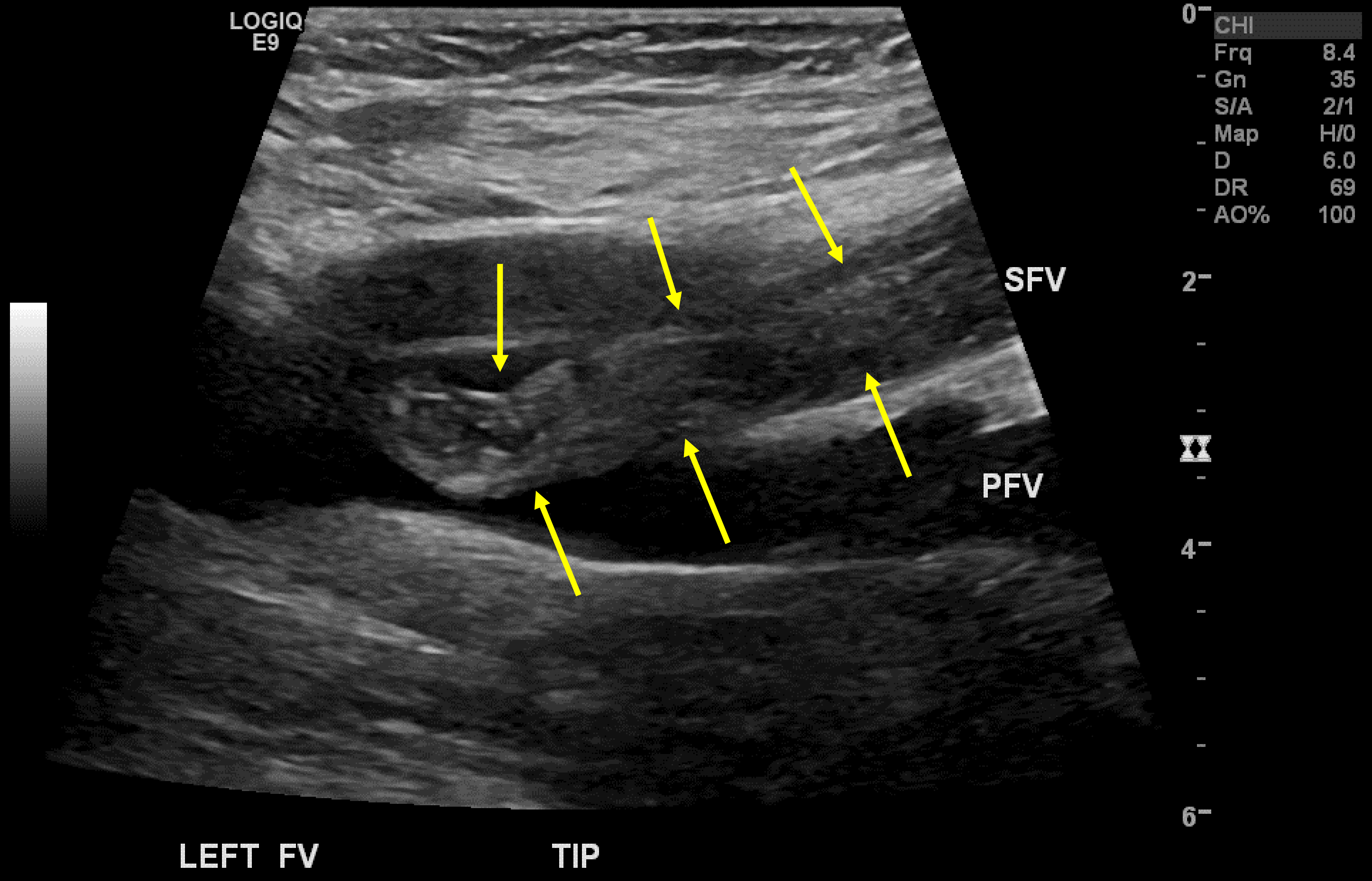
LOGIQ
E9
CHI
Frq 8.4
Gn 35
S/A 2/1
Map H/0
D 6.0
DR 69
AO% 100
0
2
4
6
SFV
PFV
LEFT FV
TIP

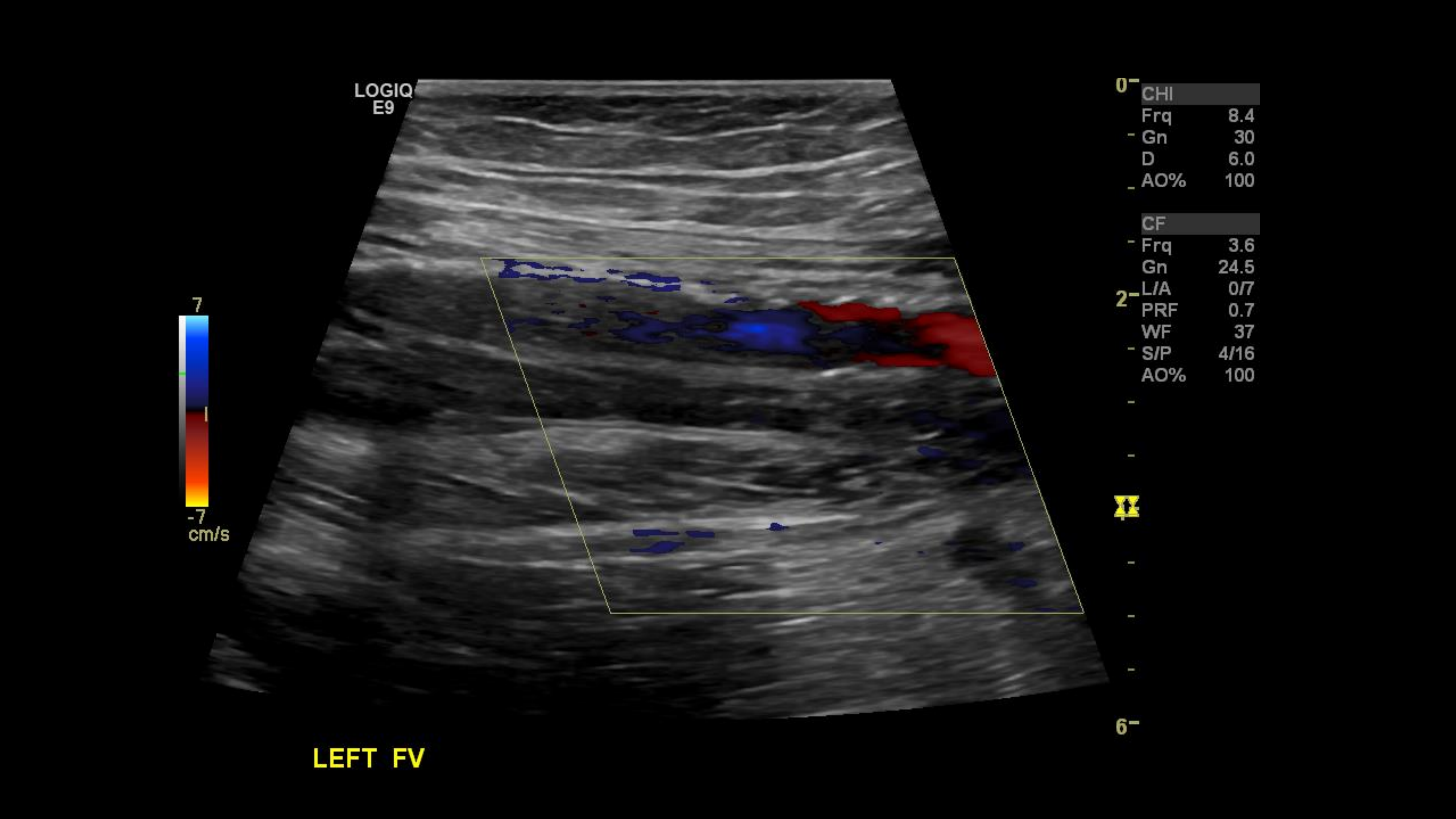
LOGIQ
E9
CHI
Frq 8.4
Gn 30
D 6.0
AO% 100
CF
Frq 3.6
Gn 24.5
L/A 0/7
PRF 0.7
WF 37
S/P 4/16
AO% 100
7
-7
cm/s
0
2
6
LEFT FV

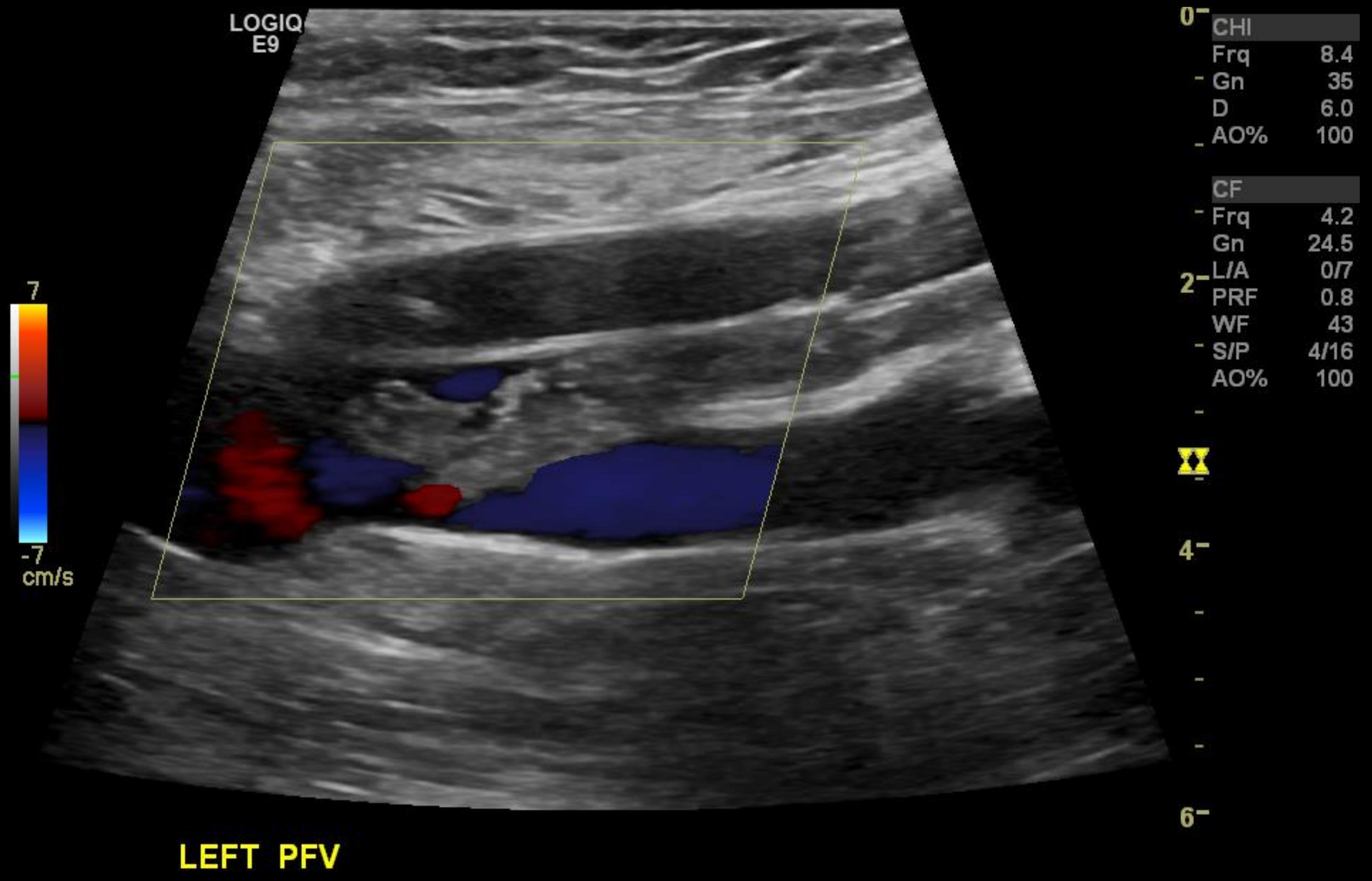
LOGIQ
E9
CHI
Frq 8.4
Gn 35
D 6.0
AO% 100
CF
Frq 4.2
Gn 24.5
L/A 0/7
PRF 0.8
WF 43
S/P 4/16
AO% 100
7
-7
cm/s
0
2
4
6
LEFT PFV

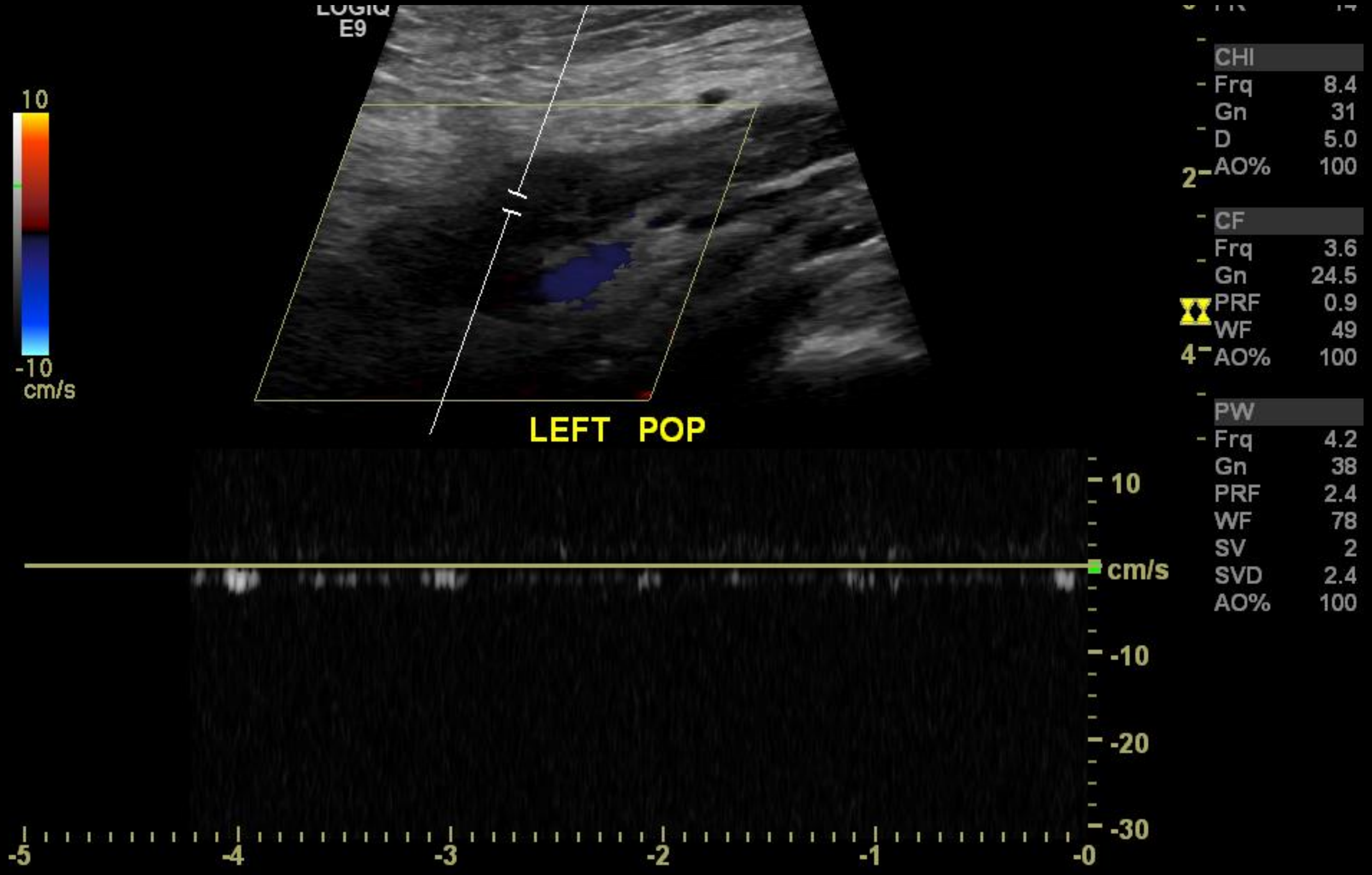
LOGIQ
E9
10
-10
cm/s
LEFT POP
CHI
Frq 8.4
Gn 31
D 5.0
AO% 100
CF
Frq 3.6
Gn 24.5
PRF 0.9
WF 49
AO% 100
PW
Frq 4.2
Gn 38
PRF 2.4
WF 78
SV 2
SVD 2.4
AO% 100
10
cm/s
-10
-20
-30
-5
-4
-3
-2
-1
-0

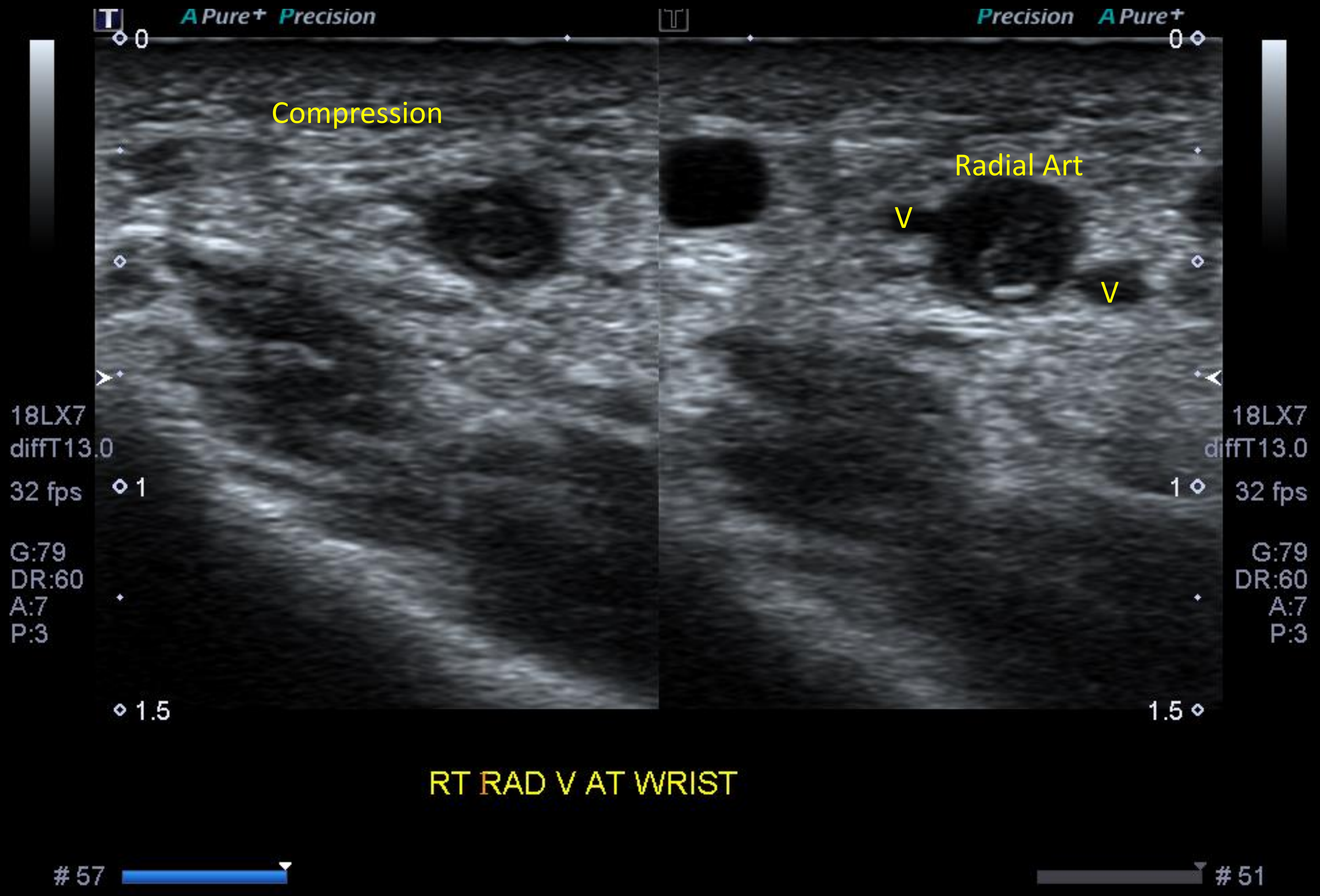
A Pure+ Precision
Precision A Pure+
Compression
Radial Art
V
V
18LX7
diffT13.0
32 fps
G:79
DR:60
A:7
P:3
18LX7
diffT13.0
32 fps
G:79
DR:60
A:7
P:3
0
1
1.5
0
1
1.5
RT RAD V AT WRIST
57
51

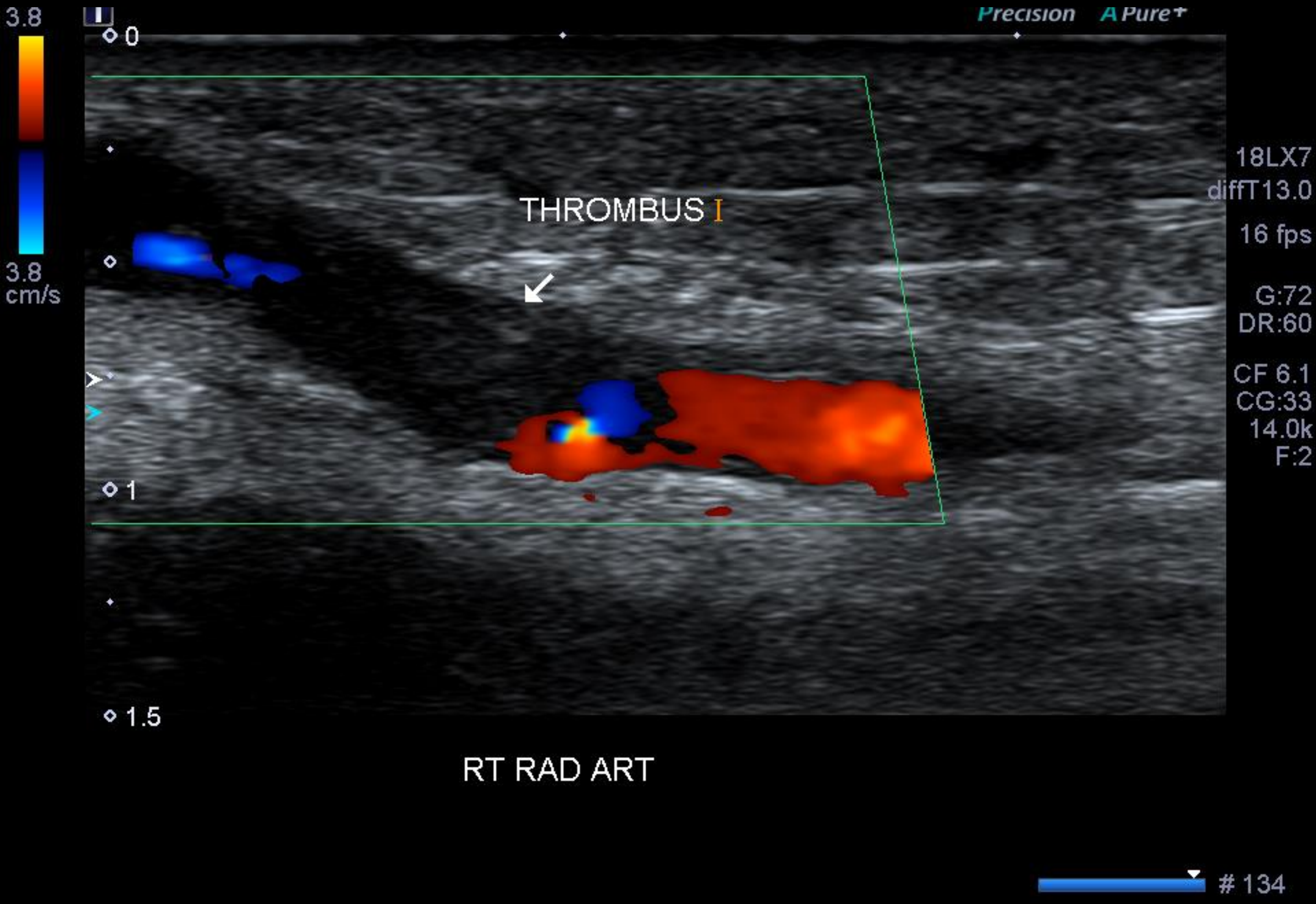

3.8
3.8
cm/s
Precision
Pure+
0
1
1.5
THROMBUS
18LX7
diffT13.0
16 fps
G:72
DR:60
CF 6.1
CG:33
14.0k
F:2
RT RAD ART
134

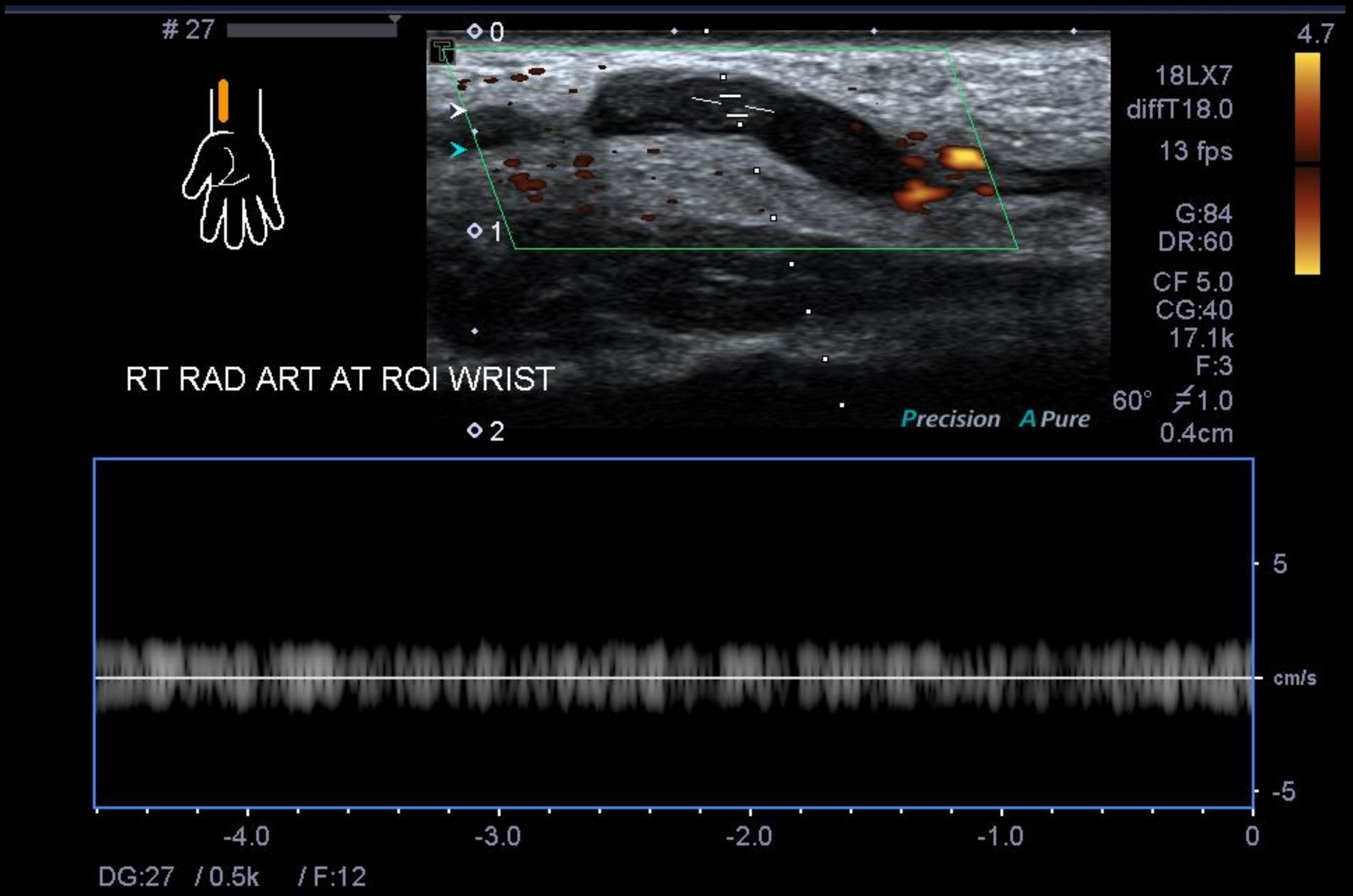

27
0
1
2
RT RAD ART AT ROI WRIST
4.7
18LX7
diffT18.0
13 fps
G:84
DR:60
CF 5.0
CG:40
17.1k
F:3
60°
1.0
0.4cm
Precision
A Pure
5
cm/s
-5
-4.0
-3.0
-2.0
-1.0
0
DG:27 / 0.5k / F:12

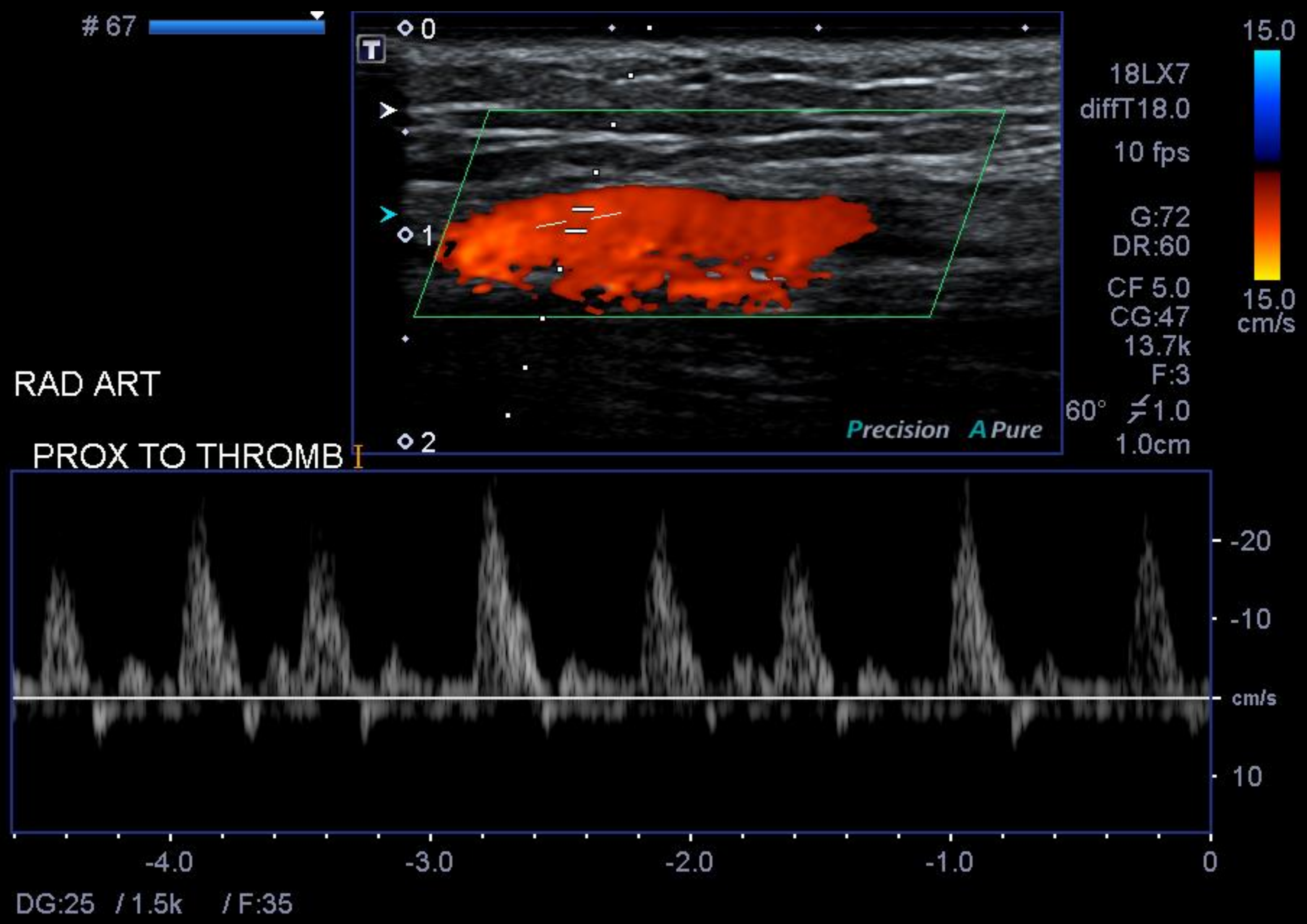

67
RAD ART
PROX TO THROMB
0
1
2
Precision
A Pure
18LX7
diffT18.0
10 fps
G:72
DR:60
CF 5.0
CG:47
13.7k
F:3
60°
1.0
1.0cm
15.0
15.0
cm/s
-20
-10
cm/s
10
-4.0
-3.0
-2.0
-1.0
0
DG:25 / 1.5k / F:35

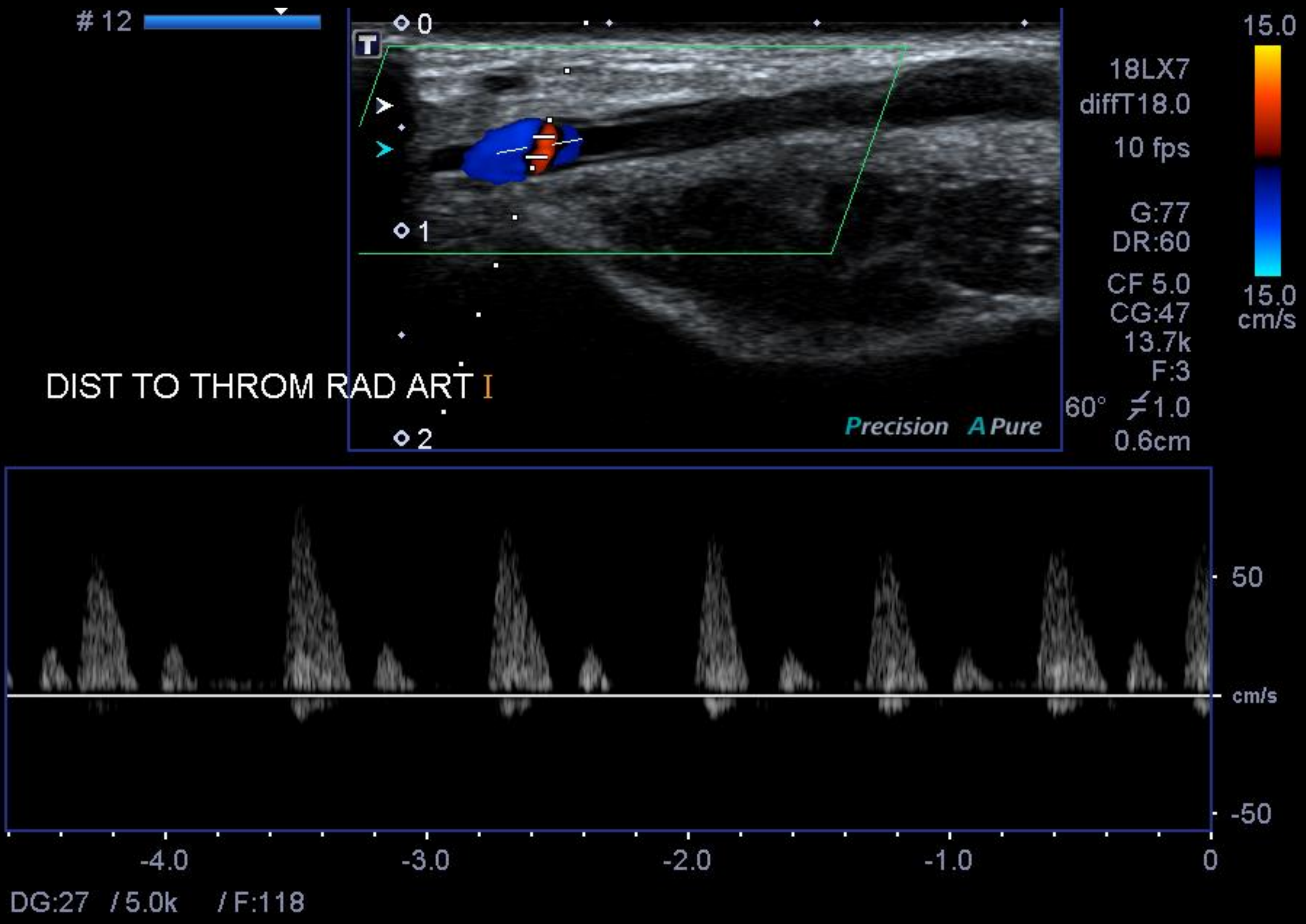

12
18LX7
diffT18.0
10 fps
G:77
DR:60
CF 5.0
CG:47
13.7k
F:3
60° 1.0
0.6cm
15.0
15.0
cm/s
DIST TO THROM RAD ART
Precision A Pure
50
cm/s
-50
-4.0
-3.0
-2.0
-1.0
0
DG:27 / 5.0k / F:118

Arterial Thrombus

Practice Cases

Case 1

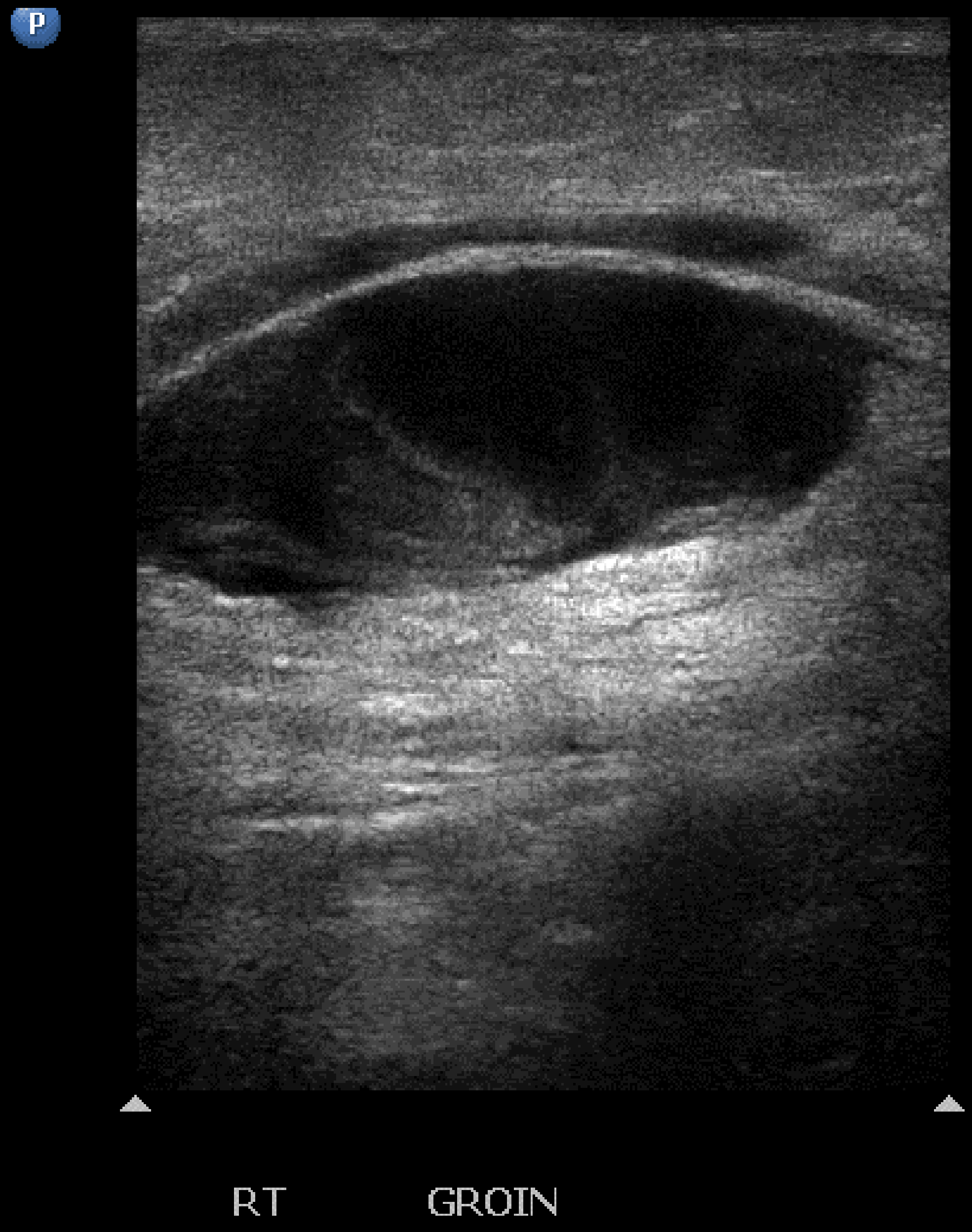
RT GROIN

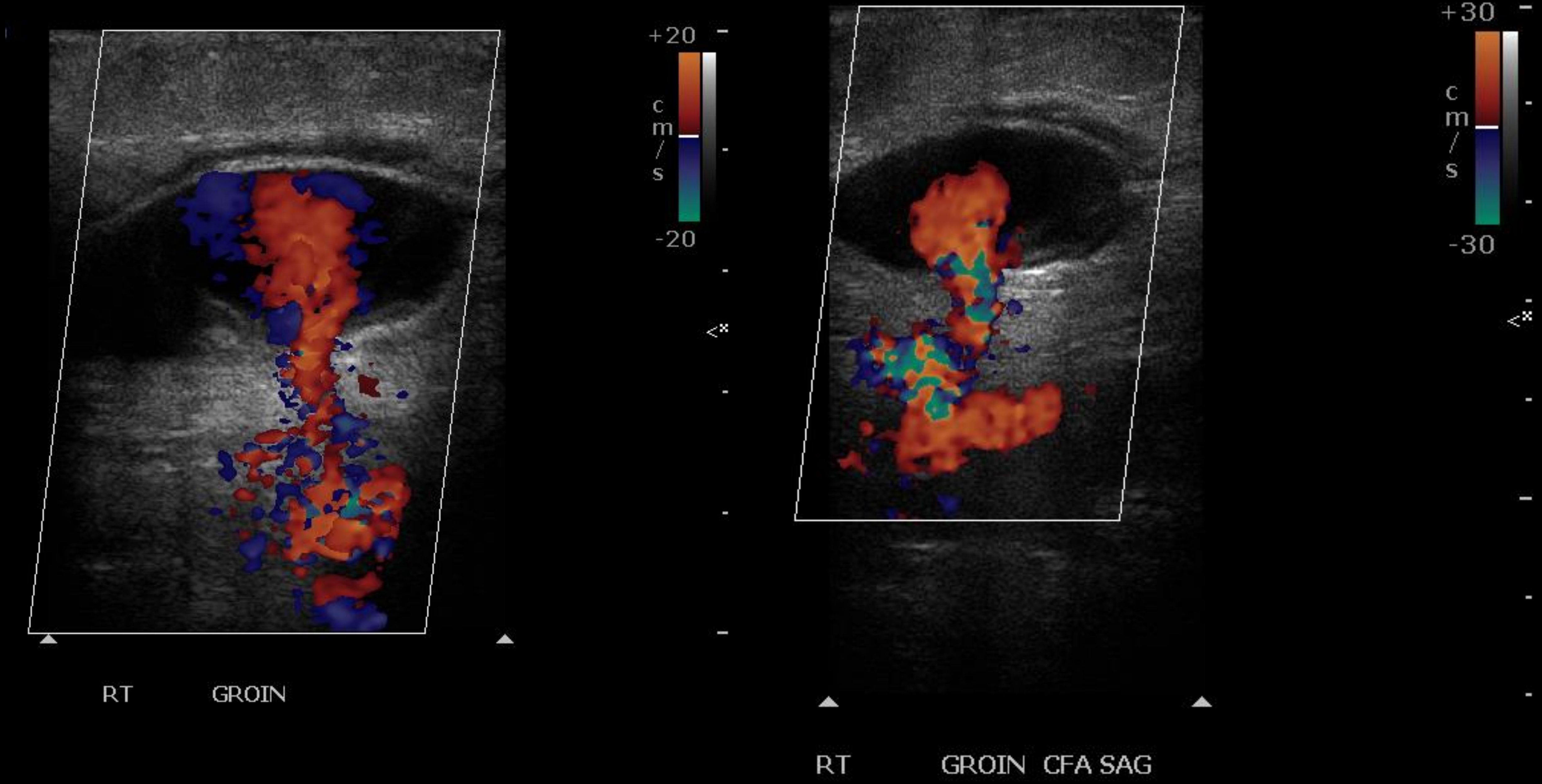
+20
cm/s
-20
RT GROIN
+30
cm/s
-30
RT GROIN CFA SAG

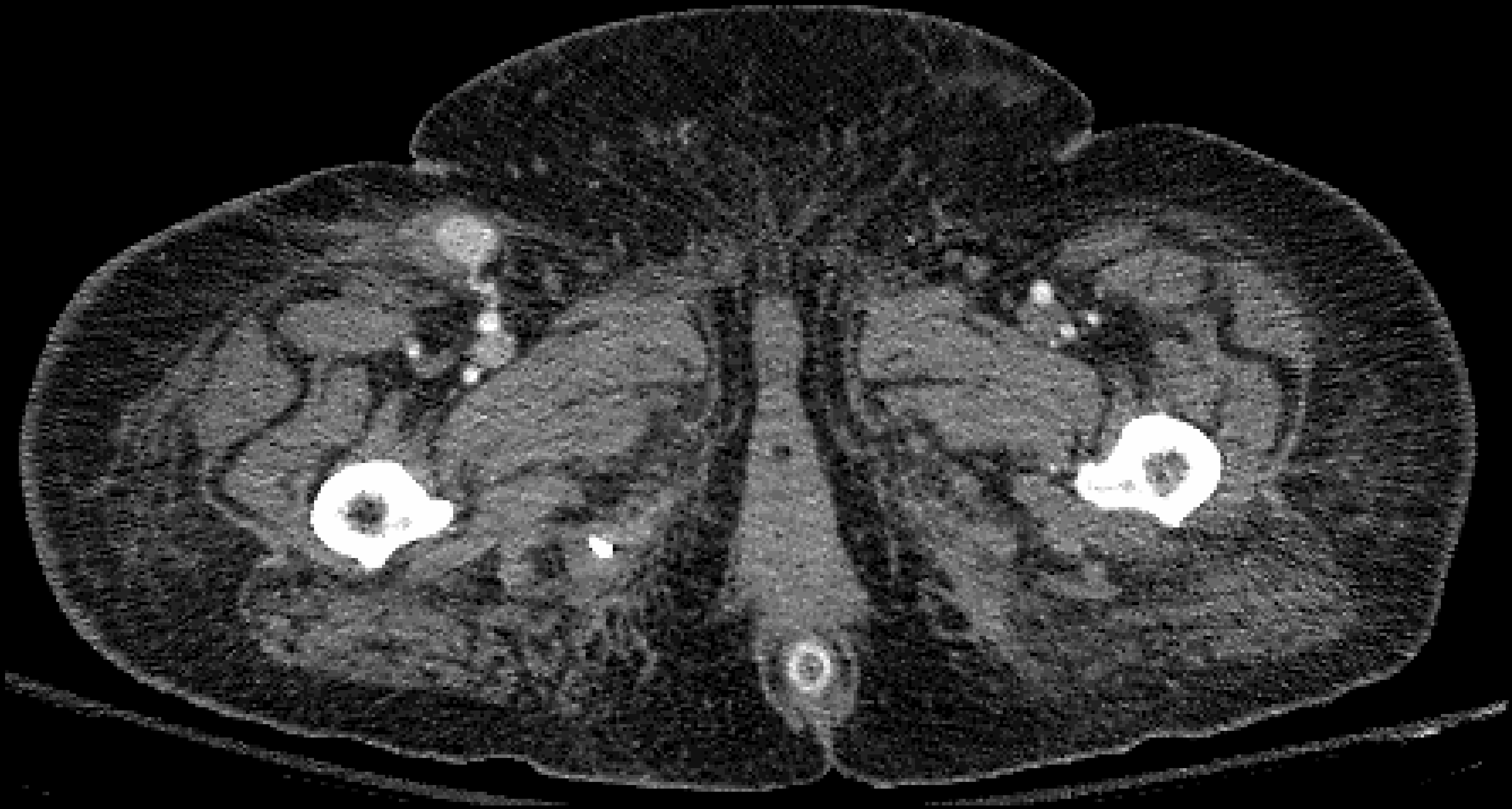

Partially thrombosed pseudoaneurysm

Case 2

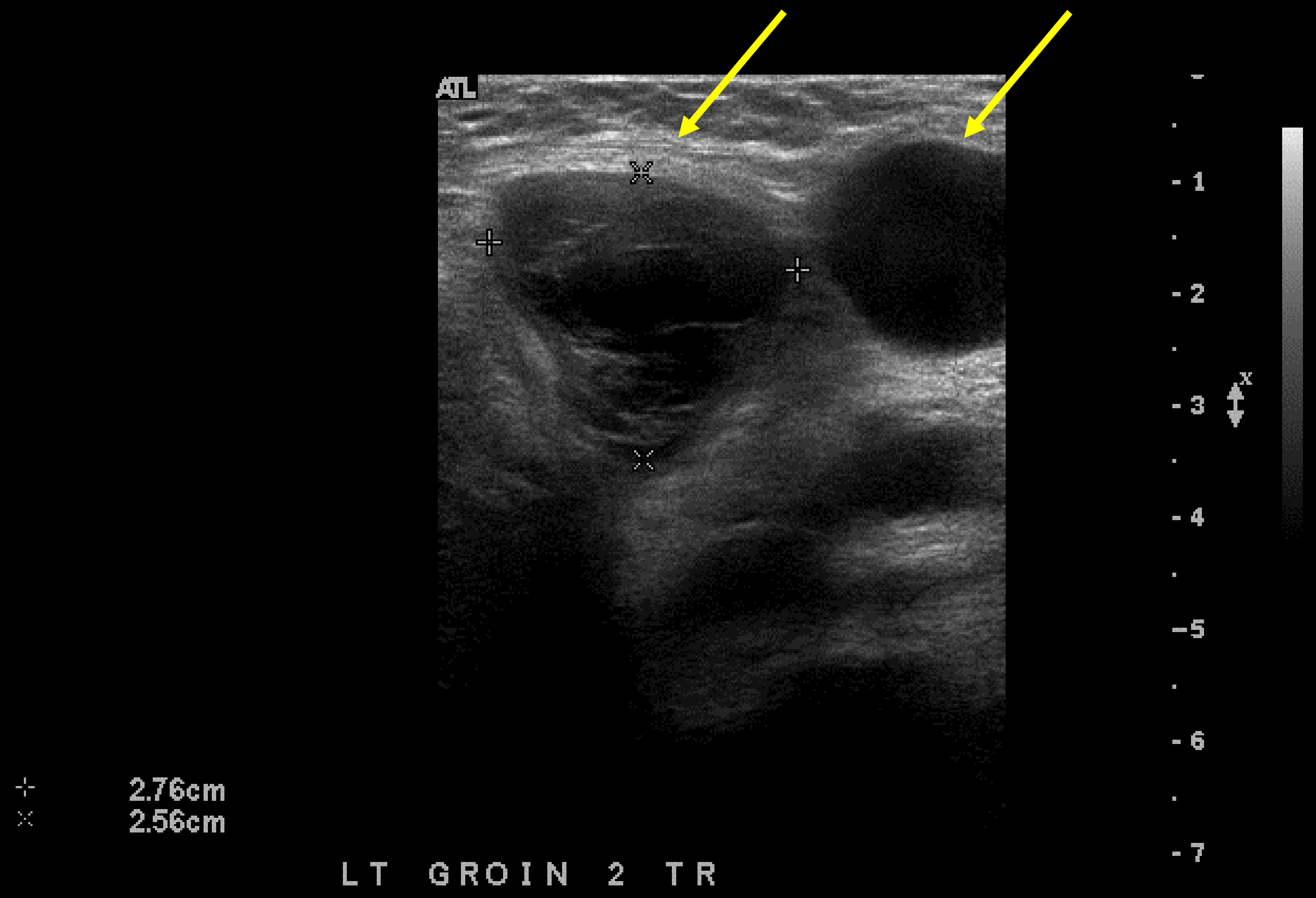
ATL
1
2
3
4
5
6
7
2.76cm
2.56cm
LT GROIN 2 TR

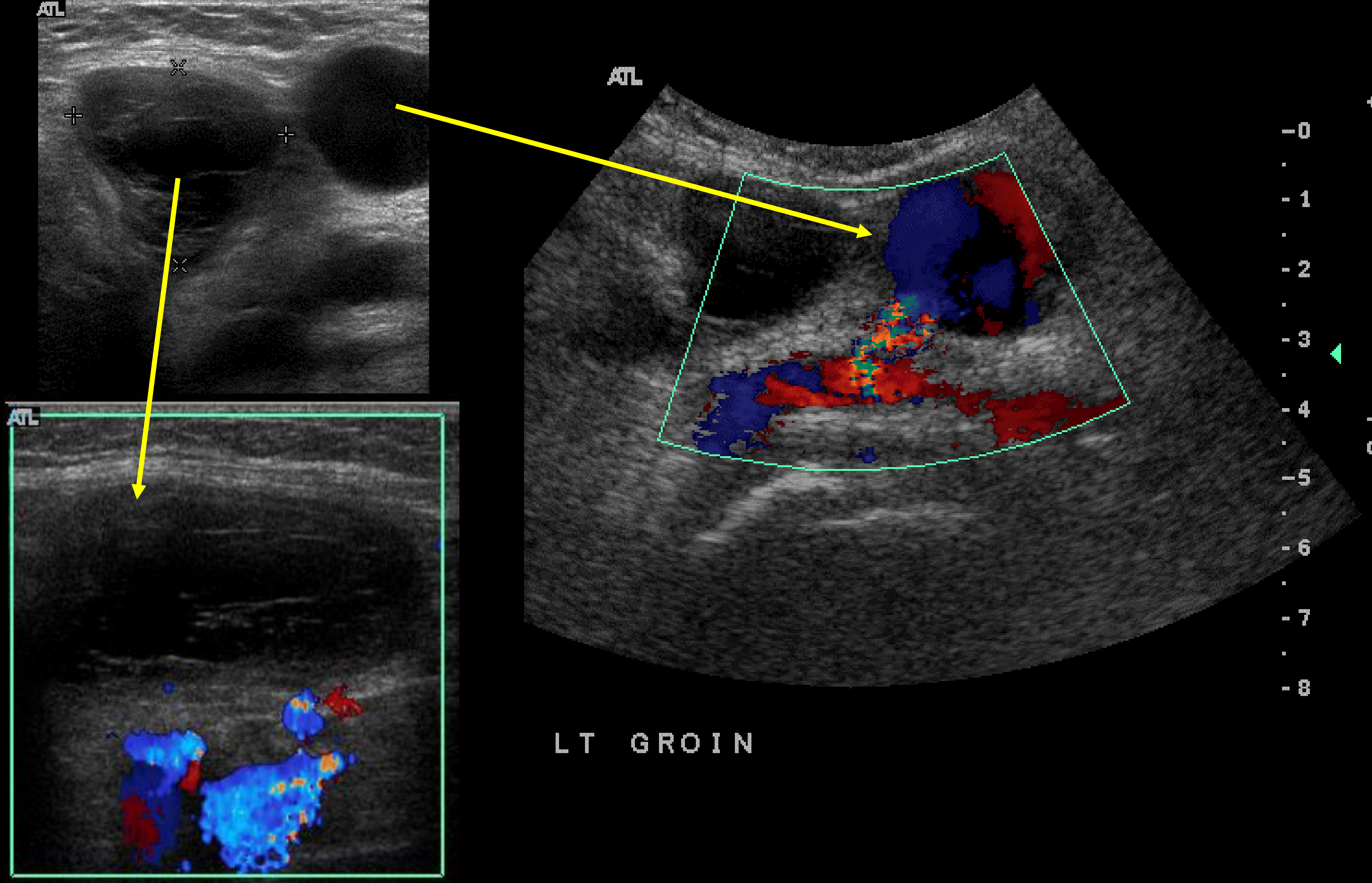
ATL
ATL
ATL
+ 23.0
− 23.0
cm/s
0
1
2
3
4
5
6
7
8
LT GROIN

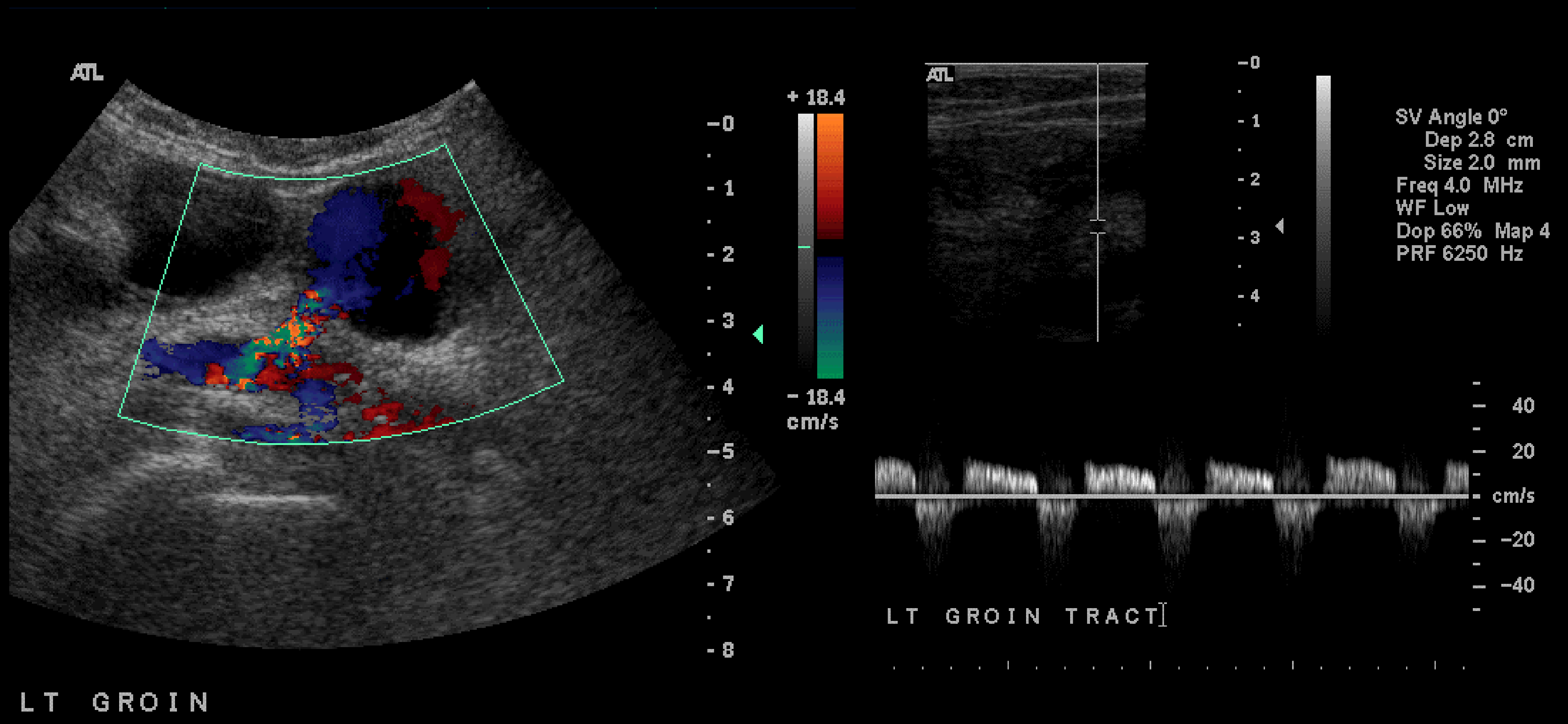
ATL
+ 18.4
- 18.4
cm/s
ATL
SV Angle 0°
Dep 2.8 cm
Size 2.0 mm
Freq 4.0 MHz
WF Low
Dop 66% Map 4
PRF 6250 Hz
40
20
cm/s
-20
-40
LT GROIN TRACT
LT GROIN

Hematoma and pseudoaneurysm

Case 3

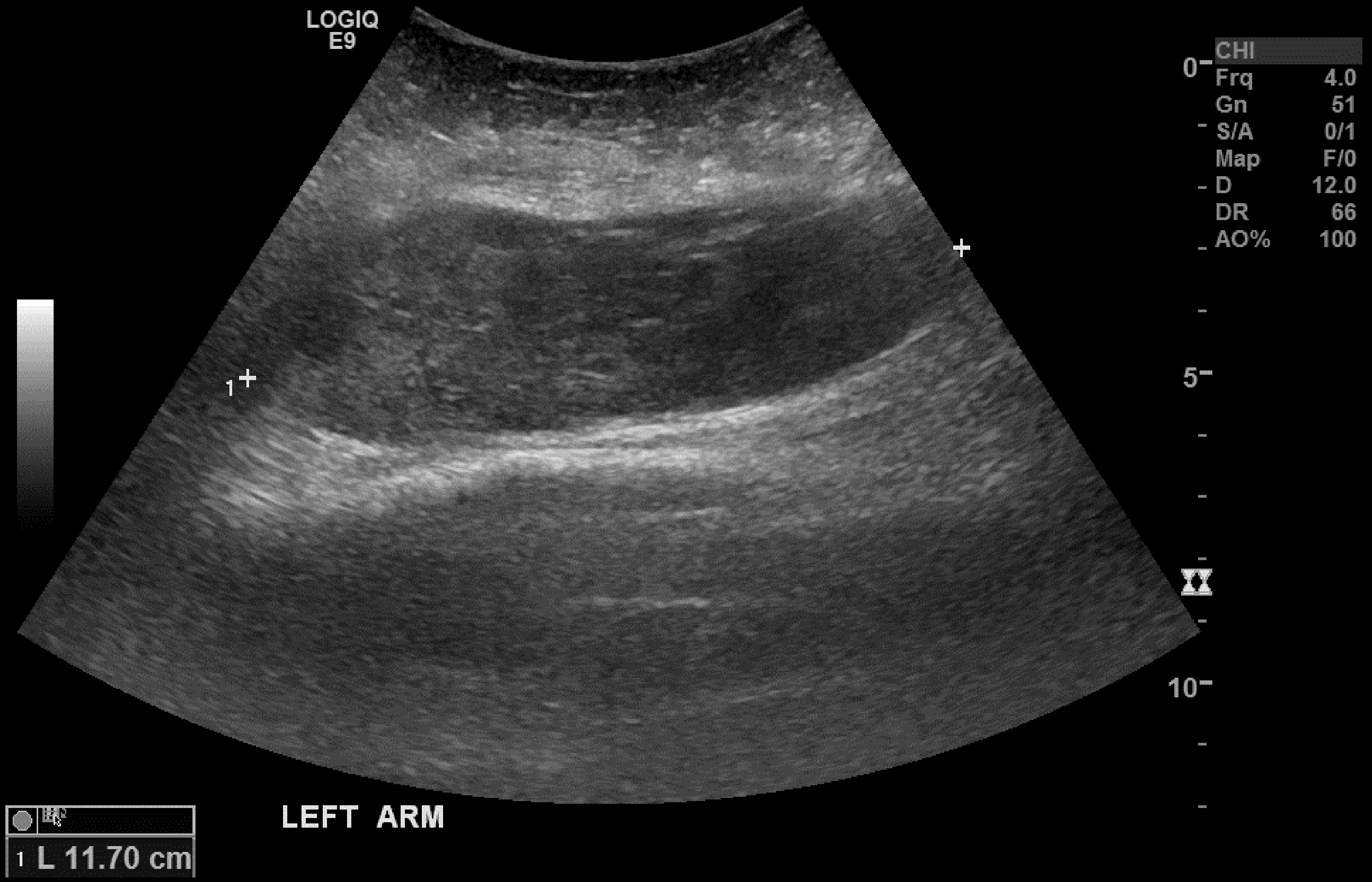
LOGIQ
E9
CHI
Frq 4.0
Gn 51
S/A 0/1
Map F/0
D 12.0
DR 66
AO% 100
0
5
10
1
LEFT ARM
1 L 11.70 cm

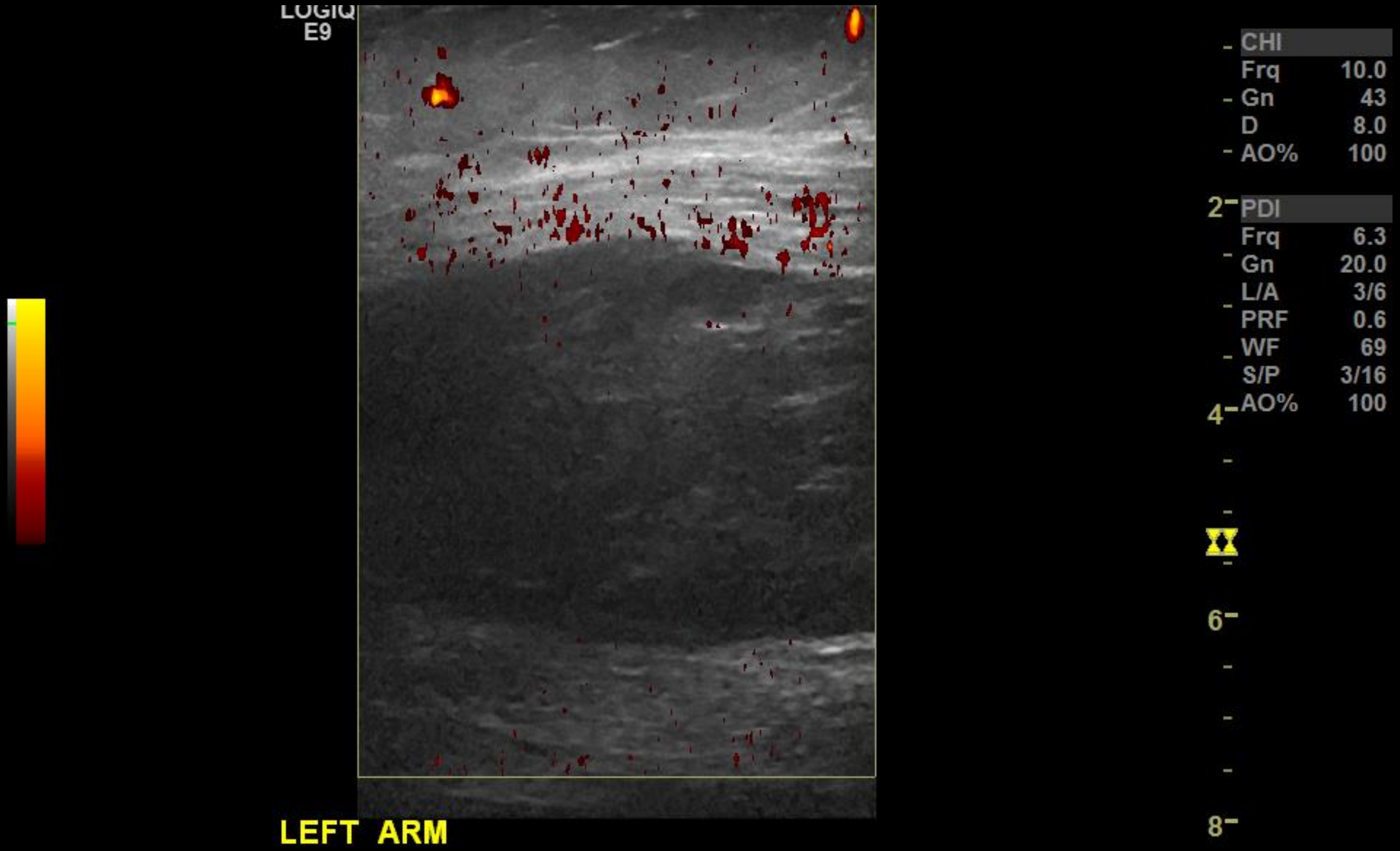
LOGIQ
E9
CHI
Frq 10.0
Gn 43
D 8.0
AO% 100
PDI
Frq 6.3
Gn 20.0
L/A 3/6
PRF 0.6
WF 69
S/P 3/16
AO% 100
2
4
6
8
LEFT ARM

Hematoma

Case 4

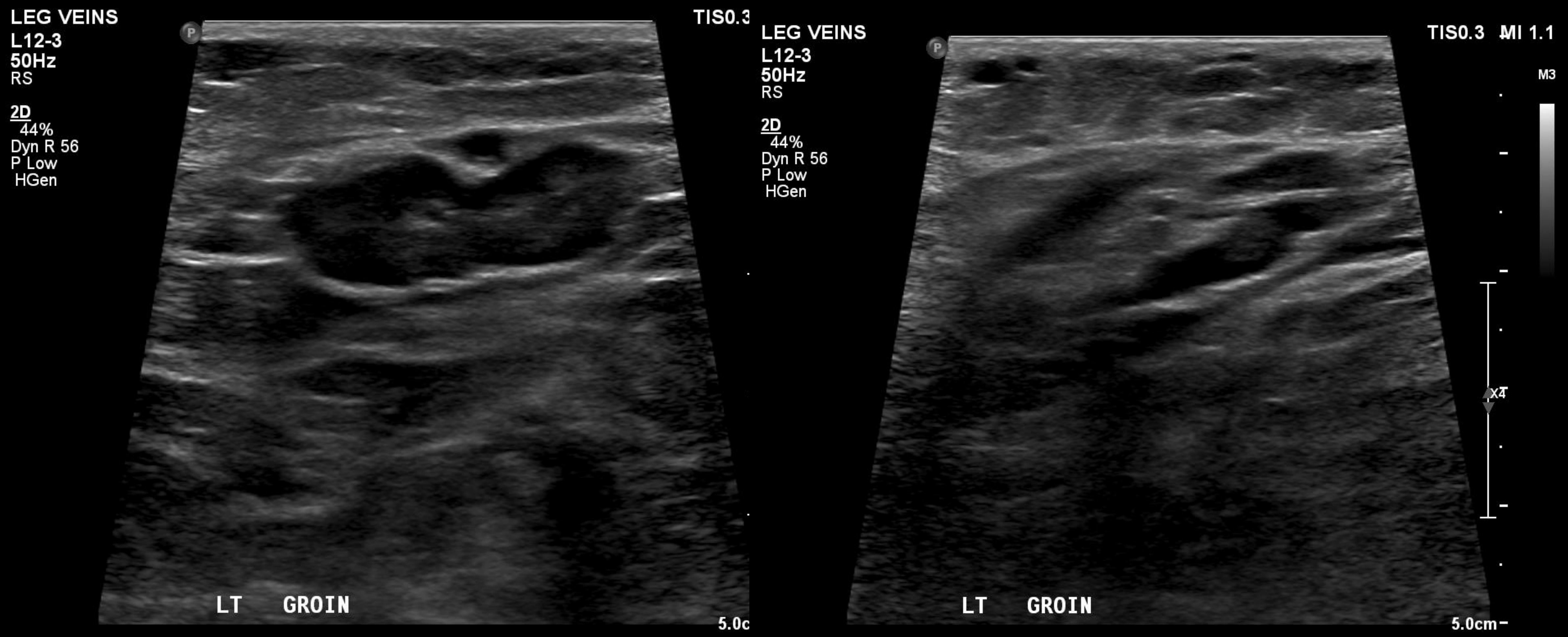
LEG VEINS
L12-3
50Hz
RS
2D
44%
Dyn R 56
P Low
HGen
TIS0.3
LT GROIN
5.0c
LEG VEINS
L12-3
50Hz
RS
2D
44%
Dyn R 56
P Low
HGen
TIS0.3 MI 1.1
M3
x4
LT GROIN
5.0cm

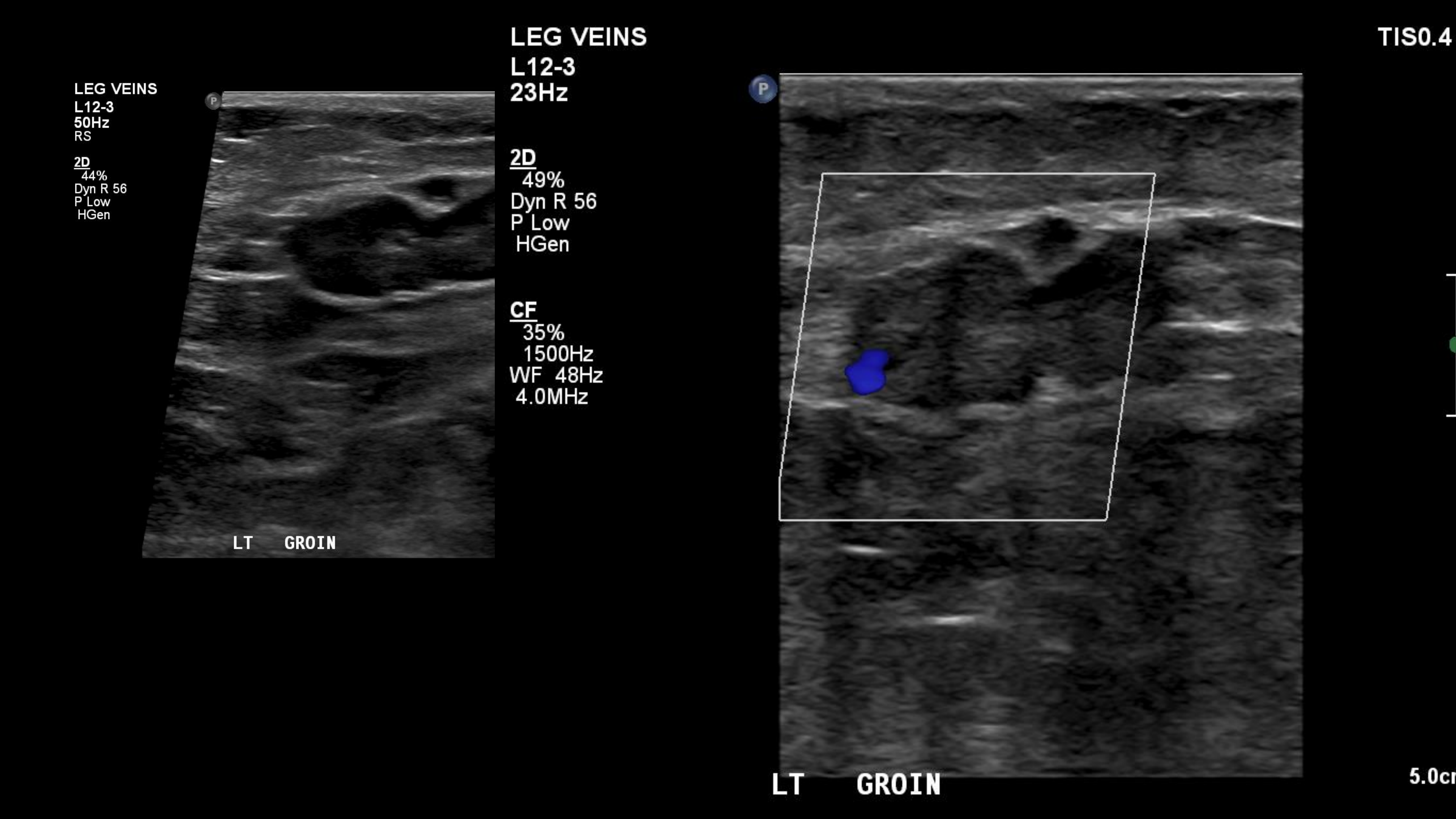
LEG VEINS
L12-3
50Hz
RS
2D
44%
Dyn R 56
P Low
HGen
LT GROIN
LEG VEINS
L12-3
23Hz
2D
49%
Dyn R 56
P Low
HGen
CF
35%
1500Hz
WF 48Hz
4.0MHz
TIS0.4
LT GROIN
5.0cm

Inguinal Lymph Node

Case 5

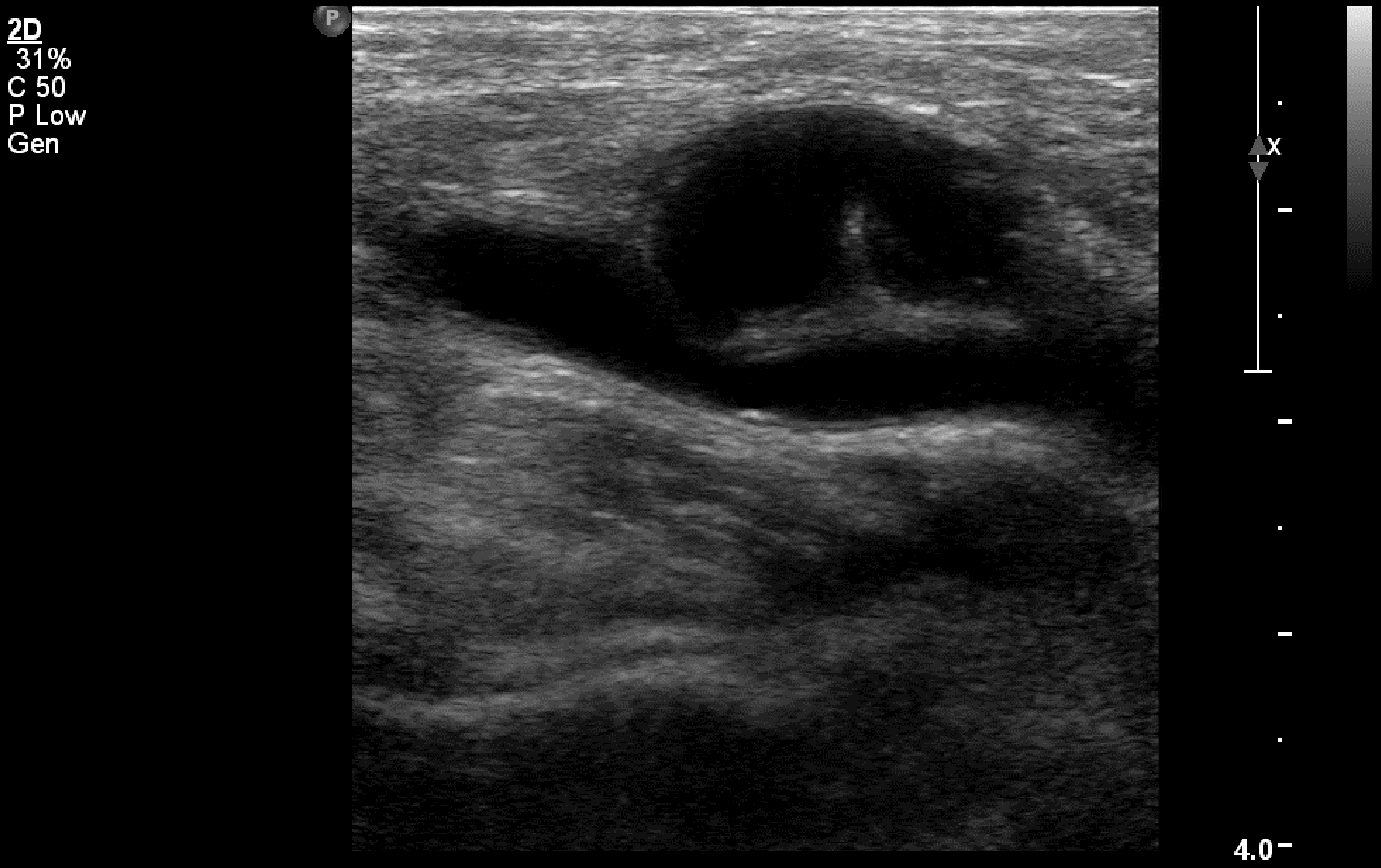

R inguinal region

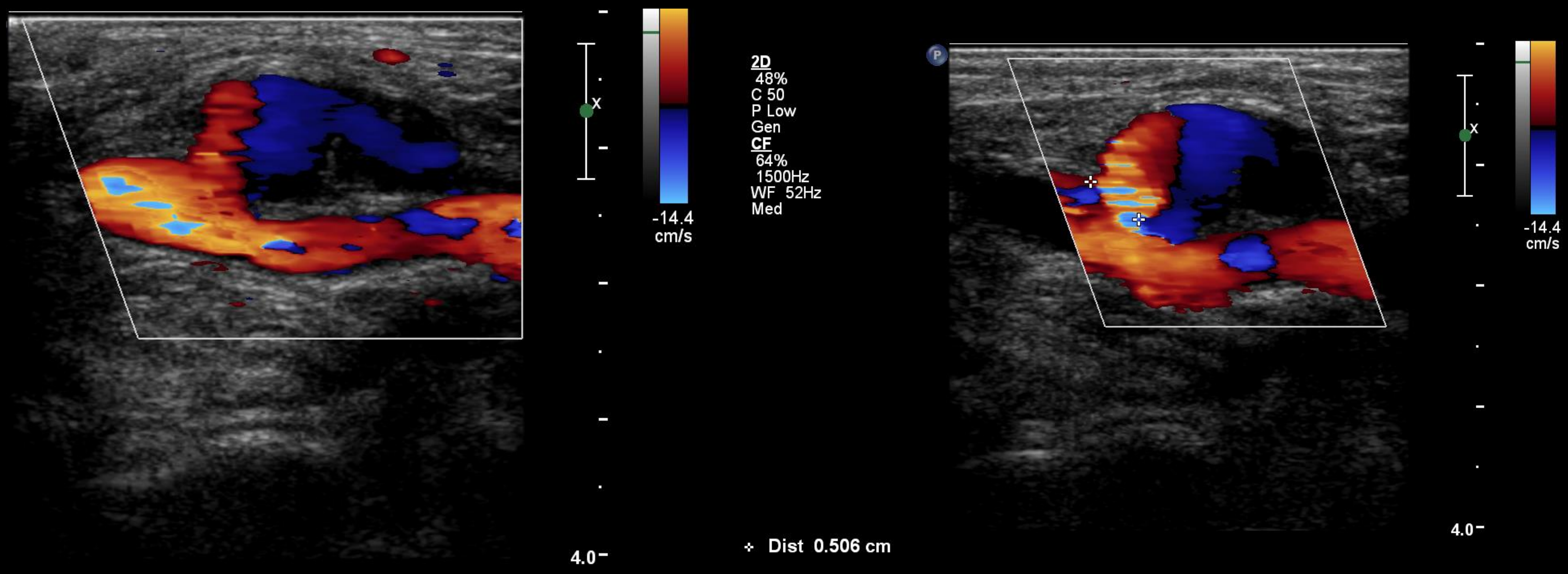

-14.4
cm/s
2D
48%
C 50
P Low
Gen
CF
64%
1500Hz
WF 52Hz
Med
4.0
Dist 0.506 cm
-14.4
cm/s
4.0

Pseudoaneurysm
(short, thick neck)

Case 6

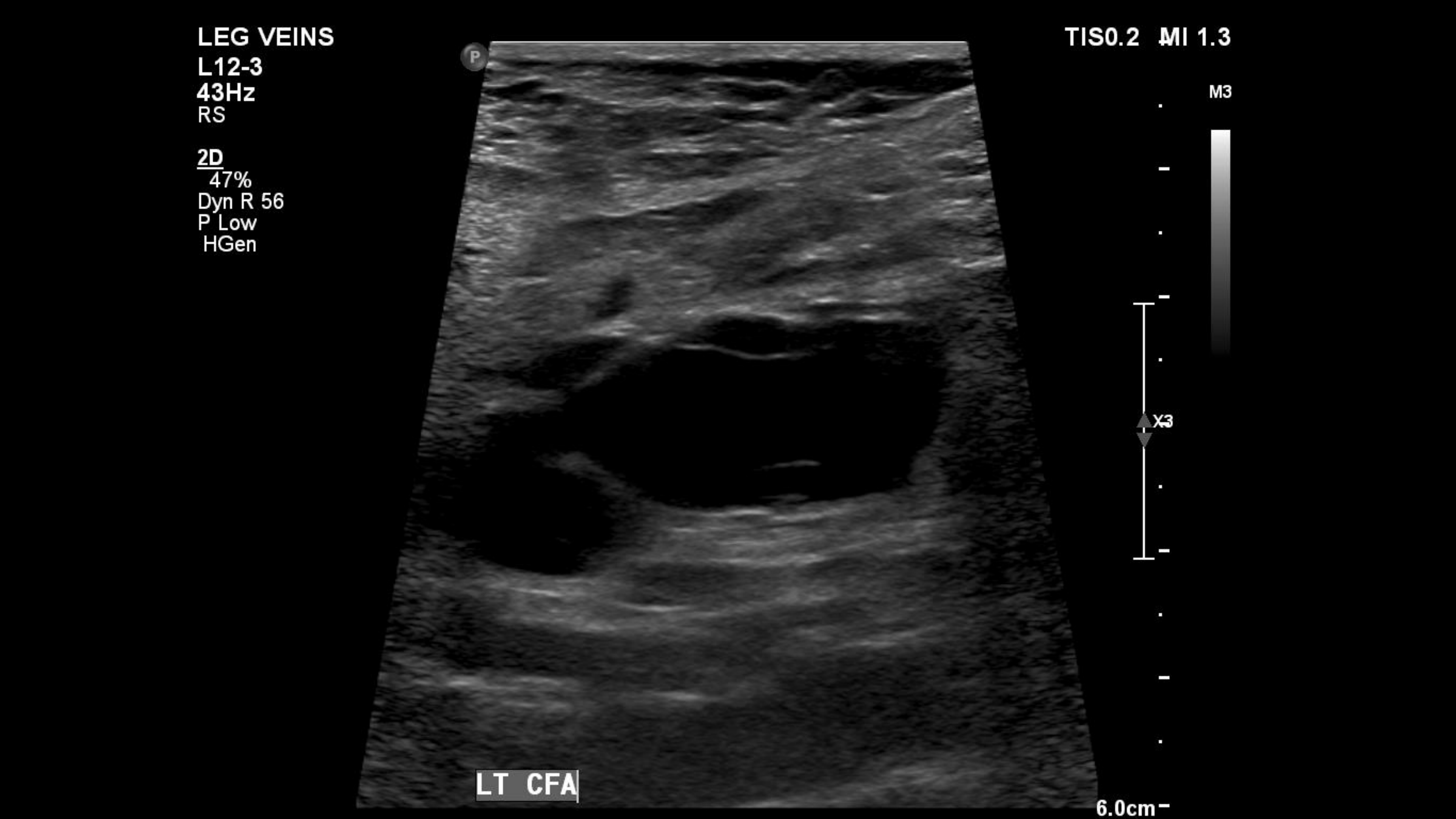

LEG VEINS
L12-3
43Hz
RS
2D
47%
Dyn R 56
P Low
HGen
TIS0.2 MI 1.3
M3
X3
LT CFA
6.0cm

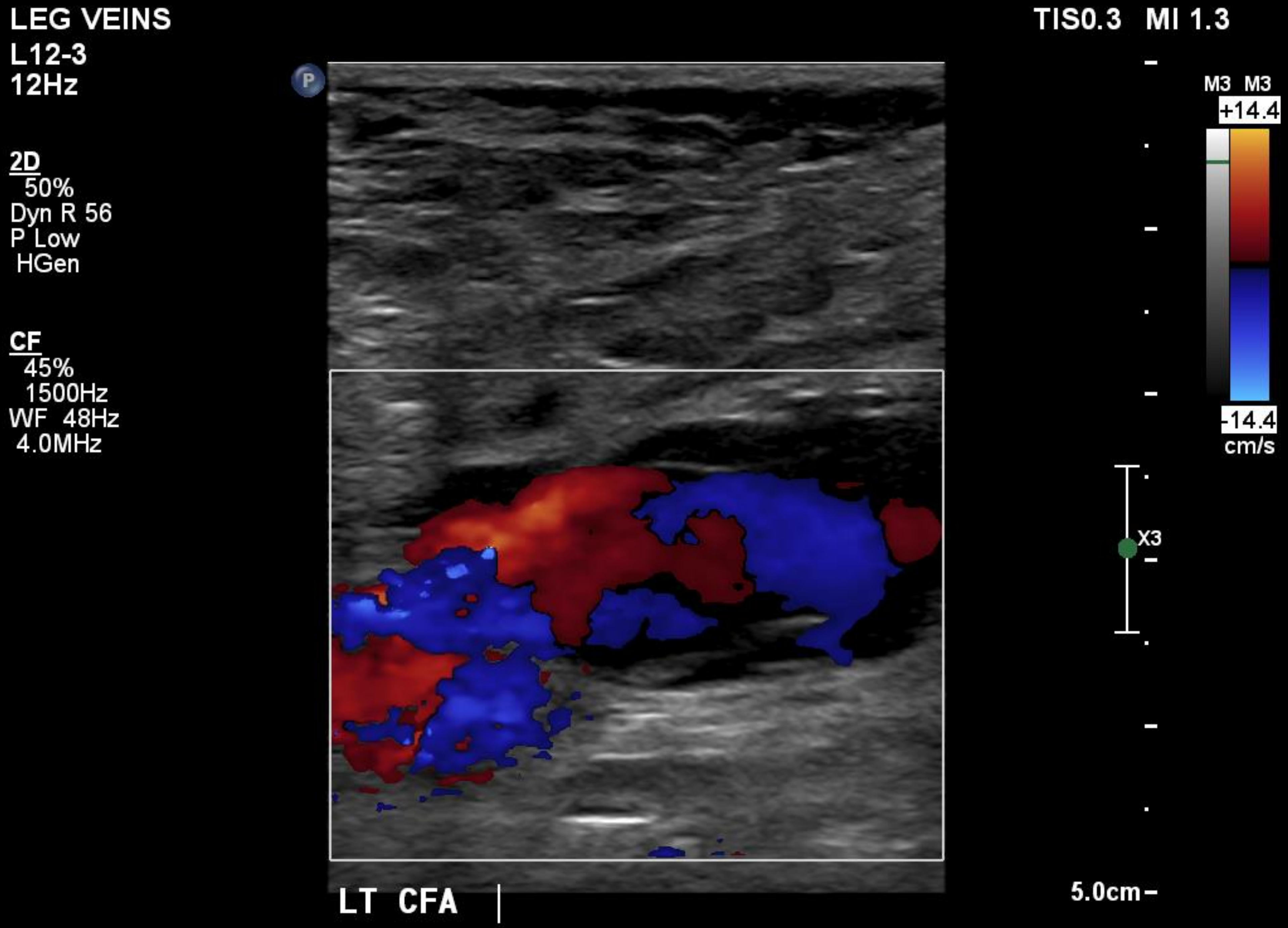
LEG VEINS
L12-3
12Hz
2D
50%
Dyn R 56
P Low
HGen
CF
45%
1500Hz
WF 48Hz
4.0MHz
TIS0.3 MI 1.3
M3 M3
+14.4
-14.4
cm/s
X3
5.0cm
LT CFA

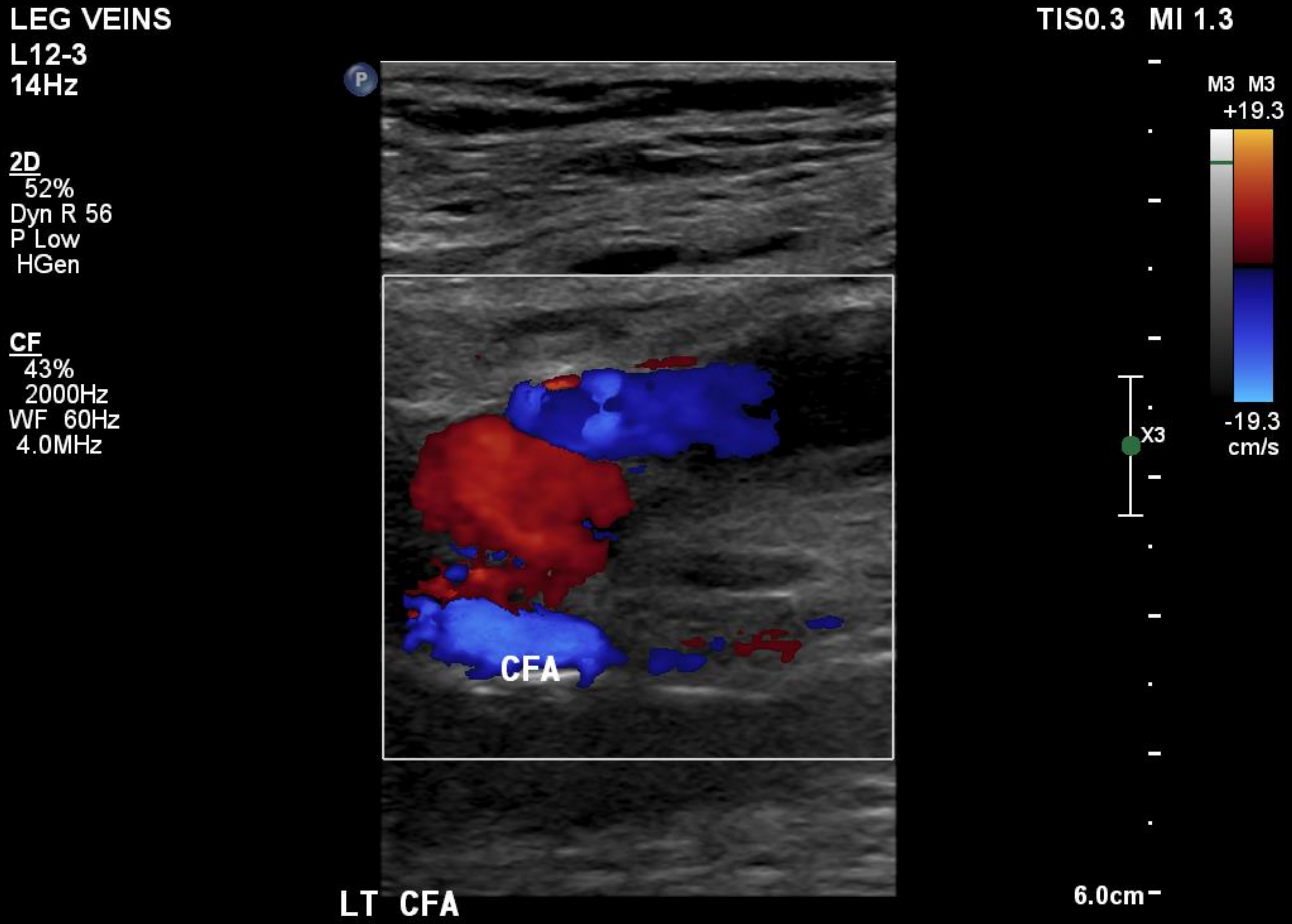
LEG VEINS
L12-3
14Hz
2D
52%
Dyn R 56
P Low
HGen
CF
43%
2000Hz
WF 60Hz
4.0MHz
TIS0.3 MI 1.3
M3 M3
+19.3
-19.3
cm/s
X3
CFA
6.0cm
LT CFA

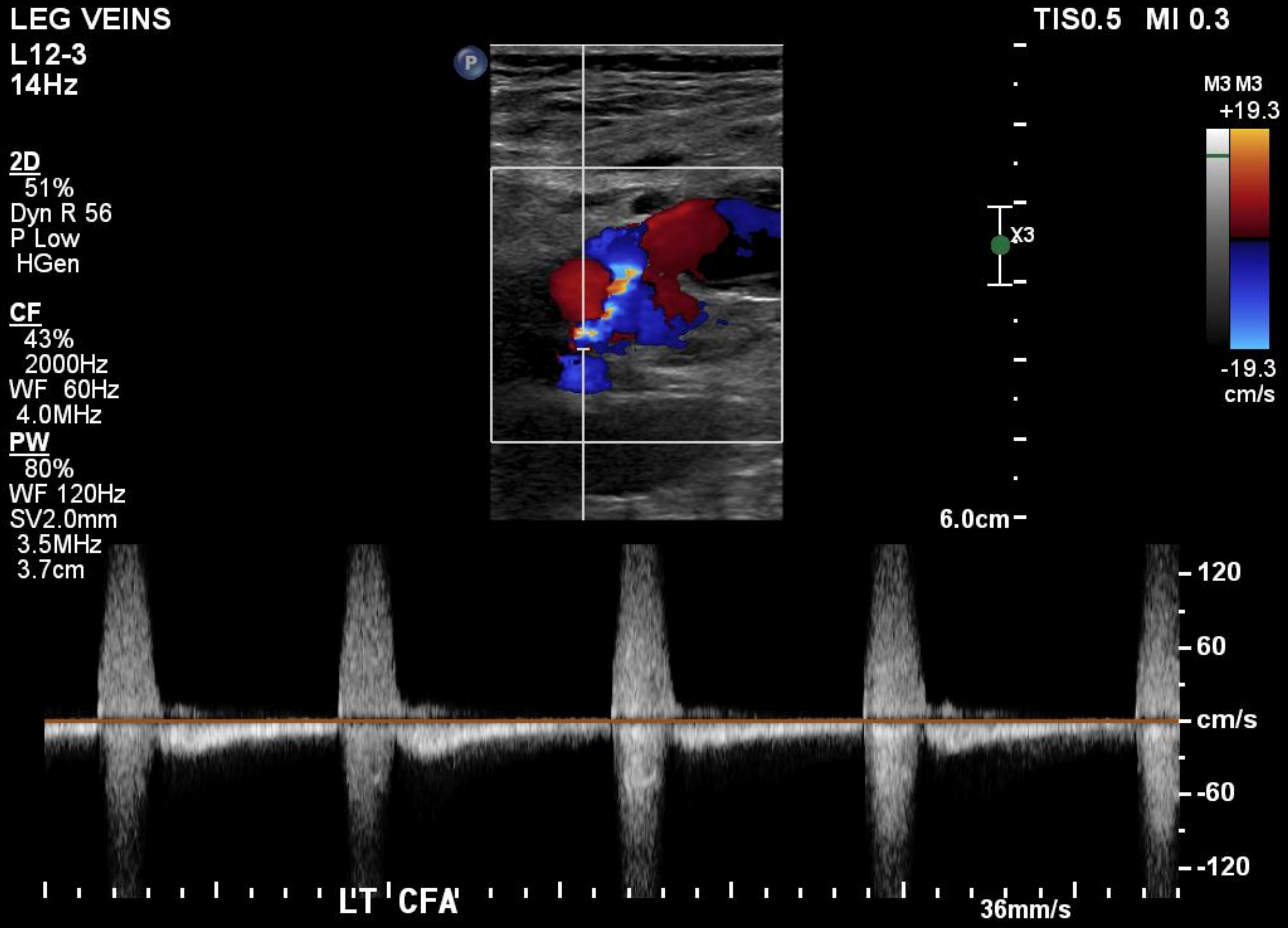
LEG VEINS
L12-3
14Hz
2D
51%
Dyn R 56
P Low
HGen
CF
43%
2000Hz
WF 60Hz
4.0MHz
PW
80%
WF 120Hz
SV2.0mm
3.5MHz
3.7cm
TIS0.5 MI 0.3
M3 M3
+19.3
-19.3
cm/s
X3
6.0cm
120
60
cm/s
-60
-120
LT CFA
36mm/s

Pseudoaneurysm

Case 7

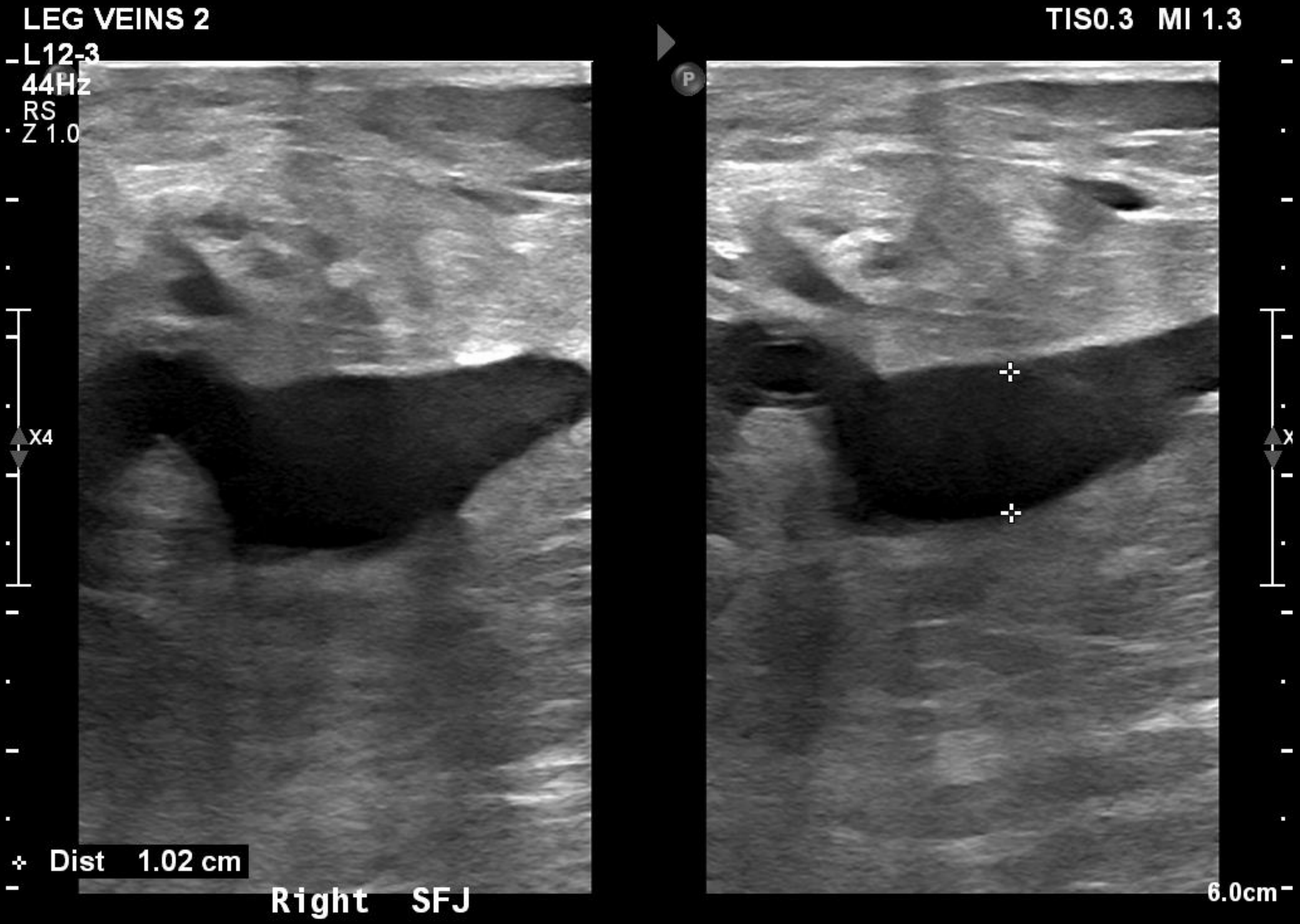
LEG VEINS 2
L12-3
44Hz
RS
Z 1.0
TIS0.3 MI 1.3
X4
Dist 1.02 cm
Right SFJ
6.0cm

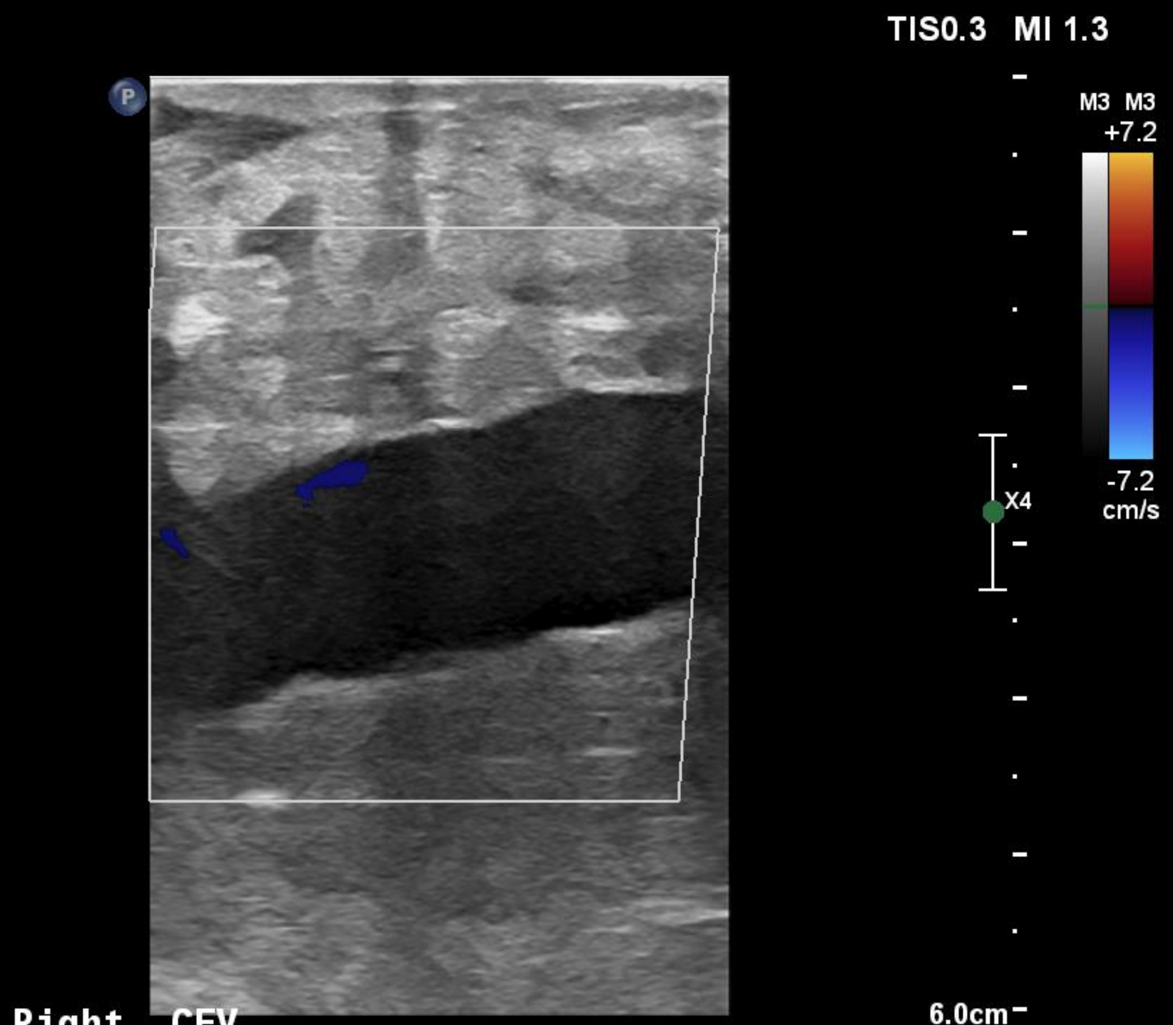
TIS0.3 MI 1.3
M3 M3
+7.2
-7.2
cm/s
X4
6.0cm
Right CFV
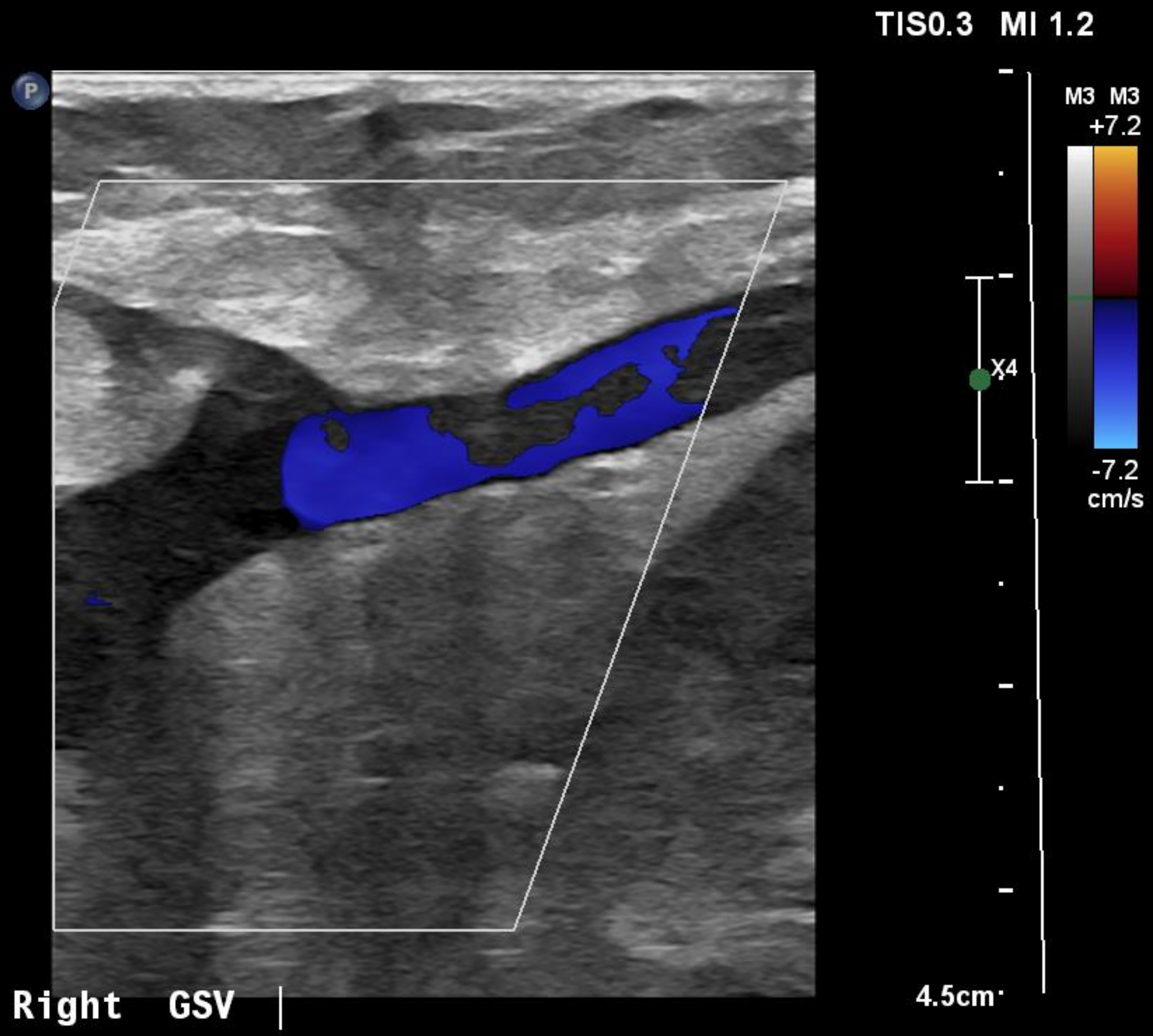
TIS0.3 MI 1.2
M3 M3
+7.2
-7.2
cm/s
X4
4.5cm
Right GSV

DVT
